# ﻿Fauna, distribution, and DNA barcoding data of caddisflies (Insecta, Trichoptera) in Croatia

**DOI:** 10.3897/zookeys.1263.152515

**Published:** 2025-12-10

**Authors:** Mladen Kučinić, Ana Previšić, Anđela Ćukušić, Ivan Vučković, Sanja Žalac, Darko Cerjanec, Renata Ćuk, Katarina Z. Stojanović, Nazymgul Akimbekova, Marijana Vuković, Josip Skejo, Dora Hlebec, Aleksandar Božić, Hrvoje Kutnjak

**Affiliations:** 1 Department of Biology, Faculty of Science, University of Zagreb, Horvatovac 102A, 10000 Zagreb, Croatia; 2 Collaborative Research Centre 1357 Microplastics, Faculty of Biology, Chemistry and Earth Sciences, University of Bayreuth, Universitätsstr. 30, 95447 Bayreuth, Germany; 3 Ministry of Economy and Sustainable Development, Ulica grada Vukovara 78, 10000 Zagreb, Croatia; 4 Elektroprojekt d.d., Civil and Architectural Engineering Department, Alexandera von Humboldta 4, 10000 Zagreb, Croatia; 5 ZSC ˝Dr. Ivo Pevalek˝, Plitvice Lakes National Park, Josipa Jovića 19, 53231 Plitvička Jezera, Croatia; 6 Primary School Barilović, Barilović 96, 47252 Barilović and Primary School Netretić, Netretić 1, 47271 Netretić, Croatia; 7 Josip Juraj Strossmayer Water Institute, Ulica grada Vukovara 220, 10000 Zagreb, Croatia; 8 Institute of Zoology, Faculty of Biology, University of Belgrade, Studentski trg 16 11000 Belgrade, Serbia; 9 Department of Biology and Ecology, Toraighyrov University, Lomov 64, 140000 Pavlodar, Kazakhstan; 10 Croatian Natural History Museum, Demetrova 1, 10000 Zagreb, Croatia; 11 Department of Evolutionary Biology, Ecology and Environmental Sciences, University of Barcelona, Avinguda Diagonal 643, 08028 Barcelona, Spain; 12 Faculty of Agriculture, University of Zagreb, Svetošimunska 25, 10000 Zagreb, Croatia

**Keywords:** Caddisfly, distribution, DNA barcoding, historical entomology, insect biodiversity, species checklist, subspecies differentiation

## Abstract

This study presents a thorough overview of the diversity of the Trichoptera fauna in Croatia, encompassing several key aspects. First, it offers a historical overview of Trichoptera research conducted within the country, tracing the development and major milestones of this field. Second, it provides a detailed analysis of the distribution and species diversity of caddisflies across Croatia’s three geographical regions, the Continental, Alpine, and Mediterranean, as well as within two ecoregions (ER5 – Dinaric Western Balkan and ER11 – Pannonian Lowlands) and two major river basins (AS – Adriatic Sea basin and BS – Black Sea basin). This biogeographic assessment is based on comprehensive records of adult specimens and, in certain cases, on DNA barcoding data obtained from larval stages. Third, the study includes a thorough examination of species synonyms and a critical review of the existing literature. Finally, it delivers a faunistic and taxonomic review of selected species and subspecies. A total of 225 species belonging to 18 families and 74 genera have been identified. Two of these species are represented by two and three subspecies, respectively, bringing the total number of recorded Trichoptera taxa in Croatia to 228. The presence of 223 species was confirmed in the adult stage. Only two species were identified by DNA barcoding as larvae. In the Continental region 170 species were recorded, in the Alpine region 155, and in the Mediterranean part 131 species. The Black Sea basin contains 203, and the Adriatic basin 141 species. In the Pannonian Lowland region (Ecoregion 11) we determined 152 and in the Dinaric Western Balkan (Ecoregion 5) 197 species. The BOLD Systems database currently contains about 593 DNA barcoded Trichoptera specimens from Croatia comprising 176 species, identifying 211 BINs, covering 78% of Croatian Trichoptera fauna.

## ﻿﻿Introduction

Systematic, long-term faunistic research within specific regions often results in comprehensive species checklists for various animal groups (Gumhalter et al. 2024; Holzenthal and Calor 2017; Nógardi and Uherkovich 2002; Petrović et al. 2014; Santos et al. 2020; Vilenica et al. 2015). These checklists are essential for assessing biodiversity, faunistic uniqueness, conservation priorities and advancing conservation biology (Petrović et al. 2014). Typically compiled at the national level, they provide valuable insights into regional biodiversity. Checklists of caddisflies have been developed for most European countries, including Greece (Malicky 2005a), Italy (Lodovici and Valle 2020), Estonia (Viidalepp et al. 2011), Ireland (O’Connor 2015), Serbia (Živić et al. 2006); outside Europe, there are, for example, Kazakhstan (Smirnova et al. 2016), Mongolia (Chuluunbat et al. 2022), and Brazil (Paprocki et al. 2004). Some of these checklists have been revised and updated multiple times, such as the one for Italy (Cianficconi 2002; Lodovici and Valle 2020; Moretti and Cianficconi 1981).

Caddisflies (Trichoptera) are one of the five exclusively aquatic insect orders (Holzenthal et al. 2007; Kjer et al. 2001); together with mayflies (Ephemeroptera) and stoneflies (Plecoptera), they form the EPT group, which are the cornerstone in freshwater biomonitoring (Šumanović et al. 2024). Together with Megaloptera, they belong to holometabolous aquatic insects, and are closely evolutionarily related to butterflies (Lepidoptera) (Frandsen et al. 2025; Holzenthal et al. 2007). Distributed across all continents except Antarctica (Frandsen et al. 2025), caddisflies are an integral component of aquatic ecosystems, inhabiting diverse freshwater habitats, with a few species adapted to brackish waters (Malicky 2005a). Additionally, a few species thrive in semi-terrestrial habitats like the genus *Enoicyla* (Glime 2017). Caddisfly larvae are highly adaptable, often building protective cases from pebbles, sand, aquatic vegetation, twigs, and even snail shells, while some are free-living. Their larval stages typically molt five times. Ecologically, they fill diverse roles as scrapers, filter feeders, shredders, miners, suckers, and predators (Hickin 1967; Sole and Gullefors 1996; Vitecek et al. 2017, 2020; Wallace et al. 2003), making them a key component of aquatic ecosystems.

The order Trichoptera encompasses 65 extant families and 17,279 described species, with approximately 300 fossil species from 22 families (Trichoptera World Checklist 2025). In Europe, as part of the Palearctic biogeographic region, between 1,700 and 1,800 caddisfly taxa have been documented (e.g., Botosaneanu and Malicky 1978; Ibrahimi 2024; Ibrahimi et al. 2016; Kumanski 1985, 1988, 2006; Malicky 2004; Malicky et al. 2007; Neu et al. 2018; Oláh et al. 2017, 2022a, 2022b; Valladolid et al. 2019, 2022, 2023; Vitecek et al. 2015). Between 2000 and 2020, 362 species of Trichoptera species were newly described in Europe (Sánchez-Campaña et al. 2023).

This study, based on three decades of research, synthesises the available literature on the Croatian Trichoptera fauna and re-examines both historical and newly established collections. It presents the first comprehensive checklist of Croatian caddisflies and includes: 1) a historical overview of Trichoptera research in the country; 2) an analysis of species distribution and diversity across Croatia’s three main geographical regions, two ecoregions, and two river basins, based on records of adult specimens or, in some cases, DNA barcodes of larval stages, including synonyms and critical evaluation of the literature; 3) a summary of DNA barcoded species from Croatia listed in the BOLD Systems database; 4) faunistic and taxonomic overview of selected species and subspecies.

### ﻿﻿Historical overview of Trichoptera research in Croatia

Trichopteran research in Croatia spans several distinct periods: 1) the initial investigations in the 19^th^ century; 2) the early 20^th^-century studies; 3) the limnological research beginning in the 1950s, and 4) the systematic faunistic, ecological, and taxonomic studies initiated in the 1990s, which continue to the present day.

In the early stages, three key caddisfly collections existed in Croatia: the Central Trichoptera Collection at the Croatian Natural History Museum in Zagreb, the Entomological Collection Košćec at the Varaždin City Museum (Malicky 2009), and the Collection Henč at the Faculty of Forestry, University of Zagreb. During the last 25 years, additional collections, such as the private Collections Cerjanec, Vučković, and Žalac, and the caddisfly collections at the Faculty of Science, University of Zagreb and at the Croatian Natural History Museum have significantly contributed to the knowledge of Croatia’s caddisfly fauna.

The study of caddisflies in Croatia began at the beginning of the 19^th^ century when the famous entomologist and mineralogist, professor of zoology and director of the Mineralogical Museum in Halle, Ernest F. Germar, collected insects in Dalmatia in 1811. In his list of insects from that area, he cited the species *Phryganea atrata* (Germar, 1817), a synonym of *Notidobia ciliaris* (Linnaeus, 1761) according to Guido Nonveiller (Nonveiller 1989). Later, the first data on caddisflies fauna were published in 1876 by the renowned entomologist Friedrich Brauer (Brauer 1876), followed by the Czech entomologist František Klapálek (1906) and Augustin Langhoffer (1912, 1915), professor at the University of Zagreb and curator of the Zoological Museum in Zagreb. In his two papers, Langhoffer reported information about seven caddisfly species collected in Croatian caves (Langhoffer 1912, 1915). The Czech entomologist František Klapálek published a pioneering study on Croatian caddisflies, reporting 38 species (Klapálek 1906) which constituted the first significant investigation of the caddisfly fauna in Croatia. The herpetologist and trichopterologist from Belgrade, Milutin Radovanović published in 1935 an extensive study on the fauna of caddisflies in former Yugoslavia, listing 21 species from Croatia (Radovanović 1935).

In the mid-20^th^ century, limnological studies advanced the understanding of caddisfly communities and the structure of macrozoobenthos, particularly through larval-stage analyses (Habdija 1979, 1988; Habdija et al. 1994; Matoničkin 1959, 1987; Matoničkin and Pavletić 1960, 1967; Matoničkin et al. 1971). Such research and the applied methodology (analysis of larval stages) has continued to the present day (Bućan and Miliša 2023; Habdija et al. 2003, 2004; Sviben et al. 2024; Šumanović et al. 2024; Vilenica et al. 2025; Vučković et al. 2009).

The collection of adult caddisflies in Croatia began in the 1970s, led by Mara Marinković-Gospodnetić. She investigated the springs of the Plitvice stream, and the rivers Crna Rijeka, Gacka, and Kostelka, recording 17 species for this Alpine area (Marinković-Gospodnetić 1971, 1979). Her most notable finding in these short-term research periods was the discovery of a new species to science, *Drusus croaticus* (Marinković-Gospodnetić, 1971), described from specimens collected in the National Park Plitvice Lakes.

In recent decades, Hans Malicky has made groundbreaking contributions, describing several endemic species, including *Athripsodes dalmatinus* Malicky, 1980 and *Chaetopteryx marinkovicae* Malicky & Krušnik, 1988 (Malicky 1980; Malicky and Krušnik 1988). Working through the caddisfly collection of the Natural History Museum in Vienna, Hans Malicky described the endemic species *Athripsodes dalmatinus* from specimens collected in the 1960s in Dalmatia, the Mediterranean part of Croatia (Malicky 1980). The same author together with the Slovenian trichopterologist Ciril Krušnik described the endemic species *Chaetopteryx marinkovicae* (Malicky and Krušnik 1988) from Istria (locality: Ročko polje, Kompanj, Ugrin). At the end of the last century, systematic collections of caddisflies in Croatian caves began, contributing detailed knowledge to that segment of our fauna and its basic ecological and ethological characteristics (Gottstein Matočec et al. 2002; Kučinić and Ilić 1993). One of the results of these studies is the first collection of cave Trichoptera in the Varaždin City Museum.

Malicky provided interesting information about the Croatian caddisfly fauna in the work presented at the symposium on the occasion of the 95^th^ anniversary of Zdravko Lorković’s birthday (Malicky 1996). The same author, together with Slovenian entomologists Ciril Krušnik and Gorazd Urbanič, described a new species *Rhyacophila schmidinarica* Urbanič, Krušnik & Malicky, 2000 from the Balkan peninsula including the locality Brušane in the Lika region, Croatia (Urbanič et al. 2000). Later on, this species was found and confirmed by findings in the National park Plitvice Lakes (e.g. Kučinić 2000; Malicky 2014) and another part of Croatia (e.g. Cerjanec et al. 2020; Previšić et al. 2013c). Professor Malicky also redetermined caddisflies collected by Franjo Košćec in the 1930s (collection Košćec of the Varaždin City Museum), which consists of 39 caddisfly species, ten of which were new records for the Croatian fauna (Malicky 2009).

During the past three decades, continuous systematic research on Trichoptera in Croatia, primarily focusing on adults, has significantly advanced the knowledge of this group. During this period, caddisflies were collected from 278 localities (Suppl. material 1), culminating in the establishment of several Trichoptera collections. This research has contributed to the completion of six doctoral theses (Cerjanec 2012; Ćukušić 2019; Kučinić 2002; Previšić 2009; Vučković 2011; Žalac 2022) and the publication of more than 65 scientific papers exploring diverse aspects of Trichoptera biology including DNA barcoding, ethology, phylogeny, distribution, diversity, and taxonomy (e.g., Ćukušić 2019; Previšić et al. 2014a; Waringer et al. 2009).

The first systematic studies of Trichoptera in Croatia were conducted in the Plitvice Lakes National Park (the Alpine part of Croatia), focusing on adult specimens. These investigations identified 91 species, approximately 40 of which were recorded for the first time in the Croatian fauna (e.g., Ivković et al. 2013, 2023; Kučinić 2002; Kučinić et al. 2017b; Malicky 2014a; Previšić et al. 2007a, 2010, 2013b; Šemnički et al. 2011, 2012; Žalac and Kučinić 2022). Notably, the larva of the previously unknown species *Drusus croaticus* Marinković-Gospodnetić, 1971 was described from this region (Kučinić et al. 2008). Research into the emergence patterns of caddisflies in this area is currently ongoing (Ivković et al. 2013; Previšić et al. 2007a; Šemnički et al. 2011).

In the Alpine part of Croatia, research was further conducted in the Gorski kotar region, including the rivers Dobra, Čabranka, Kupa, Kamačnik, and also Plitvice Lakes National Park (e.g., Cerjanec 2012; Cerjanec et al. 2020; Ćuk and Vučković 2014; Ćuk et al. 2015; Ćukušić 2019; Habdija et al. 1994; Ivković et al. 2013; Kučinić 2002; Kučinić et al. 2008, 2015, 2017b; Malicky 2014a; Malicky et al. 2007; Marinković-Gospodnetić 1971, 1979; Previšić 2009; Previšić and Popijač 2010; Previšić et al. 2007a, 2009, 2010, 2012, 2013b, 2014a, 2014b; Šemnički et al. 2011, 2012; Vučković et al. 2011, 2016; Žalac 2022; Žalac and Kučinić 2022).

In the Mediterranean region, studies have focused on the faunal characteristics and distribution of caddisflies in the Cetina, Krka and Neretva river basins, the southernmost area of Konavle, and the Croatian islands (e.g., Ćukušić 2019; Graf et al. 2008; Karaouzas et al. 2015; Kladarić et al. 2021; Kučinić et al. 2017a, 2019b, 2020a, 2020c; Malicky 2005a, 2014a; Oláh 2010; Previšić et al. 2014a; Ridl et al. 2017; Vučković 2011; Vučković et al. 2011, 2016, 2021; Waringer et al. 2009; Žalac 2022; Žalac and Kučinić 2022). Meanwhile, in the continental part of Croatia, research has been conducted in regions such as the Drava River (Previšić et al. 2007b), Sava River basin (Ćuk and Vučković 2010; Oláh et al. 2012; Szivák et al. 2017), Odra River (Ćuk et al. 2021), Mt. Ivanščica (Kladarić et al. 2021), Papuk Nature Park (Malicky 2014b; Oláh et al. 2012, 2015; Previšić et al. 2013c; Vučković et al. 2016; Szivák et al. 2017), Mt. Žumberak (Ćuk and Vučković 2009; Kučinić et al. 2015), Kopački rit Nature Park (Vrućina et al. 2016), Banovina region (Kučinić et al. 2010, 2013, 2020b; Szivák et al. 2017), and the Kordun region (Cerjanec 2012; Cerjanec et al. 2020).

During the past decade, systematic DNA barcoding (Hebert et al. 2003a, 2003b; Hogg et al. 2009; Ratnasingham and Hebert 2013; Ratnasingham et al. 2024; Santos et al. 2016) has further enriched the study of the Croatian caddisfly fauna, resulting in numerous publications (e.g., Ćuk et al. 2021; Ćukušić 2019; Ćukušić et al. 2017; Kladarić et al. 2021; Kučinić et al. 2017a, 2019b, 2019c, 2020c; Szivák et al. 2017) and in the identification of new or previously unrecognized species (e.g., Ćukušić 2019; Ćukušić et al. 2017; Kladarić et al. 2021; Kučinić et al. 2013, 2020a; Szivák et al. 2017; Valladolid et al. 2020).

During the last 15 years, some caddisfly species have been described from Croatia, showcasing the region’s biodiversity. These include *Rhyacophila delici* Kučinić & Valladolid, 2020, found across various Croatian regions (Valladolid et al. 2020), and species from the continental region, such as *Chaetopteryx bucari* Kučinić, Szivák & Delić, 2013 and *Chaetopteryx uherkovici* Oláh, 2011 (Kučinić et al. 2013; Oláh 2011). Other notable discoveries include *Rhyacophila cabrankensis* Malicky, Previšić & Kučinić, 2007, recorded exclusively at the spring of the Čabranka River in the Alpine region (Malicky et al. 2007), and *Tinodes andrasi* Oláh, 2010, documented in southeastern Croatia (Oláh 2010). Additionally, *Ecclisopteryx ivkae* Previšić, Graf & Vitecek, 2014 was identified in the central Mediterranean area (Previšić et al. 2014a) in the upper part of of the Cetina River (Previšić et al. 2014a; Vučković et al. 2016).

Larvae have also been described for several species, including Dinaric endemics: *Anitella apfelbecki* Klapálek, 1899 (Waringer et al. 2009); *Drusus croaticus*, also an endemic species of the Dinaric karst (Alpine region; Kučinić et al. 2008); *Rhyacophila balcanica* Radovanović, 1953, a Mediterranean species whose larvae were collected and described from Greece, Montenegro, and Croatia (Karaouzas et al. 2015), *Tinodes braueri* McLachlan, 1878, whose larva was identified in the Mediterranean part of Croatia (Graf et al. 2008); and the Croatian endemic *Ecclisopteryx ivkae*, described also from the Mediterranean part of Croatia (Previšić et al. 2014a). Recently, Croatia has also become a centre for ecological and ecotoxicological research, especially on pharmaceutical transfer across aquatic–terrestrial boundaries using Trichoptera as a model (e.g., Veseli et al. 2022, 2024).

## ﻿﻿Materials and methods

### ﻿﻿Geographical, climatic, and hydrological characteristics of Croatia

**Figure 1.**
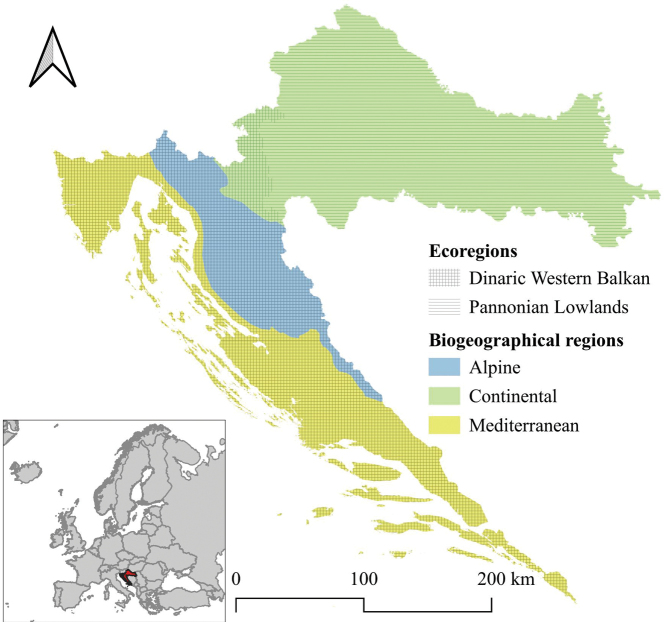
Map of Croatia showing its position in Europe, three biogeographical regions, and two ecoregions.

The Republic of Croatia is situated at the intersection of Central and Southeastern Europe, and belongs both to the Central European and Mediterranean biogeographical regions. It spans approximately 56,000 km^2^ of land and 28,000 km^2^ of marine territory and includes more than 1,100 islands scattered along its Adriatic coastline. Based on its climatological, geological, vegetational, and geographical characteristics, Croatia can be divided into three primary regions: Pannonian-Peripannonian = Continental region or part, Central–Mountainous = Alpine region or part and the Mediterranean region or part (Fig. 1) (Bertić et al. 2001). The Continental region, covering the northern and eastern parts of the country, is characterised by a temperate continental climate and features a dense network of both lotic (rivers, streams) and lentic (lakes, ponds) freshwater habitats. The Alpine region, centrally located and the smallest of the three, is distinguished by karstic terrain, numerous springs, mountain brooks, and short rivers flowing through forested, mountainous landscapes (within this region, we included the peaks of Mt. Dinara). Finally, the Mediterranean region, which extends along the southern and southeastern Adriatic coast, including the Dalmatian hinterland and islands, is the longest in geographic extent. Except for the Istrian Peninsula, it is relatively poor in surface watercourses and is dominated by intermittent streams and isolated rivers. This region experiences a typical Mediterranean climate, with hot, dry summers and mild, wet winters, causing many smaller watercourses to dry out during the summer months.

According to Illies (1978), Croatia is classified into two ecoregions (Fig. 1): the Dinaric Western Balkan – ecoregion 5 (ER5) and the Pannonian Lowland – ecoregion 11 (ER11) (Illies 1978; Vilenica et al. 2015).

**Figure 2.**
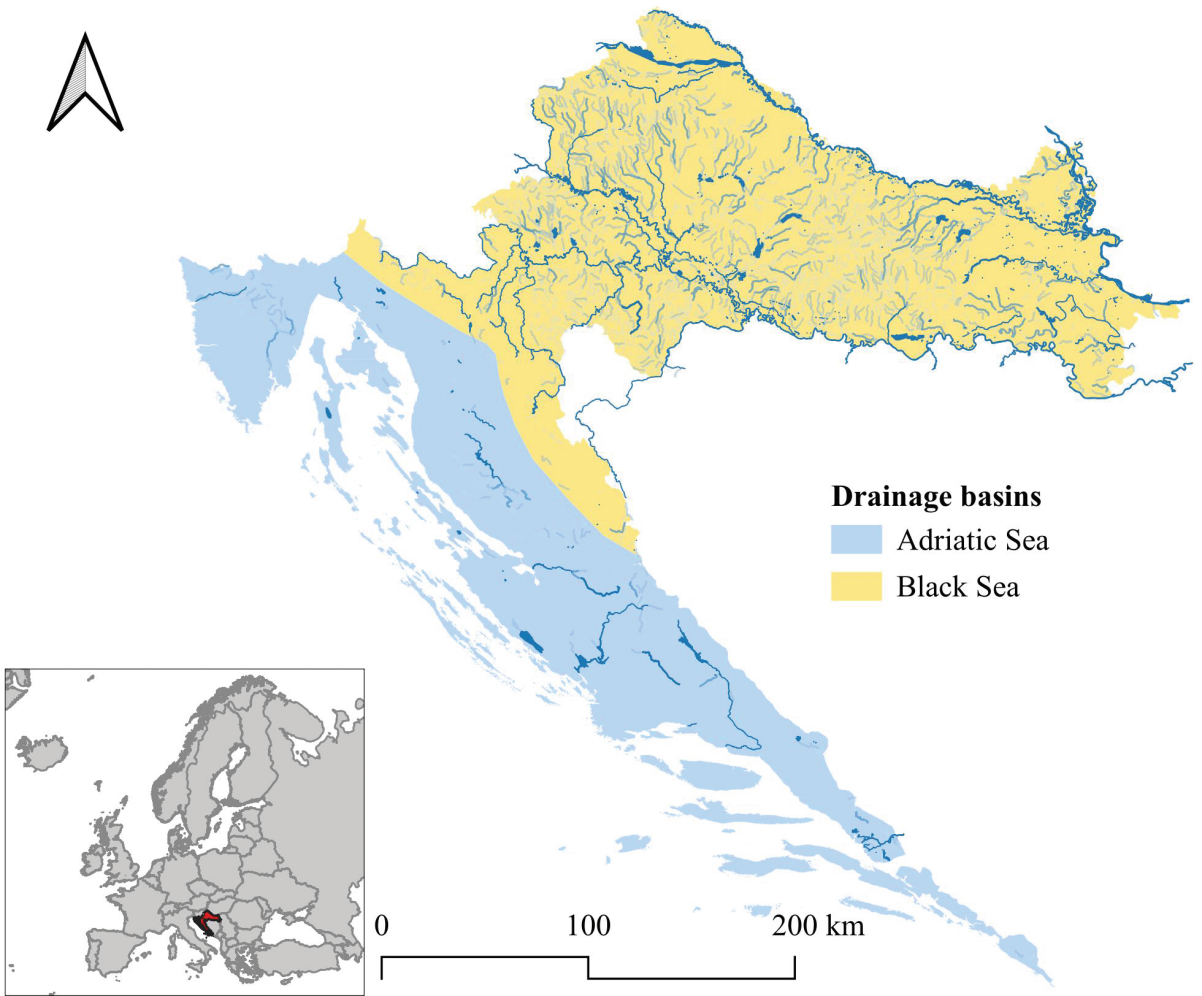
Hydrological map of Croatia showing the two drainage basins: the Black Sea Basin and the Adriatic Sea Basin, and its position in Europe.

Croatia is hydrologically divided into two major river basins (Fig. 2): the Black Sea Basin (BS), which includes watercourses in the Pannonian-Peripannonian region (continental) and the mountain (Alpine) region, and the Adriatic Sea basin (AS) which includes watercourses in the Mediterranean and Alpine regions (Habdija and Primc 2019; Kučinić et al. 2020a; Vilenica et al. 2015). All continental watercourses belong to the Black Sea basin, with the largest rivers Sava, Drava and Danube. The Adriatic Basin comprises isolated rivers such as Rječina, Zrmanja, Krka, Cetina, the lower part of the Neretva, as well as the Ljuta River in the far south of Croatia, which drain directly into the Adriatic Sea and some rivers from the Alpine region, e.g., Lika, Gacka, and Kostelka rivers. The Adriatic islands are relatively poor in inland water resources. Only a few islands have lakes (e.g., Krk, Pag and Cres) or streams (e.g., Krk, Pag, Rab), many of which are intermittent.

**Figure 3.**
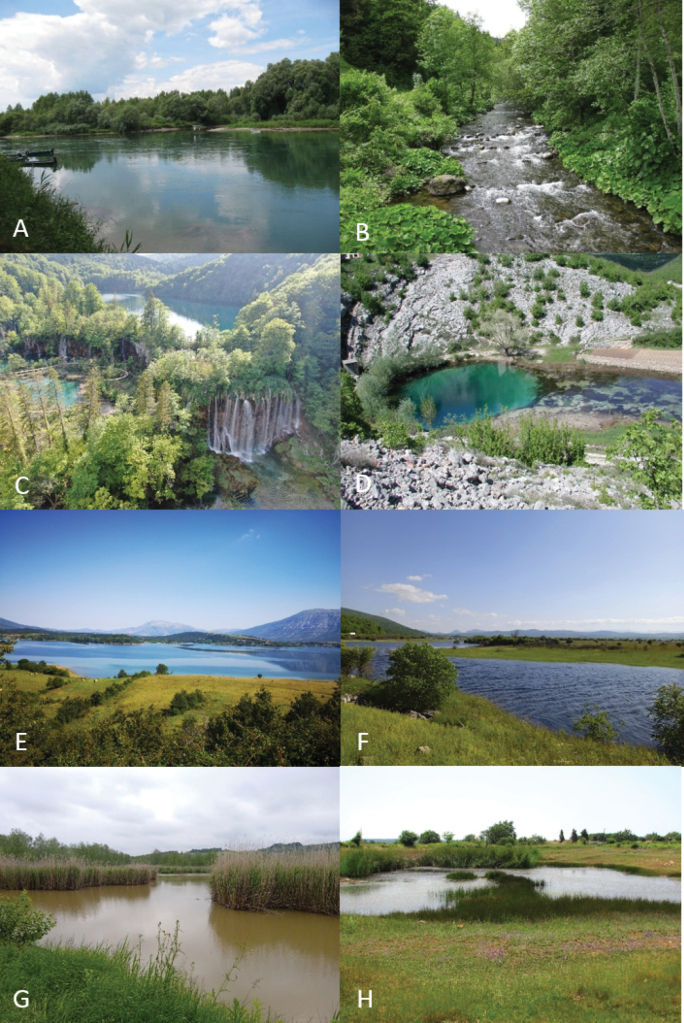
Aquatic habitats in Croatia. A. River Drava at Legrad (Continental part of Croatia); B. Upper part of the River Čabranka (Alpine part of Croatia); C. National Park Plitvice Lakes, tuffa barriers (Alpine part of Croatia); D. Source of the River Cetina (Mediterranean part of Croatia); E. Peruča lake accumulation (Mediterranean part of Croatia); F. Temporary flooded karst Krbavsko polje, Lika (Alpine part of Croatia); G. Fishpond near Konjščina, Hrvatsko zagorje (Continental part of Croatia); H. Temporary pond on the island of Pag (Mediterranean part of Croatia).

Croatia is characterised by a wide spectrum of aquatic habitats - springs, mountain streams, rivers, ponds, peat bogs, and lakes, including reservoir lakes, etc., supporting a rich aquatic fauna (Fig. 3A–H).

### ﻿﻿Field research

The collection of Trichoptera was carried out throughout Croatia in all three geographical regions, at 278 localities (Fig. 3A–H; Suppl. material 1). Adults were mainly collected using 15W UV lamps. Initially, portable batteries were used for fieldwork, and later smaller, more easily portable batteries were employed. In addition to UV lamps, Trichoptera were also collected using emergence pyramid traps (e.g., Ivković et al. 2013; Kučinić 2002; Kučinić et al. 2013, 2017b; Previšić et al. 2007a; Ridl et al. 2017) and entomological net. All collected material was stored in 80% ethanol, while for certain molecular analyses, primarily DNA barcoding, in absolute ethanol. Older collections, such as the ‘Collection Koščec’ and the Trichoptera collection at the Croatian Natural History Museum, consist of specimens mounted on entomological pins (Malicky 2009).

### ﻿﻿Laboratory work

Challenges in identifying larvae to species level arise from the lack of distinct morphological features for certain species and the fact that some species remain undescribed (Waringer and Graf 2011). Consequently, adult specimens were prioritised for inclusion in this study, as adult collection is the standard method for biodiversity and faunistic studies on caddisflies (e.g., Ibrahimi et al. 2012; Kučinić et al. 2015, 2019c, 2021; Malicky 2014a; Marinković Gospodnetić 1971, 1979; Musliu et al. 2024; Nuntakwang et al. 2006; Previšić et al. 2007a, 2007b; Svensson 1972, 1974). Exceptions were made for recent studies employing DNA barcoding and detailed larval morphological analyses (e.g., Ćuk et al. 2021; Ćukušić 2019; Kladarić et al. 2021).

Species identification was conducted using standard taxonomic keys (Malicky 1983, 2004; Kumanski 1985, 1988) and recent taxonomic literature (e.g., Kučinić et al. 2013; Malicky et al. 2007; Neu 2015; Oláh 2010, 2011; Previšić et al. 2014a; Oláh et al. 2015, 2017, 2021, 2022a; Valladolid et al. 2020). When a taxon (species/subspecies) is mentioned for the first time, it is listed with its full Latin name with author and year of description.

A comparison of the faunistic similarity among the three geographic regions (Fig. 1) was conducted using the Sørensen Index (Sørensen 1948).

### ﻿﻿DNA barcoding

The protocol for DNA barcoding of Croatian Trichoptera specimens is detailed in numerous recent publications (Ćukušić 2019; Ćukušić et al. 2017; Kladarić et al. 2021; Szivák et al. 2017; Valladolid et al. 2020). More than 85% of DNA-barcoded specimens from Croatia were collected, processed, and entered into the BOLD Systems database by the authors of this study, resulting in the establishment of the DNA barcoded Trichoptera Collection NOVA at the Faculty of Science, University of Zagreb. Around 85% of the DNA-barcoded species were analysed as a part of the “DNA barcoding of biodiversity of Croatian fauna” project. The doctoral thesis of Anđela Ćukušić was the first to present DNA barcoding of Trichoptera in Croatia (Ćukušić 2019). In addition to Croatia, preparation of samples for DNA barcoding and DNA barcoding of specimens collected in Croatia was also performed in laboratories in Germany, Hungary, Serbia, Spain, and the USA. DNA barcoding of 593 Trichoptera specimens from Croatia in the BOLD database (The Barcode of Life Data Systems, versions 3 and 5) is shown in Suppl. material 2, with data on localities, geocoordinates, and geographical regions.

### ﻿﻿Systematic overview and data sources

The systematic presentation and synonyms follow the Trichoptera World Checklist (2025). Families within suborders and species and genera within families are given alphabetically. For each species included in the systematic list, the following data are provided: 1) scientific name; 2) synonyms according to Trichoptera World Checklist (2025); 3) distribution across the three biogeographical regions in Croatia (Continental, Alpine, and Mediterranean); 4) general distribution according to Neu at al. (2018); 5) records in ecoregions (Dinaric Western Balkan - ER5 and Pannonian Lowland - ER11) and in the river basins (Black Sea – BS and Adriatic Sea - AS); 6) DNA barcoding information from the BOLD database; and 7) literature data.

The data used to determine the biodiversity and distribution of Trichoptera across the three geographic regions of Croatia were obtained through the analysis of the following Trichoptera collections and literature sources.

Continental region: Collections of the Faculty of Science, University of Zagreb, collections of the Croatian Natural History Museum in Zagreb, the Koščec Collection from the Municipal Museum in Varaždin, private collection of Darko Cerjanec. Literature sources: Ćuk and Vučković (2009, 2010, 2014); Ćuk et al. (2021); Ćukušić (2019); Ćukušić et al. (2017); Gottstein Matočec et al. (2002); Kladarić et al. (2021); Klapálek (1906); Kučinić and Ilić (1993); Kučinić et al. (2010, 2013, 2015, 2019a, 2020a, 2020b); Langhoffer (1912, 1915); Malicky (1996, 2009, 2014b); Neu et al. (2018); Oláh (2010, 2011); Oláh et al. (2012, 2015, 2022b); Previšić et al. (2007b, 2013a, 2013c); Radovanović (1935), Szivák et al. (2017); Valladolid et al. (2020); Vrućina et al. (2016);

Alpine region: Collections of the Faculty of Science, University of Zagreb, collections of the Croatian Natural History Museum in Zagreb, private collections of Sanja Žalac and Darko Cerjanec. Literature sources: Cerjanec et al. (2020); Gottstein Matočec et al. (2002); Ćuk et al. (2015); Ćukušić (2019); Ivković et al. (2013); Kladarić et al. (2021); Klapálek (1906); Kučinić (2002); Kučinić and Ilić (1993); Kučinić and Malicky (2002); Kučinić et al. (2014, 2015, 2017b, 2019a, 2020a); Langhoffer (1912, 1915); Malicky (2014a); Malicky et al. (2007); Marinković-Gospodnetić (1971, 1977, 1979); Neu et al. (2018); Oláh (2010); Oláh et al. (2013, 2017, 2018, 2021, 2022b); Previšić and Popijač (2010); Previšić et al. (2007a, 2009, 2010, 2012, 2013b, 2014a, 2014b); Radovanović (1935); Šemnički et al. (2011, 2012); Valladolid et al. (2020, 2022); Žalac and Kučinić (2022);

Mediterranean region: Collections of the Faculty of Science, University of Zagreb, collections of the Croatian Natural History Museum in Zagreb, private collections of Ivan Vučković and Sanja Žalac. Literature sources: Ćukušić (2019); Ćukušić et al. (2017); Germar (1817); Gottstein Matočec et al. (2002); Graf et al. (2008); Karaouzas et al. (2015); Kladarić (2021); Klapálek (1906); Kučinić and Ilić (1993); Kučinić et al. (2011, 2015, 2016, 2017a, 2019a, 2019b, 2019c, 2020a, 2020c, 2021); Malicky (1979, 2005a, 2014a); Malicky and Krušnik (1988); Neu et al. (2018); Oláh (2010); Oláh and Vinçon (2023); Oláh et al. (2017); Previšić et al. (2014a); Radovanović (1935); Ridl et al. (2017); Vučković et al. (2011, 2016, 2021); Valladolid et al. (2020, 2022); Waringer et al. (2009); Žalac and Kučinić (2022).

The general distribution of species is presented in the same manner as the Distribution Atlas of European Trichoptera by Neu et al. (2018). The distribution of some species is specified by country when their range is restricted. However, if a species is present in many European countries, it is referred to as widely distributed. In addition, distributions covering countries that belong to a specific geographical region of Europe are grouped under a regional name. For example, when a species occurs in multiple Alpine countries (southeastern France, Switzerland, Bavaria, Liechtenstein, Austria, Italy and Slovenia), its distribution is referred to as the Alpine region. Similarly, wide distributions within the Balkan or Scandinavian countries are unified under the terms Balkan Peninsula and Scandinavian Peninsula, respectively. Regarding Italy, if a species is broadly distributed across the mainland (excluding the Italian Alps and islands), its range is indicated as the Apennine Peninsula. If it occurs only in a part of Italy, the distribution is listed simply as Italy. The same approach is applied to species distributed in the Balkan or Scandinavian regions.

## ﻿﻿Results

This study provides an overview of the diversity of the Trichoptera fauna of Croatia in the form of a checklist. Altogether, 225 species were identified from 18 families and 74 genera (Table 1): Annulipalpia, 48 species (5 families, 14 genera) and Integripalpia, 177 species (13 families, 60 genera). Two species, *Rhyacophila dorsalis* (Curtis, 1834) and *Potamophylax cingulatus* (Stephens, 1837) were recorded with two subspecies (*R. dorsalis persimilis* McLachlan, 1879 and *R. dorsalis plitvicensis* Kučinić & Malicky, 2002), and three subspecies (*P. cingulatus cingulatus* (Stephens, 1837), *P. cingulatus alpinus* Tobias, 1994 and *P. cingulatus depilis* Szczęsny, 1994), so the overall number of taxa is 228. For the first time, *Microptila minutissima* was recorded in Croatia with the exact location of the collected adults (locality Zeleni Vir, Alpine part of Croatia, leg. W. Graf). The most species-rich family Limnephilidae, contains 66 identified species (Table 1), but several families contain only one species, e.g., Apataniidae, Odontoceridae (Table 1).

**Table 1.** Number of caddisfly genera and species by families, in the three geographical regions of Croatia.

Family	Croatia genus / species	Continental part genus / species	Alpine part genus / species	Mediterranean part genus / species
Ecnomidae	1 / 1	1 / 1	1 / 1	1 / 1
Hydropsychidae	3 / 16	3 /14	2 / 8	3 / 10
Philopotamidae	2 / 7	2 / 7	2 / 6	1 / 2
Polycentropodidae	5 / 12	5 / 11	4 / 9	4 / 8
Psychomyiidae	3 / 12	3 / 7	3 / 9	3 / 11
Apataniidae	1 / 1	-	1 / 1	-
Beraeidae	4 / 7	2 / 4	4 / 6	3 / 4
Brachycentridae	2 / 5	2 / 2	2 / 5	-
Glossosomatidae	3 / 9	3 / 8	3 / 6	3 / 6
Goeridae	3 / 6	3 / 6	3 / 6	1 / 3
Hydroptilidae	7 / 22	5 / 15	6 / 14	6 / 14
Lepidostomatidae	2 / 4	2 / 3	2 / 3	1 / 2
Leptoceridae	9 / 30	8 / 25	6 / 16	8 / 21
Limnephilidae	19 / 66	15 / 43	15 / 46	13 / 38
Odontoceridae	1 / 1	1 / 1	1 / 1	1 / 1
Phryganeidae	6 / 7	6 / 7	4 / 5	3 / 3
Rhyacophilidae	1 / 17	1 / 14	1 / 11	1 / 5
Sericostomatidae	2 / 2	2 / 2	2 / 2	2 / 2
	**74 / 225**	**64 / 170**	**62 / 155**	**54 / 131**

In the Continental part of Croatia, 170 species from 17 families and 64 genera were determined, in Alpine 155 species, 62 genera and 18 families, and in Mediterranean, 131 species from 54 genera and 16 families (Table 1). In the area of Black Sea basin (BS) we determined 203 species and two subspecies (205 taxa), and in the Adriatic Sea basin (AS) 141 species. In the Pannonian lowland region (ER11) 152 species were determined, and in the Dinaric Western Balkans (ER5) 197 species and two subspecies (199 taxa). A total of 75 species (33% of our fauna) was registered only in one Croatian region: in the Continental part 35 species, in the Alpine part 20, and in the Mediterranean part 20 species. Altogether, 77 species (34% of Croatia caddisfly fauna) have been recorded in each of the three regions of Croatia. The highest similarity (118 shared species, 73% similarity) was recorded between the Continental and Alpine regions, whereas the lowest was between the Continental and Mediterranean regions (94 shared species, 62% similarity of caddisfly fauna) (Table 2).

**Table 2.** Sørensen (1948) similarity of Trichoptera fauna among the three geographical regions of Croatia.

	Continental part	Alpine part	Mediterranean part
**Continental part**	–	73%	62%
**Alpine part**	73%	–	66%
**Mediterranean part**	62%	66%	–

**Figure 4.**
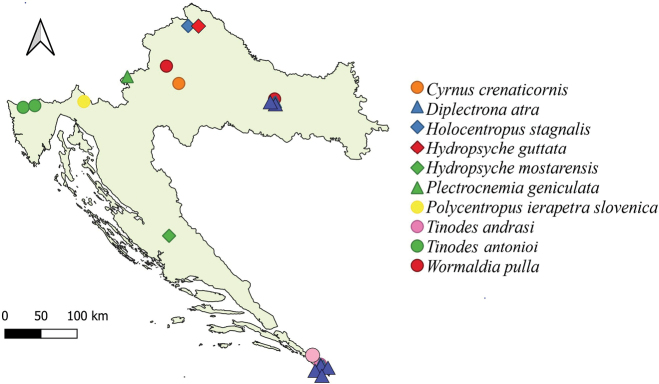
Map showing findings of 10 selected rare Annulipalpia species.

**Figure 5.**
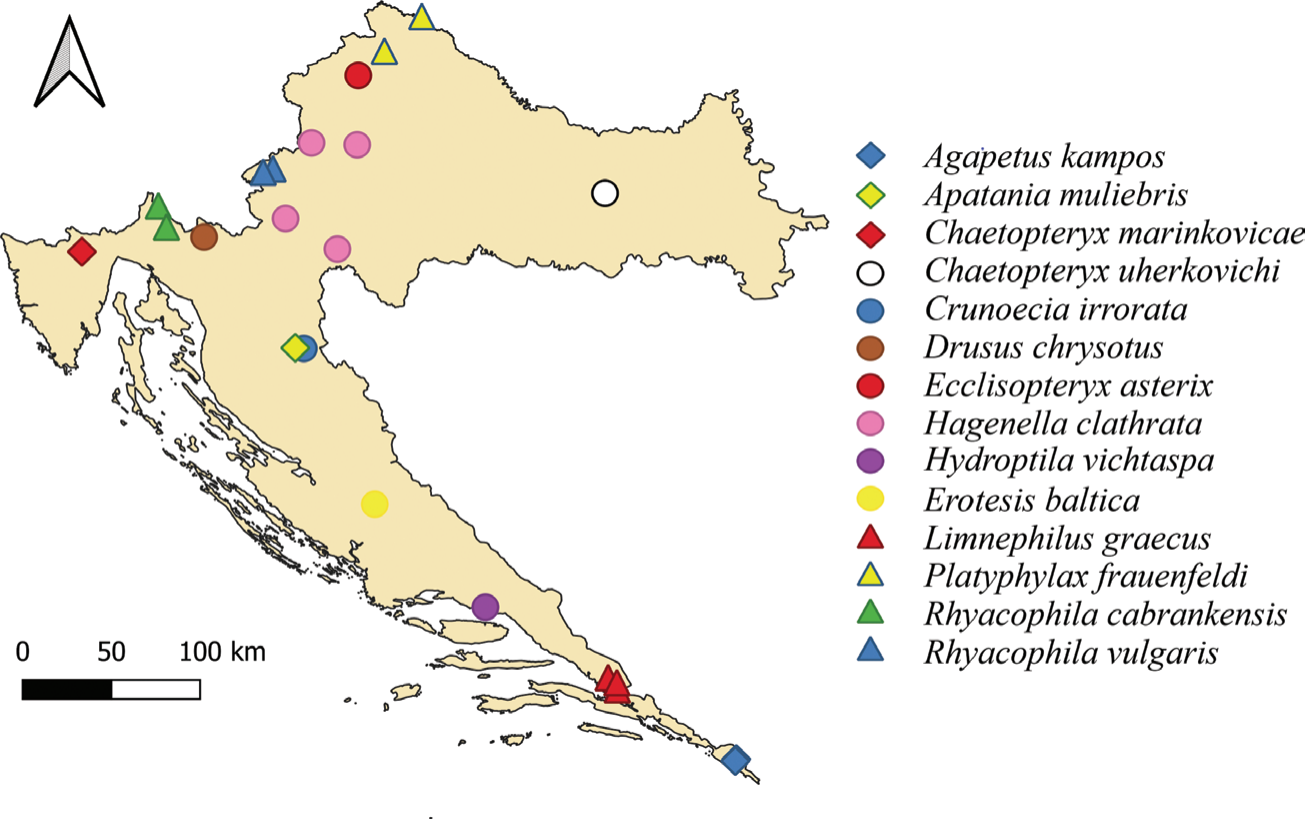
Map showing findings of 14 selected rare Integripalpia species.

Maps with findings of selected, rare species from suborders Annulipalpia (10 species) (Fig. 4) and Integripalpia (Fig. 5) (14 species) are also given as a part of this study.

In the BOLD Systems database, we analysed data from 593 specimens of caddisflies collected in Croatia (Suppl. material 2). These data represent 176 species (78% of the Croatian caddisfly fauna) with 211 BINs from the country.

### ﻿﻿Systematic checklist of caddisfly fauna in Croatia

Including synonyms, data on distribution in Croatia (Continental, Alpine, Mediterranean), ecoregions (ER5 - Dinaric Western Balkan, ER11 - Pannonian Lowlands), general distribution, drainages (AS - Adriatic Sea basin, BS - Black Sea basin), BOLD Systems database (BOLD ID), and literature data.

#### ﻿﻿Ordo TRICHOPTERA Kirby, 1813

﻿**Suborder Annulipalpia Martynov, 1924**

﻿**Family Ecnomidae Ulmer, 1903**

**Genus *Ecnomus* McLachlan, 1864**

***Ecnomus tenellus* (Rambur, 1842)**

**Synonyms.***Ecnomus falcatus* Mosely, 1932; *Ecnomus kososiensis* Kobayashi, 1987; *Ecnomus omiensis* Tsuda, 1942.

**Distribution in Croatia.** Continental part, Alpine part, Mediterranean part.

**General distribution.** Widely distributed in Europe. The species is also recorded in: China, Russian Far East, Nepal, Taiwan, and Vietnam.

**Ecoregion.**ER5, ER11.

**Drainage.**AS, BS.

**BOLD ID.**CROAA085-18 (Mediterranean part), CROAA107-18 (Alpine part), CROTR212-19 (Mediterranean part), CROTR234-19 (Mediterranean part).

**Literature data.**Cerjanec et al. (2020); Ćukušić (2019); Ivković et al. (2023); Kučinić et al. (2011, 2017b, 2019b, 2019c); Malicky (2009, 2014a); Neu et al. (2018); Previšić et al. (2007b); Ridl et al. (2017); Šemnički et al. (2011, 2012).

#### ﻿﻿Family Hydropsychidae Curtis, 1835

**Genus *Cheumatopsyche* Wallengren, 1891**

***Cheumatopsyche lepida* (Pictet, 1834)**

**Synonyms.***Tinodes albipunctatus* Stephens, 1836; *Hydropsyche angustata* Pictet, 1834; *Hydropsychodes atlantis* Navas, 1930; Catagapetus ? nigra Navas, 1916; *Hydropsyche varia* Rambur, 1842; *Hydropsyche ventralis* Curtis, 1836.

**Distribution in Croatia.** Continental part, Alpine part, Mediterranean part.

**General distribution.** Widely distributed in Europe, including European part of Russia (along the Ural Mountains). The species is also recorded in Algeria, Armenia, Georgia, Iran, Lebanon, Morocco, Syria, Tunisia, and Turkey.

**Ecoregion.**ER5, ER11.

**Drainage.**AS, BS.

**BOLD ID.**CROAA051-18 (Mediterranean part).

**Literature data.**Cerjanec et al. (2020); Ćukušić (2019); Kučinić et al. (2010, 2011, 2020b); Neu et al. (2018).

#### ﻿Genus *Diplectrona* Westwood, 1840

***Diplectrona atra* McLachlan, 1878**

**Synonym.***Hydropsyche hellenica* Jacquemart, 1957.

**Distribution in Croatia.** Continental part, Mediterranean part.

**General distribution.** Balkan Peninsula, France, Italy (Alps), Sardinia, Switzerland (Alps). The species is also recorded in Turkey.

**Ecoregion.**ER5, ER11.

**Drainage.**AS, BS.

**BOLD ID.**CROTR247-19 (Mediterranean part), CROTR291-19 (Mediterranean part), CROTR292-19 (Mediterranean part), CROTR293-19 (Mediterranean part), CROTR294-19 (Mediterranean part), CROTR295-19 (Mediterranean part), CROTR296-19 (Continental part), CROTR297-19 (Continental part), CROTR298-19 (Continental part), CROTR299-19 (Continental part), CROTR300-19 (Continental part), CROTR301-19 (Continental part), CROTR302-19 (Continental part).

**Literature data.**Ćukušić (2019); Kučinić et al. (2021); Previšić et al. (2013c); Žalac and Kučinić (2022).

#### ﻿Genus *Hydropsyche* Pictet, 1834

***Hydropsyche angustipennis* (Curtis, 1834)**

**Synonyms.***Hydropsyche* aspersa Rambur, 1842; *Hydropsyche fulvipes* Stephens, 1836; *Hydropsyche longipennis* Debauche, 1935, *Hydropsyche nebulosa* Pictet, 1834.

**Distribution in Croatia.** Continental part, Mediterranean part.

**General distribution.** Widely distributed in Europe, including European part of Russia. The species is also recorded in Siberia.

**Ecoregion.**ER5, ER11.

**Drainage.**AS, BS.

**BOLD ID.**CROAA108-18 (Mediterranean part), CROAA109-18 (Mediterranean part), CROAA119-18 (Continental part).

**Literature data.**Ćukušić (2019); Kučinić et al. (2011, 2021); Neu et al. (2018); Previšić et al. (2007b).

***Hydropsyche bulbifera* McLachlan, 1878**

**Synonyms.***Hydropsyche atrata* Navas, 1933; *Hydropsyche fallaciosa* Kumanski & Botosaneanu, 1974; *Hydropsyche mosulensis* Mosely, 1934; *Hydropsyche nebulosa* Brauer (nec Pictet), 1857; *Hydropsyche protecta* Navas, 1923; *Hydropsyche subguttata* Schmid (nec Martynov), 1959, not Martynov, 1927.

**Distribution in Croatia.** Continental part, Alpine part.

**General distribution.** Austria, Balkan Peninsula, Czechia, France, Germany, Iberian Peninsula, Poland, Slovakia, Switzerland, Ukraine. The species is also recorded in Iran and Turkey.

**Ecoregion.**ER5, ER11.

**Drainage.**AS, BS.

**BOLD ID.**CROAA005-18 (Continental part), CROTR039-19 (Alpine part).

**Literature data.**Ćukušić (2019); Kučinić et al. (2019a); Neu et al. (2018).

***Hydropsyche bulgaromanorum* Malicky, 1977**

**Distribution in Croatia.** Continental part, Alpine part.

**General distribution.** Austria, Belarus, Benelux, Bulgaria, Crimean Peninsula, Croatia, Czechia, Estonia, Finland, France, Germany, Great Britain, Hungary, Italy (Alps), Lithuania, Moldova, Poland, Romania, Serbia, Slovakia, Switzerland, Ukraine, including European part of Russia. The species is also recorded in Mongolia.

**Ecoregion.**ER5, ER11.

**Drainage.**AS, BS.

**BOLD ID.**CROAA055-18 (Continental part), CROAA056-18 (Continental part).

**Literature data.**Ćukušić (2019); Malicky (2009); Neu et al. (2018); Žalac and Kučinić (2022).

***Hydropsyche contubernalis* McLachlan, 1865**

**Distribution in Croatia.** Continental part.

**General distribution.** Widely distributed in Europe. The species is also recorded in Mongolia and Turkey.

**Ecoregion.**ER5, ER11.

**Drainage.**BS.

**BOLD ID.**CROAA006-18 (Continental part), CROAA076-18 (Continental part), CROTR219-19 (Continental part), CROTR269-19 (Continental part), CROTR397-22 (Continental part).

**Literature data.**Cerjanec et al. (2020); Ćukušić (2019); Kučinić et al. (2010, 2020b); Malicky (2009); Neu et al. (2018); Previšić et al. (2007b).

***Hydropsyche dinarica* Marinković-Gospodnetić, 1979**

**Synonym.***Hydropsyche sarnas* Oláh, 2015 (2014).

**Distribution in Croatia.** Alpine part, Mediterranean part.

**General distribution.** Alpine region, Belgium, Bosnia and Herzegovina, Croatia, Czechia, France, Germany, Greece, Iberian Peninsula, Italy, Luxembourg and borders between Albania and North Macedonia and between North Macedonia and Bulgaria.

**Ecoregion.**ER5, ER11.

**Drainage.**AS, BS.

**BOLD ID.** No DNA barcoded specimens from Croatia.

**Literature data.**Ćukušić (2019); Malicky (2014a); Neu et al. (2018); Previšić and Popijač (2010); Vučković et al. (2011, 2021).

***Hydropsyche fulvipes* (Curtis, 1834)**

**Synonym.***Hydropsyche atomaria* Kolenati, 1858.

**Distribution in Croatia.** Continental part, Mediterranean part.

**General distribution.** Alpine region, Balkan Peninsula, Belgium, Croatia, Czechia, France, Great Britain, Hungary, Luxembourg, Romania, Serbia, Slovakia, Spain, Ukraine.

**Ecoregion.**ER5, ER11.

**Drainage.**AS, BS.

**BOLD ID.**CROTR163-19 (Mediterranean part).

**Literature data.**Cerjanec et al. (2020); Ćukušić (2019); Klapálek (1906); Kučinić et al. (2021); Neu et al. (2018); Radovanović (1935).

***Hydropsyche guttata* Pictet, 1834**

**Synonyms.***Hydropsyche adspersula* Kolenati, 1858; *Hydropsyche atomaria* Stephens, 1836 (without var. obliquus); *Hydropsyche danubii* Brauer, 1857; *Hydropsyche laeta* Imhoff & Labram, 1838.

**Distribution in Croatia.** Continental part.

**General distribution.** Alpine region, Croatia, Czechia, Hungary, Slovakia.

**Ecoregion.**ER11.

**Drainage.**BS.

**BOLD ID.** No DNA barcoded specimens from Croatia.

**Literature data.**Malicky (2009); Neu et al. (2018).

***Hydropsyche incognita* Pitsch, 1993**

**Distribution in Croatia.** Continental part, Alpine part, Mediterranean part.

**General distribution.** Alpine region, Balkan Peninsula, Belgium, Croatia, Czechia, France, Germany, Hungary, Iberian Peninsula, Italy, Luxembourg, Poland, Slovakia, Ukraine. The species is also recorded in Lebanon, Syria, and Turkey.

**Ecoregion.**ER5, ER11.

**Drainage.**AS, BS.

**BOLD ID.**CROAA123-18 (Continental part), CROTR036-19 (Continental part), CROTR119-19 (Continental part).

**Literature data.**Cerjanec et al. (2020); Ćukušić (2019); Graf et al. (2008); Ivković et al. (2023); Kučinić et al. (2010, 2011, 2017b, 2020b); Neu et al. (2018); Previšić et al. (2010, 2012); Ridl et al. (2017); Vučković et al. (2011, 2021).

***Hydropsyche instabilis* (Curtis, 1834)**

**Synonyms.**Hydropsyche atomaria var. obliquus Stephens, 1836; *Hydropsyche cinerea* Pictet, 1834; *Hydropsyche hibera* Schmid, 1952; *Hydropsyche lanceolata* McLachlan, 1865; *Phryganea maculata* Donovan, 1813; *Hydropsyche pallida* Pictet, 1865; *Hydropsyche stictica* Pictet, 1865; *Hydropsyche tismanae* Murgoci, 1968.

**Distribution in Croatia.** Continental part, Alpine part, Mediterranean part.

**General distribution.** Alpine region, Apennine Peninsula with Sardinia, Balkan Peninsula, Belgium, British Islands, Croatia, Czechia, France, Germany, Iberian Peninsula, Luxembourg, Poland, Slovakia, Ukraine. The species is also recorded in Iran and Turkey.

**Ecoregion.**ER5, ER11.

**Drainage.**AS, BS.

**BOLD ID.**CROAA053-18 (Continental part), CROTR091-19 (Mediterranean part) CROTR142-19 (Alpine part), CROTR151-19 (Mediterranean part), CROTR162-19 (Mediterranean part), CROTR201-19 (Alpine part), CROTR211-19 (Continental part), CROTR238-19 (Mediterranean part), CROTR242-19 (Mediterranean part), CROTR270-19 (Alpine part), CROTR271-19 (Continental part), CROTR388-22 (Continental part), CROTR389-22 (Continental part).

**Literature data.**Cerjanec et al. (2020); Ćukušić (2019); Ćukušić et al. (2017); Ivković et al. (2013, 2023); Kučinić et al. (2011, 2017b, 2019b, 2020a, 2021); Malicky (2014a); Neu et al. (2018); Previšić et al. (2007a, 2010, 2013c); Ridl et al. (2017); Šemnički et al. (2011, 2012); Vučković et al. (2021); Žalac and Kučinić (2022).

***Hydropsyche modesta* Navas, 1925**

**Synonyms.***Hydropsyche batavorum* Botosaneanu, 1979; *Hydropsyche dissimulata* Kumanski & Botosaneanu, 1974.

**Distribution in Croatia.** Continental part.

**General distribution.** Alpine region, Apennine Peninsula with Sicily, Balkan Peninsula, Croatia, Czechia, France, Hungary, Poland, Slovakia, Ukraine. The species is also recorded in Lebanon, Syria, and Turkey.

**Ecoregion.**ER11.

**Drainage.**BS.

**BOLD ID.**CROAA046-18 (Continental part).

**Literature data.**Ćukušić (2019); Kučinić et al. (2010, 2020b); Malicky (2009); Neu et al. (2018).

***Hydropsyche mostarensis* Klapálek, 1898**

**Distribution in Croatia.** Mediterranean part.

**General distribution.** Albania, Bosnia and Herzegovina, Bulgaria, Croatia, Greece, Montenegro, North Macedonia, and Serbia.

**Ecoregion.**ER5.

**Drainage.**AS.

**BOLD ID.**CROTR086-19 (Mediterranean part).

**Literature data.**Ćukušić (2019); Kučinić et al. (2011, 2019b).

***Hydropsyche ornatula* McLachlan, 1878**

**Synonyms.***Hydropsyche atomaria* Pictet, 1834; *Hydropsyche subguttata* Martynov, 1927.

**Distribution in Croatia.** Continental part.

**General distribution.** Austria, Bulgaria, Croatia, France, Hungary, Italy (Alps and southern Italy), Poland, Romania, Serbia, Thrace, Ukraine, including European part of Russia. The species is also recorded in China.

**Ecoregion.**ER11.

**Drainage.**BS.

**BOLD ID.** No DNA barcoded specimens from Croatia.

**Literature data.**Neu et al. (2018).

***Hydropsyche pellucidula* (Curtis, 1834)**

**Synonyms.***Hydropsyche atomaria* Rambur, 1842; *Hydropsyche hibernica* Curtis, 1836; *Hydropsyche laeta* Pictet, 1834; *Philopotamus lanceolatus* Curtis, 1834; *Hydropsyche maxima* Brauer, 1857; *Hydropsyche tenuicornis* Pictet, 1834; *Hydropsyche uentchensis* Schmid, 1968; *Hydropsyche variabilis* Pictet, 1834; *Hydropsyche versicolor* Brauer, 1857.

**Distribution in Croatia.** Continental part, Alpine part, Mediterranean part.

**General distribution.** Widely distributed in Europe. The species is also recorded in China and Mongolia.

**Ecoregion.**ER5, ER11.

**Drainage.**AS, BS.

**BOLD ID.**CROAA052-18 (Alpine part), CROAA063-18 (Mediterranean part), CROTR155-19 (Mediterranean part), CROTR194-19 (Mediterranean part).

**Literature data.**Ćukušić (2019); Klapálek (1906); Kučinić et al. (2019c); Malicky (2009); Neu et al. (2018); Radovanović (1935); Vučković et al. (2021).

***Hydropsyche saxonica* McLachlan, 1884**

**Synonyms.***Hydropsyche bujori* Murgoci, 1960; *Hydropsyche dentata* Kumanski, 1974.

**Distribution in Croatia.** Continental part, Alpine part, Mediterranean part.

**General distribution.** Alpine region, Balkan Peninsula, Benelux, Bulgaria, Croatia, Czechia, Denmark, Finland, France, Germany, Great Britain, Hungary, Lithuania, Poland, Romania, Serbia, Slovakia, Sweden, Ukraine.

**Ecoregion.**ER5, ER11.

**Drainage.**AS, BS.

**BOLD ID.**CROAA070-18 (Mediterranean part), CROTR149-19 (Mediterranean part), CROTR202-19 (Mediterranean part), CROTR229-19 (Alpine part).

**Literature data.**Ćukušić (2019); Ivković et al. (2013, 2023); Kučinić et al. (2010, 2011, 2017b, 2020a, 2020b); Malicky (2014a); Neu et al. (2018); Previšić et al. (2007a, 2010); Ridl et al. (2017); Šemnički et al. (2011, 2012); Žalac and Kučinić (2022).

#### ﻿﻿Family Philopotamidae Stephens, 1829

**Genus *Philopotamus* Stephens, 1829**

***Philopotamus ludificatus* McLachlan, 1878**

**Distribution in Croatia.** Continental part, Alpine part.

**General distribution.** Alpine region, Apennine Peninsula, Belgium, Bosnia and Herzegovina, Croatia, Czechia, France, Germany, Hungary, Luxembourg, Slovakia, Ukraine.

**Ecoregion.**ER5.

**Drainage.**BS.

**BOLD ID.**CROTR329-21 (Alpine part).

**Literature data.**Neu et al. (2018).

***Philopotamus montanus* (Donovan, 1813)**

**Synonyms.***Phryganea charpentieri* Zetterstedt, 1840; *Philopotamus scopulorum* Stephens, 1836; *Philopotamus tigrinus* Brauer, 1857.

**Distribution in Croatia.** Continental part, Alpine part.

**General distribution.** Alpine region, Apennine Peninsula, Balkan Peninsula, British Islands, Croatia, Czechia, France, Germany, Hungary, Iberian Peninsula, Poland, Scandinavian Peninsula, Slovakia, Ukraine, also including Turkey. The distribution of the species requires revision due to the presence of subspecies.

**Ecoregion.**ER5, ER11.

**Drainage.**BS.

**BOLD ID.**CROAA080-18 (Continental part), CROAA130-18 (Continental part), CROTR398-22 (Continental part).

**Literature data.**Cerjanec et al. (2020); Ćukušić (2019); Ivković et al. (2023); Klapálek (1906); Kučinić et al. (2017b, 2020a); Neu et al. (2018); Previšić and Popijač (2010); Previšić et al. (2010, 2013c).

***Philopotamus variegatus* (Scopoli, 1763)**

**Synonyms.***Philopotamus pedemontanus* Navas, 1934; *Philopotamus variegatus* (Scopoli).

**Distribution in Croatia.** Continental part, Alpine part.

**General distribution.** Alpine region, Balkan Peninsula, Belgium, Croatia, Czechia, France with Corsica, Germany, Hungary, Iberian Peninsula, Italy, Luxembourg, Poland, Slovakia, Ukraine. The species is also recorded in Turkey.

On the Iberian Peninsula two subspecies are recognised, *Philopotamus varieagatus amphilectus* McLachlan, 1884 in Portugal and *Philopotamus varieagatus hispanicus* McLachlan, 1878 in Spain. Also, *Philopotamus varieagatus flavidus* Hagen, 1864 is recognised in Corsica. The distribution of the species requires revision due to the presence of subspecies.

**Ecoregion.**ER11.

**Drainage.**BS.

**BOLD ID.**CROAA121-18 (Continental part).

**Literature data.**Ćukušić (2019); Ivković et al. (2013, 2023); Kučinić et al. (2017b); Malicky (2009, 2014a); Neu et al. (2018); Previšić et al. (2010, 2013c); Šemnički et al. (2011, 2012).

#### ﻿Genus *Wormaldia* McLachlan, 1865

***Wormaldia copiosa* (McLachlan, 1868)**

**Synonyms.***Hydropsyche columbina* Pictet, 1834; *Dolophilus cuprosus* Obenberger, 1952.

**Distribution in Croatia.** Continental part, Alpine part.

**General distribution.** Alpine region, Apennine Peninsula, Croatia, Czechia, Slovakia, and Poland.

**Ecoregion.**ER5, ER11.

**Drainage.**BS.

**BOLD ID.**CROTR195-19 (Alpine part), CROTR384-22 (Continental part), CROTR385-22 (Continental part).

**Literature data.**Cerjanec et al. (2020); Ćukušić (2019); Kučinić et al. (2020a); Neu et al. (2018); Previšić and Popijač (2010).

***Wormaldia pulla* (McLachlan, 1878)**

**Distribution in Croatia.** Continental part.

**General distribution.** Alpine region, Apennine Peninsula, Balkan Peninsula, Czechia, Germany, Poland, Slovakia, Ukraine.

**Ecoregion.**ER11.

**Drainage.**BS.

**BOLD ID.**CROAA45-18 (Continental part).

**Literature data.**Ćukušić (2019); Kučinić et al. (2015); Previšić et al. (2013c).

***Wormaldia subnigra* McLachlan, 1865**

**Synonyms.***Wormaldia triangulifera thasica* Malicky, 1983; *Wormaldia asterusia* Malicky, 1972; *Wormaldia subnigra* McLachlan, 1865.

**Distribution in Croatia.** Continental part, Alpine part, Mediterranean part.

**General distribution.** Widely distributed in Europe. The species is also recorded in Lebanon, Morocco, and Turkey.

**Ecoregion.**ER5, ER11.

**Drainage.**AS, BS.

**BOLD ID.**CROAA019-18 (Continental part), CROAA099-18 (Mediterranean part), CROTR099-19 (Mediterranean part), CROTR246-19 (Alpine part).

**Literature data.**Cerjanec et al. (2020); Ćukušić (2019); Ćukušić et al. (2017); Gottstein Matočec et al. (2002); Graf et al. (2008); Ivković et al. (2023); Klapálek (1906); Kučinić et al. (2008, 2010, 2011, 2017b, 2020a, 2020b); Langhoffer (1912); Malicky (2014a); Oláh (2010); Neu et al. (2018); Previšić et al. (2010, 2007a, 2012); Radovanović (1935); Ridl et al. (2017); Šemnički et al. (2011, 2012); Vučković et al. (2021); Žalac and Kučinić (2022).

***Wormaldia subterranea* Radovanović, 1932**

**Distribution in Croatia.** Continental part, Alpine part, Mediterranean part.

**General distribution.** Alpine region, Bosnia and Herzegovina, Croatia, France, Germany, Hungary, Luxembourg, Montenegro, Poland, Romania, Slovakia, Ukraine. Transitional form of *Wormaldia occipitalis* (Pictet, 1834) and *W. subterranea* are recognised in Italy (Alps).

**Ecoregion.**ER5, ER11.

**Drainage.**AS, BS.

**BOLD ID.**CROAA044-18 (Continental part), CROTR061-19 (Continental part), CROTR068-19 (Alpine part), CROTR245-19 (Continental part), CROTR382-22 (Continental part), CROTR383-22 (Continental part), CROTR387-22 (Continental part), CROTR390-22 (Continental part), CROTR393-22 (Continental part), CROTR395-22 (Continental part).

**Literature data.**Cerjanec et al. (2020); Ćukušić (2019); Gottstein Matočec et al. (2002); Ivković et al. (2023); Kučinić et al. (2010, 2017b, 2020a, 2020b); Malicky (2009, 2014a); Neu et al. (2018); Previšić et al. (2010, 2012, 2013c); Šemnički et al. (2011, 2012); Vučković et al. (2021); Žalac and Kučinić (2022).

#### ﻿﻿Family Polycentropodidae Ulmer, 1903

**Genus *Cyrnus* Stephens, 1836**

***Cyrnus crenaticornis* (Kolenati, 1859)**

**Distribution in Croatia.** Continental part.

**General distribution.** Alpine region, Belarus, Benelux, Croatia, Czechia, Denmark, Estonia, Finland, France, Germany, Hungary, Latvia, Lithuania, Montenegro, Poland, Romania, Slovakia, Sweeden, Ukraine, including also European part of Russia.

**Ecoregion.**ER11.

**Drainage.**BS.

**BOLD ID.**CROTR362-21 (Continental part).

**Literature data.**Ćuk et al. (2021); Neu et al. (2018).

***Cyrnus trimaculatus* (Curtis, 1834)**

**Synonyms.***Plectrocnemia atomaria* Kolenati, 1859; *Philopotamus flavomaculatus* Rambur, 1842; *Cyrnus pulchellus* Stephens, 1836; *Cyrnus unipunctatus* Stephens, 1836.

**Distribution in Croatia.** Continental part, Alpine part, Mediterranean part.

**General distribution.** Widely distributed in Europe, including European part of Russia. The species is also recorded in Turkey.

**Ecoregion.**ER5, ER11.

**Drainage.**AS, BS.

**BOLD ID.**CROAA118-18 (Alpine), CROAA129-18 (Alpine part), CROTR075-19 (Alpine part), CROTR160-19 (Mediterranean part), CROTR161-19 (Mediterranean part), CROTR217-19 (Mediterranean part).

**Literature data.**Cerjanec et al. (2020); Ćukušić (2019); Ćukušić et al. (2017); Graf et al. (2008); Ivković et al. (2023); Kučinić et al. (2008, 2010, 2011, 2017b, 2019b, 2020a, 2020b); Malicky (2014a); Neu et al. (2018); Previšić et al. (2007b, 2010); Šemnički et al. (2011, 2012); Vučković et al. (2021); Žalac and Kučinić (2022).

#### ﻿Genus *Holocentropus* McLachlan, 1878

***Holocentropus stagnalis* (Albarda, 1874)**

**Synonym.***Plectrocnemia lituratus* Kolenati, 1859.

**Distribution in Croatia.** Continental part.

**General distribution.** Austria, Belarus, Belgium, Bulgaria, Croatia, Czechia, Denmark, Estonia, France, Germany, Great Britain, Hungary, Netherlands, Poland, Serbia, Slovakia, Slovenia, Sweden, Switzerland, Ukraine.

**Ecoregion.**ER11.

**Drainage.**BS.

**BOLD ID.** No DNA barcoded specimens from Croatia.

**Literature data.**Malicky (2009); Neu et al. (2018).

#### ﻿Genus *Neureclipsis* McLachlan, 1864

***Neureclipsis bimaculata* (Linnaeus, 1758)**

**Synonyms.***Polycentropus concolor* Burmeister, 1839; *Phryganea noctuaeformis* von Paula Schrank, 1802; Anticyra ? robusta Walker, 1852; *Phryganea tigurinensis* Fabricius, 1798; *Philopotamus variegatus* Schoch, 1884.

**Distribution in Croatia.** Continental part, Alpine part, Mediterranean part.

**General distribution.** Widely distributed in Europe, including European part of Russia. The species is also recorded in Russian Far East, Mongolia, and North America.

**Ecoregion.**ER5, ER11.

**Drainage.**AS, BS.

**BOLD ID.**CROAA071-18 (Continental part).

**Literature data.**Ćukušić (2019); Ivković et al. (2023); Kučinić et al. (2011, 2017b); Malicky (2014a); Neu et al. (2018); Previšić et al. (2007a, 2007b, 2010); Ridl et al. (2017); Šemnički et al. (2011, 2012).

#### ﻿Genus *Plectrocnemia* Stephens, 1836

***Plectrocnemia brevis* McLachlan, 1871**

**Synonym.***Plectrocnemia brevis gracilligonopoda* Botosaneanu, 1960.

**Distribution in Croatia.** Continental part, Alpine part.

**General distribution.** Alpine region, Balkan Peninsula, Belgium, Croatia, Czechia, Denmark, Germany, Great Britain, Hungary, Italy, France (also border line between Spain and France), Luxembourg, Poland, Slovakia, Ukraine. The species is also recorded in Turkey.

**Ecoregion.**ER5, ER11.

**Drainage.**BS.

**BOLD ID.**CROTR140-19 (Alpine part), CROTR392-22 (Continental part).

**Literature data.**Cerjanec et al. (2020); Ćukušić (2019); Ivković et al. (2023); Kučinić et al. (2008, 2017b, 2020a); Malicky (2014a); Neu et al. (2018); Previšić et al. (2007a, 2010, 2012).

***Plectrocnemia conspersa* (Curtis, 1834)**

**Synonyms.***Plectrocnemia atomaria* Walser, 1864; *Plectrocnemiella carelica* Nybom, 1950; *Hydropsyche senex* Pictet, 1834; *Crunophila torrentium* Kolenati, 1859.

**Distribution in Croatia.** Continental part, Alpine part, Mediterranean part.

**General distribution.** Widely distributed in Europe, including European part of Russia. The species is also recorded in Turkey. The subspecies *Plectrocnemia conspersa keftiu* Malicky, 1974 has been recorded in Crete.

**Ecoregion.**ER5, ER11.

**Drainage.**AS, BS.

**BOLD ID.**CROTR008-19 (Alpine part), CROTR076-19 (Alpine part), CROTR144-19 (Alpine), CROTR192-19 (Alpine), CROTR380-22 (Continental part).

**Literature data.**Cerjanec et al. (2020); Ćukušić (2019); Ivković et al. (2023); Kučinić et al. (2008, 2010, 2011, 2017b, 2019a, 2020a, 2020b); Malicky (2014a); Marinković-Gospodnetić (1979); Neu et al. (2018); Oláh (2010); Previšić et al. (2010, 2012, 2013c); Vučković et al. (2021); Žalac and Kučinić (2022).

***Plectrocnemia geniculata* McLachlan, 1871**

**Distribution in Croatia.** Continental part.

**General distribution.** Five subspecies are recognised. *Plectrocnemia geniculata geniculata* McLachlan, 1871 has the widest distribution, including the Alpine region, Belgium, the British Isles, Czechia, France, Germany, Greece, Hungary, Luxembourg, and central Italy.

**Ecoregion.**ER5.

**Drainage.**BS.

**BOLD ID.** No DNA barcoded specimens from Croatia.

**Literature data.**Kučinić et al. (2015).

#### ﻿Genus *Polycentropus* Curtis, 1835

***Polycentropus excisus* Klapálek, 1894**

**Distribution in Croatia.** Continental part, Alpine part, Mediterranean part.

**General distribution.** Alpine region, Balkan Peninsula, Croatia, Romania, Slovakia, Ukraine.

**Ecoregion.**ER5, ER11.

**Drainage.**AS, BS.

**BOLD ID.**CROAA110-18 (Mediterranean part), CROTR214-19 (Continental part), CROTR396-22 (Continental part).

**Literature data.**Ćukušić (2019); Ivković et al. (2023); Kučinić et al. (2017b, 2021); Malicky (2014a); Neu et al. (2018); Previšić et al. (2010); Šemnički et al. (2011, 2012); Žalac and Kučinić (2022).

***Polycentropus flavomaculatus* (Pictet, 1834)**

**Synonyms.***Phryganea atomaria* von Paula Schrank, 1802; *Hydropsyche brevicollis* Pictet, 1834; *Polycentropus concinnus* Stephens, 1836; *Polycentropus fuliginosus* Stephens, 1836; *Plectrocnemia irrorata* Brauer, 1857; *Polycentropus multiguttatus* Curtis, 1835; *Polycentropus pyrrhoceras* Stephens, 1836; *Polycentropus subpunctatus* Stephens, 1836; *Polycentropus trimaculatus* Stephens, 1836; *Hydropsyche vitreus* Pictet, 1834.

**Distribution in Croatia.** Continental part, Alpine part, Mediterranean part.

**General distribution.** Widely distributed in Europe, including European part of Russia. The species is also recorded in Turkey.

**Ecoregion.**ER5, ER11.

**Drainage.**AS, BS.

**BOLD ID.**CROTR190-19 (Alpine part), CROTR272-19 (Mediterranean part), CROTR273-19 (Mediterranean part).

**Literature data.**Ćukušić (2019); Graf et al. (2008); Ivković et al. (2013, 2023); Kučinić et al. (2008, 2010, 2011, 2017b, 2019a, 2020a, 2020b); Malicky (2014a); Neu et al. (2018); Previšić et al. (2007a, 2010); Šemnički et al. (2011); Vučković et al. (2021).

***Polycentropus ierapetra slovenica* Malicky, 1998**

**Distribution in Croatia.** Alpine part, Mediterranean part.

**General distribution.** Croatia, Italy, Slovenia (Alpine region) and border between Albania and Kosovo.

**Ecoregion.**ER5.

**Drainage.**AS, BS.

**BOLD ID.** No DNA barcoded specimens from Croatia.

**Literature data.**Neu et al. (2018); Vučković et al. (2011).

***Polycentropus irroratus* (Curtis, 1835)**

**Distribution in Croatia.** Continental part, Alpine part, Mediterranean part.

**General distribution.** Widely distributed in Europe.

**Ecoregion.**ER5, ER11.

**Drainage.**AS, BS.

**BOLD ID.**CROAA010-18 (Continental part), CROAA088-18 (Mediterranean part), CROTR046-19 (Continental part).

**Literature data.**Cerjanec et al. (2020); Ćukušić (2019); Ćukušić et al. (2017); Kučinić et al. (2011, 2020a); Malicky (2014a); Neu et al. (2018); Ridl et al. (2017).

***Polycentropus schmidi* Novak & Botosaneanu, 1965**

**Distribution in Croatia.** Continental part, Alpine part, Mediterranean part.

**General distribution.** Alpine region, Croatia, Czechia, France, Hungary, Poland, Romania, Ukraine.

**Ecoregion.**ER5, ER11.

**Drainage.**AS, BS.

**BOLD ID.**CROAA017-18 (Continental part).

**Literature data.**Ćukušić (2019); Ivković et al. (2023); Kučinić et al. (2017b, 2020b); Malicky (2014a); Neu et al. (2018); Previšić et al. (2010); Šemnički et al. (2011, 2012); Vučković et al. (2021).

#### ﻿﻿Family Psychomyiidae Walker, 1852

**Genu *s Lype* McLachlan, 1878**

***Lype phaeopa* (Stephens, 1836)**

**Synonyms.***Psychomia derelicta* McLachlan, 1863; *Anticyra gracilipes* Stephens, 1836; *Beraea melas* Brauer, 1857; *Lype phaeopa meridionalis* Moretti, 1986 (1984); Tinodes (Homoeocerus) pusillus Kolenati, 1859; *Lype sinuata* McLachlan, 1878; *Cyrnus urbanus* Stephens, 1836.

**Distribution in Croatia.** Continental part, Alpine part, Mediterranean part.

**General distribution.** Widely distributed in Europe, including European part of Russia. The species is also recorded in Turkey and Iran.

**Ecoregion.**ER5.

**Drainage.**AS, BS.

**BOLD ID.** No DNA barcoded specimens from Croatia.

**Literature data.**Cerjanec et al. (2020); Ćukušić (2019); Ivković et al. (2023); Kučinić et al. (2011, 2017b); Malicky (2014a); Neu et al. (2018); Previšić et al. (2010); Ridl et al. (2017); Šemnički et al. (2011, 2012).

***Lype reducta* (Hagen, 1868)**

**Synonym.***Lype flavospinosa* Mosely, 1930.

**Distribution in Croatia.** Continental part, Alpine part, Mediterranean part.

**General distribution.** Widely distributed in Europe, including European part of Russia. The species is also recorded in Lebanon and Turkey.

**Ecoregion.**ER5, ER11.

**Drainage.**AS, BS.

**BOLD ID.**CROAA014-18 (Mediterranean part), CROTR081-19 (Mediterranean part), CROTR223-19 (Alpine part).

**Literature data.**Cerjanec et al. (2020); Ćukušić (2019); Ćukušić et al. (2017); Graf et al. (2008); Ivković et al. (2013, 2023); Kučinić et al. (2011, 2017b, 2020a); Malicky (2014a); Neu et al. (2018); Previšić et al. (2007a; 2010, 2013c); Ridl et al. (2017); Šemnički et al. (2011, 2012); Vučković et al. (2021); Žalac and Kučinić (2022).

#### ﻿Genus *Psychomyia* Latreille, 1829

***Psychomyia klapaleki* Malicky, 1995**

**Distribution in Croatia.** Alpine part, Mediterranean part.

**General distribution.** Slovenia, Balkan Peninsula.

**Ecoregion.**ER5.

**Drainage.**AS, BS.

**BOLD ID.**CROAA038-18 (Alpine part), CROAA112-18 (Alpine part).

**Literature data.**Cerjanec et al. (2020); Ćukušić (2019); Ćukušić et al. (2017); Graf et al. (2008); Ivković et al. (2023); Kučinić et al. (2017b, 2019a, 2020a); Malicky (2014a); Neu et al. (2018); Previšić and Popijač (2010); Previšić et al. (2010); Vučković et al. (2021).

***Psychomyia pusilla* (Fabricius, 1781)**

**Synonyms.***Psychomyia acuta* Pictet, 1834; *Psychomyia annulicornis* Pictet, 1834; *Psychomyia bifurcata* Mosely, 1938; *Anticyra ciliaris* Stephens, 1836; *Anticyra gracilipes* Curtis, 1834; *Anticyra latipes* Curtis, 1834; *Psychomyia shelkovnikovi* Martynov, 1925; *Anticyra subochracea* Stephens, 1836; *Psychomyia tenuis* Pictet, 1834; *Cyrnus unicolor* Stephens, 1836.

**Distribution in Croatia.** Continental part, Alpine part, Mediterranean part.

**General distribution.** Widely distributed in Europe, including European part of Russia. The species is also recorded in Iran, Israel, Lebanon, Siberia, Syria, and Turkey.

**Ecoregion.**ER5, ER11.

**Drainage.**AS, BS.

**BOLD ID.**CROAA029-18 (Continental part), CROTR133-19 (Continental part), CROTR177-19 (Continental part), CROTR178-19 (Mediterranean part), CROTR179-19 (Continental part), CROTR180-19 (Alpine part), CROTR228-19 (Alpine part), CROTR274-19 (Continental part).

**Literature data.**Cerjanec et al. (2020); Ćukušić (2019); Klapálek (1906); Kučinić et al. (2010, 2020b); Malicky (2009); Neu et al. (2018); Previšić et al. (2007b).

#### ﻿Genus *Tinodes* Curtis, 1834

***Tinodes andrasi* Oláh, 2010**

**Distribution in Croatia.** Mediterranean part.

**General distribution.** An endemic species of the Dinarides, with the type locality in the upper course of the Ljuta River (Konavle region). The species has also been recorded at a single locality in Montenegro (Kučinić et al. 2021; Malicky, 2018).

**Ecoregion.**ER5.

**Drainage.**AS.

**BOLD ID.**CROTR352-21 (Mediterranean part), CROTR353-21 (Mediterranean part).

**Literature data.**Kučinić et al. (2016, 2021); Malicky (2018); Neu et al. (2018); Oláh (2010).

***Tinodes antonioi* Botosaneanu & Taticchi-Viganò, 1974**

**Distribution in Croatia.** Mediterranean part.

**General distribution.** Apennine Peninsula.

**Ecoregion.**ER5.

**Drainage.**AS.

**BOLD ID.**NIP002-16 (Mediterranean part), NIP003-16 (Mediterranean part), NIP004-16 (Mediterranean part).

**Literature data.**Ćukušić (2019); Kučinić et al. (2016, 2020a).

***Tinodes braueri* McLachlan, 1878**

**Distribution in Croatia.** Alpine part, Mediterranean part.

**General distribution.** Balkan Peninsula, south Europe.

**Ecoregion.**ER5.

**Drainage.**AS, BS.

**BOLD ID.**CROTR125-19 (Mediterranean part), CROTR354-21 (Mediterranean part), NIP005-16 (Mediterranean part).

**Literature data.**Cerjanec et al. (2020); Ćukušić (2019); Graf et al. (2008); Kučinić et al. (2011, 2016, 2019b, 2021); Malicky (2014a); Neu et al. (2018); Previšić et al. (2012); Ridl et al. (2017); Vučković et al. (2021); Žalac and Kučinić (2022).

***Tinodes dives jeekeli* Botosaneanu, 1980**

**Distribution in Croatia.** Continental part, Alpine part, Mediterranean part.

**General distribution.** Central Europe.

**Ecoregion.**ER5, ER11.

**Drainage.**AS, BS.

**BOLD ID.**NIP007-16 (Alpine part).

**Literature data.**Botosaneanu (1980); Cerjanec et al. (2020); Ćukušić (2019); Ivković et al. (2013, 2023); Kučinić et al. (2008, 2011, 2016, 2017b, 2020a); Malicky (2014a); Marinković-Gospodnetić (1979); Neu et al. (2018); Oláh et al. (2021); Previšić and Popijač (2010); Previšić et al. (2007a, 2010, 2014a); Šemnički et al. (2011, 2012); Vučković et al. (2011, 2021).

***Tinodes pallidulus* McLachlan, 1878**

**Distribution in Croatia.** Continental part, Mediterranean part.

**General distribution.** Alpine region, Balkan Peninsula, Belgium, Croatia, Czechia, Denmark, France, Germany, Gotland (Island of Sweeden), Great Britain, Hungary, Luxembourg, Slovakia. The species is also recorded in Turkey.

**Ecoregion.**ER5, ER11.

**Drainage.**AS, BS.

**BOLD ID.**CROTR158-19 (Mediterranean part), CROTR203-19 (Mediterranean part), CROTR350-21 (Mediterranean part), NIP006-16 (Mediterranean part).

**Literature data.**Ćukušić (2019); Kučinić et al. (2016, 2019b, 2020a, 2021); Malicky (2014a); Neu et al. (2018); Previšić et al. (2007b).

***Tinodes rostocki* McLachlan, 1878**

**Distribution in Croatia.** Continental part, Alpine part.

**General distribution.** Alpine region, Balkan Peninsula, Belgium, Croatia, Czechia, France, Germany, Great Britain, Hungary, Luxembourg, Poland, Slovakia, Spain, Ukraine.

**Ecoregion.**ER5, ER11.

**Drainage.**BS.

**BOLD ID.**CROTR148-19 (Continental part).

**Literature data.**Cerjanec et al. (2020); Ćukušić (2019); Kučinić et al. (2016); Malicky (2018); Neu et al. (2018); Previšić and Popijač (2010); Previšić et al. (2013c).

***Tinodes unicolor* (Pictet, 1834)**

**Synonym.***Hydropsyche urbana* Pictet, 1834.

**Distribution in Croatia.** Continental part, Alpine part, Mediterranean part.

**General distribution.** Alpine region, Balkan Peninsula, Belgium, British Islands, Croatia, Czechia, Denmark, France, Germany, Hungary, Iberian Peninsula, Luxembourg, Slovakia.

**Ecoregion.**ER5, ER11.

**Drainage.**AS, BS.

**BOLD ID.**CROAA120-18 (Continental part), CROTR082-19 (Mediterranean part), CROTR089-19 (Mediterranean part), CROTR204-19 (Continental part), CROTR205-19 (Mediterranean part), CROTR206-19 (Mediterranean part), CROTR386-22 (Continental part).

**Literature data.**Ćukušić (2019); Ivković et al. (2023); Kučinić et al. (2016, 2017b, 2020a); Malicky (2009, 2014a); Neu et al. (2018); Previšić et al. (2007a, 2010); Ridl et al. (2017); Šemnički et al. (2011, 2012); Žalac and Kučinić (2022).

***Tinodes waeneri* (Linnaeus, 1758)**

**Synonyms.***Tinodes annulicornis* Stephens, 1836; *Tinodes flaviceps* Stephens, 1836; *Tinodes flavipes* Hagen, 1860; *Phryganea griseola* Zetterstedt, 1840; *Philopotamus longipennis* Rambur, 1842; *Tinodes luridus* Curtis, 1834; *Hydropsyche microcephala* Pictet, 1834; *Tinodes pallescens* Stephens, 1836; *Tinodes pallipes* Stephens, 1836; *Tinodes xanthoceras* Stephens, 1836.

**Distribution in Croatia.** Alpine part, Mediterranean part.

**General distribution.** Alpine region, Apennine Peninsula including Sardinia and Sicily, Benelux, Bosnia and Herzegovina, British Islands, Croatia, Czechia, Estonia, France, Germany, Greece, Hungary, Iberian Peninsula, Lithuania, Poland, Scandinavian Peninsula, Slovakia. The species is also registered in Morocco.

**Ecoregion.**ER5.

**Drainage.**AS, BS.

**BOLD ID.**CROTR034-19 (Mediterranean part), CROTR159-19 (Mediterranean part), CROTR164-19 (Alpine part), CROTR176-19 (Mediterranean part), NIP001-16 (Mediterranean part).

**Literature data.**Ćukušić (2019); Ivković et al. (2023); Kučinić et al. (2008, 2011, 2016, 2017b, 2019b, 2019c, 2020a); Malicky (2014a); Neu et al. (2018); Previšić et al. (2010); Ridl et al. (2017); Šemnički et al. (2011, 2012); Vučković et al. (2021).

#### ﻿﻿Suborder Integripalpia Martynov, 1924

﻿**Family Apataniidae Wallengren, 1886**

**Genus *Apatania* Kolenati, 1848**

***Apatania muliebris* McLachlan, 1866**

**Distribution in Croatia.** Alpine part.

**General distribution.** Alpine region (excluding Italy and Slovenia), Belgium, British Islands, Croatia, Denmark, France, Germany, Hungary, Montenegro, Netherlands, Norway, Sweden.

**Ecoregion.**ER5.

**Drainage.**BS.

**BOLD ID.** No DNA barcoded specimens from Croatia.

**Literature data.**Ivković et al. (2023); Kučinić et al. (2017b); Neu et al. (2018); Previšić et al. (2013b).

#### ﻿﻿Family Beraeidae Wallengren, 1891

**Genus *Beraea* Stephens, 1836**

***Beraea dira* McLachlan, 1875**

**Distribution in Croatia.** Alpine part, Mediterranean part.

**General distribution.** Alpine region (Italy and Slovenia).

**Ecoregion.**ER5.

**Drainage.**AS, BS.

**BOLD ID.** No DNA barcoded specimens from Croatia.

**Literature data.**Ćuk et al. (2015); Ćukušić (2019); Neu et al. (2018).

***Beraea maura* (Curtis, 1834)**

**Distribution in Croatia.** Continental part.

**General distribution.** Alpine region, Apennine Peninsula, Balkan Peninsula, Benelux, British Islands, Czechia, Denmark, Estonia, France, Germany, Hungary, Poland, Slovakia, Sweden, Ukraine.

**Ecoregion.**ER11.

**Drainage.**BS.

**BOLD ID.** No DNA barcoded specimens from Croatia.

**Literature data.**Malicky (2009); Previšić et al. (2013c).

***Beraea pullata* (Curtis, 1834)**

**Synonyms.***Beraea albipes* Stephens, 1836; *Beraea alticola* Vaillant, 1967; *Nais aterrima* Brauer, 1857; *Rhyacophila barbata* Pictet, 1834; *Beraea marshamella* Stephens, 1836; *Rhyacophila melas* Pictet, 1834; *Phryganea minuta* Zetterstedt, 1840; *Rhyacophila nigrocincta* Pictet, 1834; *Rhyacophila penicillus* Pictet, 1834; *Phryganea pygmaea* Fabricius, 1798.

**Distribution in Croatia.** Continental part, Alpine part.

**General distribution.** Widely distributed in Europe, including European part of Russia (along the Ural Mountains).

**Ecoregion.**ER5.

**Drainage.**AS, BS.

**BOLD ID.**CROTR080-19 (Alpine part).

**Literature data.**Ćukušić (2019); Ivković et al. (2023); Klapálek (1906); Kučinić et al. (2017b, 2019a, 2020a); Malicky (2014a); Marinković-Gospodnetić (1979); Neu et al. (2018); Previšić et al. (2010); Žalac and Kučinić (2022).

#### ﻿Genus *Beraeamyia* Mosely, 1930

***Beraeamyia schmidi* Botosaneanu, 1960**

**Distribution in Croatia.** Alpine part, Mediterranean part.

**General distribution.** Albania, Bosnia and Herzegovina, Croatia, Greece, Montenegro, Serbia (should be verified), Slovenia.

**Ecoregion.**ER5.

**Drainage.**AS, BS.

**BOLD ID.**CROAA117-18 (Alpine part).

**Literature data.**Ćukušić (2019); Ivković et al. (2013, 2023); Kučinić et al. (2017b); Malicky (2005a, 2014a); Neu et al. (2018); Previšić et al. (2010); Šemnički et al. (2011, 2012); Vučković et al. (2021).

#### ﻿Genus *Beraeodes* Eaton, 1867

***Beraeodes minutus* (Linnaeus, 1761)**

**Synonym.***Beraeamyia eideli* Doehler, 1981.

**Distribution in Croatia.** Alpine part.

**General distribution.** Widely distributed in Europe, including European part of Russia. The species is also recorded in Turkey.

**Ecoregion.**ER5.

**Drainage.**BS.

**BOLD ID.** No DNA barcoded specimens from Croatia.

**Literature data.**Ivković et al. (2023); Kučinić et al. (2017b); Malicky (2014a); Neu et al. (2018); Šemnički et al. (2011, 2012).

#### ﻿Genus *Ernodes* Wallengren, 1891

***Ernodes articularis* (Pictet, 1834)**

**Synonym.***Beraea martynovi* Murgoci & Juncu, 1955.

**Distribution in Croatia.** Continental part, Alpine part, Mediterranean part.

**General distribution.** Widely distributed in Europe. The species is also recorded in Turkey.

**Ecoregion.**ER5, ER11.

**Drainage.**AS, BS.

**BOLD ID.**CROAA074-18 (Mediterranean part), CROAA105-18 (Alpine part).

**Literature data.**Ćukušić (2019); Ivković et al. (2023); Kučinić et al. (2017b); Malicky (2014a); Neu et al. (2018); Previšić et al. (2010, 2013c).

***Ernodes vicinus* (McLachlan, 1879)**

**Distribution in Croatia.** Continental part, Alpine part, Mediterranean part.

**General distribution.** Alpine region, Bosnia and Herzegovina, Croatia, Czechia, France, Germany, Italy, Poland, Romania, Slovakia, Ukraine.

**Ecoregion.**ER5.

**Drainage.**AS, BS.

**BOLD ID.** No DNA barcoded specimens from Croatia.

**Literature data.**Ćukušić (2019); Ivković et al. (2023); Kučinić et al. (2017b); Malicky (2014a); Neu et al. (2018); Previšić et al. (2010, 2013a); Šemnički et al. (2011, 2012); Žalac and Kučinić (2022).

#### ﻿﻿Family Brachycentridae Ulmer, 1903

**Genus *Brachycentrus* Curtis, 1834**

***Brachycentrus montanus* Klapálek, 1892**

**Synonyms.***Brachycentrus carpathicus* Dziedzielewicz, 1895; *Silo sexguttata* von Heyden, 1896.

**Distribution in Croatia.** Alpine part.

**General distribution.** Alpine region, Balkan Peninsula, Belgium, Czechia, France, Germany, Italy, Luxembourg, Slovakia, Ukraine.

**Ecoregion.**ER5.

**Drainage.**BS.

**BOLD ID.**CROAA057-18 (Alpine part), CROAA102-18 (Alpine part).

**Literature data.**Ćukušić (2019); Neu et al. (2018); Previšić and Popijač (2010).

***Brachycentrus subnubilus* Curtis, 1834**

**Synonyms.***Brachycentrus caucasicus* Martynov, 1926; *Brachycentrus concolor* Stephens, 1836; *Brachycentrus costalis* Stephens, 1836; *Brachycentrus maracandicus* McLachlan, 1875; *Hydronautia nubila* Kolenati, 1859; *Pogonostoma vernum* Rambur, 1842.

**Distribution in Croatia.** Continental part, Alpine part.

**General distribution.** Widely distributed in Europe, including European part of Russia. The species is also recorded in Russian Far East and Mongolia.

**Ecoregion.**ER5, ER11.

**Drainage.**BS.

**BOLD ID.** No DNA barcoded specimens from Croatia.

**Literature data.**Ćukušić (2019); (Malicky 2014a); Neu et al. (2018); Previšić and Popijač (2010).

#### ﻿Genus *Micrasema* McLachlan, 1876

***Micrasema minimum* McLachlan, 1876**

**Synonyms.***Micrasema exiguus* McLachlan, 1876; *Beraea valirana* Navas, 1917.

**Distribution in Croatia.** Continental part, Alpine part.

**General distribution.** Alpine region, Apennine Peninsula, Balkan Peninsula, Belgium, Czechia, France, Germany, Iberian Peninsula, Luxembourg, Slovakia, Ukraine. The species is also recorded in Morocco.

**Ecoregion.**ER5.

**Drainage.**BS.

**BOLD ID.** No DNA barcoded specimens from Croatia.

**Literature data.**Cerjanec et al. (2020); Kučinić et al. (2015).

***Micrasema sericeum* Klapálek, 1902**

**Distribution in Croatia.** Alpine part.

**General distribution.** Balkan Peninsula.

**Ecoregion.**ER5.

**Drainage.**BS.

**BOLD ID.** No DNA barcoded specimens from Croatia.

**Literature data.**Cerjanec et al. (2020).

***Micrasema setiferum* (Pictet, 1834)**

**Synonyms.***Hydronautia maculata concolor* Kolenati, 1848; *Dasystoma nigrum* Brauer, 1857.

**Distribution in Croatia.** Alpine part.

**General distribution.** Alpine region, Belgium, Croatia, Czechia, Estonia, France, Germany, Hungary, Lithuania, Luxembourg, Scandinavian Peninsula, Slovakia, Ukraine.

**Ecoregion.**ER5.

**Drainage.**BS.

**BOLD ID.**CROAA039-18 (Alpine part), CROAA061-18 (Alpine part), CROAA086-18 (Alpine part).

**Literature data.**Ćukušić (2019); Neu et al. (2018); Previšić and Popijač (2010).

#### ﻿﻿Family Glossosomatidae Wallengren, 1891

**Genus *Agapetus* Curtis, 1834**

***Agapetus delicatulus* McLachlan, 1884**

**Distribution in Croatia.** Continental part, Alpine part, Mediterranean part.

**General distribution.** Austria, Balkan Peninsula, Belgium, British Islands, Czechia, France, Germany, Hungary, Iberian Peninsula, Italy, Luxembourg, Poland, Slovakia, Slovenia, Ukraine. The species is also recorded in Turkey.

**Ecoregion.**ER5.

**Drainage.**AS, BS.

**BOLD ID.** No DNA barcoded specimens from Croatia.

**Literature data.**Cerjanec et al. (2020); Kučinić et al. (2020b); Neu et al. (2018).

***Agapetus kampos* Oláh, 2013**

**Distribution in Croatia.** Mediterranean part.

**General distribution.** Croatia (Kučinić et al. 2021); Montenegro.

**Ecoregion.**ER5.

**Drainage.**AS.

**BOLD ID.**CROTR216-19 (Mediterranean part), CROTR225-19 (Mediterranean part), CROTR230-19 (Mediterranean part), CROTR347-21 (Mediterranean part), CROTR348-21 (Mediterranean part), NIP011-17 (Mediterranean part).

**Literature data.**Ćukušić (2019); Kučinić et al. (2021); Žalac and Kučinić (2022).

***Agapetus laniger* Pictet, 1834**

**Synonym.***Agapetus pactus* McLachlan, 1879.

**Distribution in Croatia.** Continental part, Alpine part.

**General distribution.** Alpine region, Apennine Peninsula, Balkan Peninsula, Benelux, Croatia, Czechia, France, Germany, Hungary, Iberian Peninsula, Poland, Slovakia, Ukraine. The species is also recorded in Turkey.

**Ecoregion.**ER5, ER11.

**Drainage.**BS.

**BOLD ID.**NIPAG001-17 (Continental part).

**Literature data.**Cerjanec et al. (2020); Ćukušić (2019); Neu et al. (2018).

***Agapetus ochripes* Curtis, 1834**

**Synonyms.***Agapetus comatus* Pictet, 1834; *Hydroptila pumilio* Zetterstedt, 1840.

**Distribution in Croatia.** Continental part, Alpine part, Mediterranean part.

**General distribution.** Widely distributed in Europe, including European part of Russia (along the Ural Mountains).

**Ecoregion.**ER5, ER11.

**Drainage.**AS, BS.

**BOLD ID.**CROTR010-19 (Continental part), CROTR199-19 (Alpine part).

**Literature data.**Cerjanec et al. (2020); Ćukušić (2019); Kučinić et al. (2011, 2019a); Malicky (2014a); Neu et al. (2018); Previšić et al. (2012, 2013c); Vučković et al. (2011); Žalac and Kučinić (2022).

#### ﻿Genus *Glossosoma* Curtis, 1834

***Glossosoma bifidum* McLachlan, 1879**

**Synonym.***Glossosoma beaumonti* Schmid, 1947.

**Distribution in Croatia.** Continental part, Alpine part, Mediterranean part.

**General distribution.** Alpine region, Balkan Peninsula.

**Ecoregion.**ER5.

**Drainage.**AS, BS.

**BOLD ID.**NIPAG004-17 (Continental part).

**Literature data.**Cerjanec et al. (2020); Ćukušić (2019); Ivković et al. (2023); Kučinić et al. (2008, 2011, 2017b); Malicky (2014a); Neu et al. (2018); Previšić et al. (2007a, 2010); Vučković et al. (2011).

***Glossosoma conforme* Neboiss, 1963**

**Synonym.***Glossosoma boltoni* McLachlan, 1879.

**Distribution in Croatia.** Continental part.

**General distribution.** Alpine region, Apennine Peninsula, Balkan Peninsula, Belgium, Czechia, France (also along the border between France and Spain), Germany, Hungary, Luxembourg, Poland, Slovakia, Ukraine.

**Ecoregion.**ER11.

**Drainage.**BS.

**BOLD ID.**CROTR014-19 (Continental part), CROTR210-19 (Continental part).

**Literature data.**Ćukušić (2019); Kučinić et al. (2015); Previšić et al. (2013c).

***Glossosoma discophorum* Klapálek, 1902**

**Distribution in Croatia.** Continental part, Alpine part, Mediterranean part.

**General distribution.** Balkan Peninsula.

**Ecoregion.**ER5.

**Drainage.**AS, BS.

**BOLD ID.**CROAA004-18 (Alpine part), CROAA035-18 (Alpine part), CROAA036-18 (Continental part), CROAA037-18 (Alpine part), CROAA064-18 (Mediterranean part), CROTR057-19 (Alpine part), CROTR063-19 (Mediterranean part), CROTR090-19 (Mediterranean part).

**Literature data.**Cerjanec et al. (2020); Ćukušić (2019); Ivković et al. (2023); Kučinić et al. (2008, 2017b, 2020a); Marinković-Gospodnetić (1979); Neu et al. (2018); Previšić et al. (2007a, 2010, 2014a); Vučković et al. (2011, 2021); Waringer et al. (2009); Žalac and Kučinić (2022).

#### ﻿Genus *Synagapetus* McLachlan, 1879

***Synagapetus krawanyi* Ulmer, 1938**

**Distribution in Croatia.** Continental part, Alpine part, Mediterranean part.

**General distribution.** Croatia, Eastern Alps, Hungary.

**Ecoregion.**ER5, ER11.

**Drainage.**AS, BS.

**BOLD ID.**CROTR153-19 (Continental part), CROTR213-19 (Continental part).

**Literature data.**Ćukušić (2019); Ivković et al. (2013, 2023); Kučinić et al. (2008, 2017b); Malicky (2014a); Marinković-Gospodnetić (1979); Neu et al. (2018); Previšić et al. (2007a, 2010); Vučković et al. (2011, 2021).

***Synagapetus moselyi* (Ulmer, 1938)**

**Distribution in Croatia.** Continental part.

**General distribution.** Bulgaria, Czechia, Eastern Alps (Austria), Hungary, Romania, Slovakia, southern Europe.

**Ecoregion.**ER11.

**Drainage.**BS.

**BOLD ID.**NIPAG003-17 (Continental part).

**Literature data.**Ćukušić (2019).

#### ﻿﻿Family Goeridae Ulmer, 1903

**Genus *Goera* Stephens, 1829**

***Goera pilosa* (Fabricius, 1775)**

**Synonyms.***Trichostomum auratum* Burmeister, 1839; *Trichostoma capillata* Pictet, 1834; *Silo flavipes* Curtis, 1833; *Lasiostoma fulvum* Rambur, 1842; *Trichostoma fuscicorne* Pictet, 1834; *Phryganea vulgata* Fourcroy, 1785.

**Distribution in Croatia.** Continental part, Alpine part.

**General distribution.** Widely distributed in Europe, including European part of Russia. The species is also recorded in Turkey.

**Ecoregion.**ER5, ER11.

**Drainage.**BS.

**BOLD ID.**CROAA073-18 (Continental part), CROTR118-19 (Continental part), CROTR220-19 (Continental part), CROTR243-19 (Continental part), CROTR286-19 (Continental part).

**Literature data.**Cerjanec et al. (2020); Ćukušić (2019); Ivković et al. (2023); Kučinić et al. (2010, 2017b, 2020b); Malicky (2009); Neu et al. (2018); Previšić et al. (2007b, 2010, 2012).

#### ﻿Genus *Lithax* McLachlan, 1876

***Lithax niger* (Hagen, 1859)**

**Synonym.***Aspatherium frigidum* Meyer-Duer, 1864.

**Distribution in Croatia.** Continental part, Alpine part.

**General distribution.** Albania, Alpine region, Belgium, Bosnia and Herzegovina, Croatia, Czechia, France, Germany, Luxembourg, Montenegro, Romania, Slovakia, Ukraine.

**Ecoregion.**ER5.

**Drainage.**BS.

**BOLD ID.** No DNA barcoded specimens from Croatia.

**Literature data.**Ivković et al. (2013, 2023); Kučinić et al. (2008, 2017b); Malicky (2014a); Neu et al. (2018); Previšić and Popijač (2010); Previšić et al. (2007a, 2010).

***Lithax obscurus* (Hagen, 1859)**

**Synonym.***Lithax fuscipes* Rostock, 1879.

**Distribution in Croatia.** Continental part, Alpine part.

**General distribution.** Austria, Balkan Peninsula, Belgium, Croatia, Czechia, Denmark, Estonia, France, Germany, Luxembourg, Slovakia, Spain, Switzerland, Ukraine.

**Ecoregion.**ER5, ER11.

**Drainage.**BS.

**BOLD ID.**CROTR330-21 (Continental part).

**Literature data.**Klapálek (1906); Langhoffer (1912); Neu et al. (2018); Radovanović (1935).

#### ﻿Genus *Silo* Curtis, 1830

***Silo nigricornis* (Pictet, 1834)**

**Synonyms.***Goera dalmatina* Kolenati, 1848; *Silo fumipennis* McLachlan, 1865.

**Distribution in Croatia.** Continental part, Alpine part, Mediterranean part.

**General distribution.** Alpine region, Benelux, Bosnia and Herzegovina, British Islands, Croatia, Czechia, Denmark, France, Germany, Hungary, Iberian Peninsula, Italy, Poland, Ukraine.

**Ecoregion.**ER5, ER11.

**Drainage.**AS, BS.

**BOLD ID.**CROTR288-19 (Mediterranean part), CROTR355-21 (Mediterranean part) CROTR363-21 (Mediterranean part), CROTR364-21 (Mediterranean part), CROTR365-21 (Mediterranean part), CROTR366-21 (Mediterranean part), CROTR367-21 (Mediterranean part), CROTR368-21 (Mediterranean part), CROTR369-21 (Mediterranean part), CROTR370-21 (Mediterranean part), CROTR371-21 (Mediterranean part).

**Literature data.**Ćukušić (2019); Kučinić et al. (2021); Neu et al. (2018); Oláh (2010); Previšić et al. (2007b); Radovanović (1935); Žalac and Kučinić (2022).

***Silo pallipes* (Fabricius, 1781)**

**Synonyms.***Silo duplex* Hagen, 1964; *Aspatherium fuscicorne* sensu Kolenati, 1848, not Pictet, in part; *Limnophilus iridion* Waga, 1857; *Silo minutus* Kolenati, 1848; *Trichostoma picicorne* Pictet, 1834; *Goera vulgata* Stephens, 1836.

**Distribution in Croatia.** Continental part, Alpine part, Mediterranean part.

**General distribution.** Widely distributed in Europe, including European part of Russia.

**Ecoregion.**ER5, ER11.

**Drainage.**AS, BS.

**BOLD ID.**CROAA132-18 (Mediterranean part), CROTR065-19 (Alpine part), CROTR287-19 (Mediterranean part), CROTR381-22 (Continental part).

**Literature data.**Ćukušić (2019); Ivković et al. (2013, 2023); Kučinić et al. (2017b, 2020a); Malicky (2009, 2014a); Marinković-Gospodnetić (1979); Neu et al. (2018); Previšić and Popijač (2010); Previšić et al. (2007a, 2010).

***Silo piceus* (Brauer, 1857)**

**Synonyms.***Aspatherium fuscicorne* sensu Kolenati 1848, not Pictet 1834, in part; *Aspatherium medium* Meyer-Duer, 1864; *Aspatherium obtusus* Hagen, 1864; *Ptilocolepus turbidus* Dziedzielewicz, 1883; *Silo vulgatus* Hagen, 1859.

**Distribution in Croatia.** Continental part, Alpine part, Mediterranean part.

**General distribution.** Alpine region, Balkan Peninsula, Belgium, Croatia, Czechia, France, Germany, Hungary, Luxembourg, Poland, Slovakia, Spain, Ukraine.

**Ecoregion.**ER5, ER11.

**Drainage.**AS, BS.

**BOLD ID.**CROAA049-18 (Continental part), CROTR100-19 (Mediterranean part), CROTR101-19 (Mediterranean part), CROTR181-19 (Alpine part).

**Literature data.**Cerjanec et al. (2020); Ćukušić (2019); Graf et al. (2008); Kučinić et al. (2011, 2020b); Malicky (2014a); Neu et al. (2018); Previšić and Popijač (2010); Previšić et al. (2012, 2013c); Vučković et al. (2011, 2021).

#### ﻿﻿Family Hydroptilidae Stephens, 1834

**Genus *Agraylea* Curtis, 1834**

***Agraylea sexmaculata* Curtis, 1834**

**Synonyms.***Hydroptila flabellifera* Bremi, 1864 in part; *Agraylea pallidula* McLachlan, 1875.

**Distribution in Croatia.** Continental part, Mediterranean part.

**General distribution.** Widely distributed in Europe, including European part of Russia. The species is also recorded in Iran and Turkey.

**Ecoregion.**ER5, ER11.

**Drainage.**AS, BS.

**BOLD ID.**CROTR093-19 (Mediterranean part), CROTR249-19 (Continental part), CROTR253-19 (Continental part).

**Literature data.**Ćukušić (2019); Graf et al. (2008); Kučinić et al. (2020b); Malicky (2014a); Neu et al. (2018); Vučković et al. (2021).

#### ﻿Genus *Allotrichia* McLachlan, 1880

***Allotrichia pallicornis* (Eaton, 1873)**

**Synonym.***Allotrichia tauri* Jacquemart, 1965.

**Distribution in Croatia.** Alpine part, Mediterranean part.

**General distribution.** Alpine region, Apennine Peninsula including Sicily, Balkan Peninsula, Belgium, British Islands, Croatia, Denmark, Finland, France with Corsica, Germany, Hungary, Lithuania, Luxembourg, Slovakia, Ukraine. The species is also recorded in Iran and Turkey.

**Ecoregion.**ER5, ER11.

**Drainage.**AS, BS.

**BOLD ID.**CROAA100-18 (Alpine part), CROTR092-19 (Mediterranean part).

**Literature data.**Ćukušić (2019); Kučinić et al. (2019b); Neu et al. (2018); Ridl et al. (2017).

#### ﻿Genus *Hydroptila* Dalman, 1819

***Hydroptila cognata* Mosely, 1930**

**Distribution in Croatia.** Alpine part.

**General distribution.** Croatia, France, Italy, Slovenia, Spain.

**Ecoregion.**ER5.

**Drainage.**BS.

**BOLD ID.** No DNA barcoded specimens from Croatia.

**Literature data.**Ivković et al. (2023); Kučinić et al. (2017b); Malicky (2014a); Neu et al. (2018); Šemnički et al. (2011, 2012).

***Hydroptila forcipata* (Eaton, 1873)**

**Distribution in Croatia.** Continental part, Alpine part, Mediterranean part.

**General distribution.** Widely distributed in Europe, including European part of Russia (along the Ural Mountains). The species is also recorded in Turkey.

**Ecoregion.**ER5.

**Drainage.**AS, BS.

**BOLD ID.**CROAA093-18 (Alpine part), CROTR095-19 (Mediterranean part), CROTR121-19 (Continental part), CROTR127-19 (Mediterranean part), CROTR131-19 (Mediterranean part).

**Literature data.**Cerjanec et al. (2020); Ćukušić (2019); Kučinić et al. (2015, 2019b); Neu et al. (2018).

***Hydroptila lotensis* Mosely, 1930**

**Distribution in Croatia.** Continental part, Alpine part.

**General distribution.** Balkan Peninsula, Belgium, Croatia, Czechia, Eastern Alps, Great Britain, France, Finland, Germany, Hungary, Poland, Spain, Slovakia, Ukraine. The species is also recorded in Iran and Turkey.

**Ecoregion.**ER5, ER11.

**Drainage.**BS.

**BOLD ID.**CROTR056-19 (Continental part), CROTR152-19 (Continental part), CROTR251-19 (Continental part), CROTR255-19 (Continental part).

**Literature data.**Cerjanec et al. (2020); Ćukušić (2019); Kučinić et al. (2020a, 2020b); Neu et al. (2018).

***Hydroptila martini* Marshall, 1977**

**Synonym.***Hydroptila occulta* sensu auct. nec Eaton (Marshall, 1979).

**Distribution in Croatia.** Continental part, Alpine part, Mediterranean part.

**General distribution.** Alpine region, Apennine Peninsula with Sicily, British Islands, Czechia, Denmark, France, Germany, Gotland (Island of Sweeden), Montenegro, Slovakia, Spain, Poland.

**Ecoregion.**ER5, ER11.

**Drainage.**AS, BS.

**BOLD ID.**CROAA094-18 (Alpine part), CROTR087-19 (Continental part), CROTR088-19 (Continental part), CROTR141-19 (Continental part), CROTR170-19 (Mediterranean part).

**Literature data.**Ćukušić (2019); Kučinić et al. (2019a, 2020a, 2021).

***Hydroptila occulta* (Eaton, 1873)**

**Synonyms.***Hydroptila insignis* Martynov, 1927; *Hydroptila kimminsi* Mosely, 1930; *Hydroptila parthava* Schmid, 1959.

**Distribution in Croatia.** Continental part, Alpine part.

**General distribution.** Widely distributed in Europe, including European part of Russia. The species is also recorded in Turkey.

**Ecoregion.**ER5, ER11.

**Drainage.**BS.

**BOLD ID.** No DNA barcoded specimens from Croatia.

**Literature data.**Ivković et al. (2023); Kučinić et al. (2017b); Malicky (2014a); Neu et al. (2018); Šemnički et al. (2011, 2012).

***Hydroptila phaon* Malicky, 1976**

**Distribution in Croatia.** Alpine part, Mediterranean part.

**General distribution.** Apennine Peninsula with Sicily, Greece, France.

**Ecoregion.**ER5.

**Drainage.**AS, BS.

**BOLD ID.**CROAA111-18 (Mediterranean part), CROTR232-19 (Mediterranean part), CROTR268-19 (Alpine part).

**Literature data.**Ćukušić (2019); Kučinić et al. (2020a).

***Hydroptila rheni* Ris, 1896**

**Distribution in Croatia.** Alpine part.

**General distribution.** Croatia, France, Switzerland.

**Ecoregion.**ER5.

**Drainage.**BS.

**BOLD ID.** No DNA barcoded specimens from Croatia.

**Literature data.**Ivković et al. (2023); Kučinić et al. (2017b); Neu et al. (2018); Previšić et al. (2013b).

***Hydroptila simulans* Mosely, 1920**

**Distribution in Croatia.** Mediterranean part.

**General distribution.** Widely distributed in Europe, including European part of Russia (along the Ural Mountains). The species is also recorded in Iran, Israel, Lebanon, and Turkey.

**Ecoregion.**ER5.

**Drainage.**AS.

**BOLD ID.**CROTR129-19 (Mediterranean part).

**Literature data.**Ćukušić (2019); Kučinić et al. (2019b).

***Hydroptila sparsa* Curtis, 1834**

**Synonym.***Hydroptila brunneicornis* Stephens, 1836.

**Distribution in Croatia.** Continental part, Mediterranean part.

**General distribution.** Widely distributed in Europe, including European part of Russia. The species is also recorded in Iran, Lebanon, and Turkey.

**Ecoregion.**ER5, ER11.

**Drainage.**AS, BS.

**BOLD ID.**CROAA124-18 (Mediterranean part), CROTR156-19 (Mediterranean part), CROTR185-19 (Mediterranean part), CROTR196-19 (Mediterranean part).

**Literature data.**Ćukušić (2019); Kučinić et al. (2011, 2019b, 2021); Malicky (2014a); Neu et al. (2018); Previšić et al. (2007b); Ridl et al. (2017).

***Hydroptila tigurina* Ris, 1894**

**Distribution in Croatia.** Continental part, Alpine part.

**General distribution.** British Islands, Greece, Italy and Sicily, Spain, Switzerland.

**Ecoregion.**ER5, ER11.

**Drainage.**AS, BS.

**BOLD ID.**CROTR146-19 (Continental part), CROTR182-19 (Continental part), CROTR183-19 (Continental part).

**Literature data.**Cerjanec et al. (2020); Ćukušić (2019).

***Hydroptila tineoides* Dalman, 1819**

**Synonyms.***Phrixocoma femoralis* Eaton, 1873; *Hydroptila longispina* McLachlan, 1884.

**Distribution in Croatia.** Continental part, Alpine part, Mediterranean part.

**General distribution.** Widely distributed in Europe, including European part of Russia (along the Ural Mountains). The species is also recorded in Mongolia and Turkey.

**Ecoregion.**ER5, ER11.

**Drainage.**AS, BS.

**BOLD ID.**CROTR085-19 (Continental part), CROTR102-19 (Continental part), CROTR139-19 (Continental part), CROTR231-19 (Continental part).

**Literature data.**Cerjanec et al. (2020); Ćukušić (2019); Ivković et al. (2023); Kučinić et al. (2019a, 2020a, 2020b); Neu et al. (2018); Vučković et al. (2021).

***Hydroptila vectis* Curtis, 1834**

**Synonyms.**Hydroptila maclachlani var. corsicana Mosely, 1930; *Hydroptila maclachlani* Klapálek, 1891.

**Distribution in Croatia.** Continental part, Mediterranean part.

**General distribution.** Widely distributed in Europe, including European part of Russia (along the Ural Mountains). The species is also recorded in Israel, Lebanon, and Turkey.

**Ecoregion.**ER5, ER11.

**Drainage.**AS, BS.

**BOLD ID.**CROAA091-18 (Continental part), CROTR168-19 (Mediterranean part), CROTR175-19 (Mediterranean part), CROTR215-19 (Mediterranean part).

**Literature data.**Ćukušić (2019); Kučinić et al. (2021); Neu et al. (2018); Previšić et al. (2007b).

***Hydroptila vichtaspa* Schmid, 1959**

**Distribution in Croatia.** Mediterranean part.

**General distribution.** Balearic Islands (Spain), Croatia, Cyprus, Czechia, France, Greece, Italy, Portugal, Slovakia. The species is also recorded in Iran and Turkey.

**Ecoregion.**ER5.

**Drainage.**AS.

**BOLD ID.** No DNA barcoded specimens from Croatia.

**Literature data.**Malicky (2014a); Neu et al. (2018).

#### ﻿Genus *Ithytrichia* Eaton, 1873

***Ithytrichia lamellaris* Eaton, 1873**

**Synonym.***Hydroptila brunneicornis* Pictet, 1834.

**Distribution in Croatia.** Continental part, Alpine part, Mediterranean part.

**General distribution.** Widely distributed in Europe, including European part of Russia. The species is also recorded in Lebanon and Russian Far East.

**Ecoregion.**ER5, ER11.

**Drainage.**AS, BS.

**BOLD ID.**CROAA067-18 (Mediterranean part), CROTR138-19 (Continental part), CROTR143-19 (Continental part), CROTR200-19 (Continental part).

**Literature data.**Cerjanec et al. (2020); Ćukušić (2019); Ćukušić et al. (2017); Neu et al. (2018).

#### ﻿Genus *Microptila* Ris, 1897

***Microptila minutissima* Ris, 1897**

**Distribution in Croatia.** Alpine part.

**General distribution.** Alpine area, Croatia, Greece.

**Ecoregion.**ER5.

**Drainage.**BS.

BOLD: No DNA barcoded specimens from Croatia.

**Literature data.**Neu et al. (2018).

#### ﻿Genus *Orthotrichia* Eaton, 1873

***Orthotrichia angustella* (McLachlan, 1865)**

**Synonym.***Hydroptila brunneicornis* Pictet, 1834, in part.

**Distribution in Croatia.** Continental part, Alpine part.

**General distribution.** Apennine Peninsula with Sicily and Sardinia, British Islands, Bulgaria, Denmark, Eastern Alps, Germany, Iberian Peninsula, Lithuania, Netherlands, Poland, Romania, Sweden. The species is also recorded in Canary Islands, Morocco, and Tunisia.

**Ecoregion.**ER5, ER11.

**Drainage.**BS.

**BOLD ID.**CROAA068-18 (Continental part).

**Literature data.**Cerjanec et al. (2020); Ćukušić (2019); Neu et al. (2018).

***Orthotrichia costalis* (Curtis, 1834)**

**Synonym.***Orthotrichia tetensii* Kolbe, 1887.

**Distribution in Croatia.** Continental part, Mediterranean part.

**General distribution.** Widely distributed in Europe, including European part of Russia. The species is also recorded in China, Japan, Russian Far East, Tunisia, and Turkey.

**Ecoregion.**ER5, ER11.

**Drainage.**AS, BS.

**BOLD ID.**CROTR184-19 (Mediterranean part), CROTR224-19 (Mediterranean part), CROTR252-19 (Mediterranean part).

**Literature data.**Ćukušić (2019); Kučinić et al. (2019b); Neu et al. (2018).

***Orthotrichia tragetti* Mosely, 1930**

**Distribution in Croatia.** Continental part, Mediterranean part.

**General distribution.** Alpine region, Benelux, Bulgaria, Croatia, Czechia, Finland, France, Germany, Great Britain, Greece, Hungary, Italy, Poland, Romania, Slovakia, Sweeden, Ukraine. The species is also recorded in China, Japan, Russian Far East, Vietnam.

**Ecoregion.**ER5, ER11.

**Drainage.**AS, BS.

**BOLD ID.**CROAA069-18 (Mediterranean part).

**Literature data.**Ćukušić (2019); Neu et al. (2018); Vrućina et al. (2016).

#### ﻿Genus *Oxyethira* Eaton, 1873

***Oxyethira falcata* Morton, 1893**

**Synonyms.***Oxyethira assia* Botosaneanu & Moubayed, 1985; *Oxyethira bidentata* Nybom, 1948; *Oxyethira boreella* Svensson & Tjeder, 1975; *Oxyethira dentata* Nybom, 1954; *Oxyethira rhodani* Schmid, 1947.

**Distribution in Croatia.** Continental part, Mediterranean part.

**General distribution.** Widely distributed in Europe, including European part of Russia. The species is also recorded in: China, Iran, Jordan, Lebanon, Tunisia, and Turkey.

**Ecoregion.**ER5, ER11.

**Drainage.**AS, BS.

**BOLD ID.**CROTR166-19 (Mediterranean part), CROTR167-19 (Mediterranean part), CROTR169-19 (Mediterranean part).

**Literature data.**Ćukušić (2019); Kučinić et al. (2021); Neu et al. (2018).

***Oxyethira flavicornis* (Pictet, 1834)**

**Synonym.***Oxyethira costalis* Eaton, 1873.

**Distribution in Croatia.** Continental part, Alpine part.

**General distribution.** Widely distributed in Europe, including European part of Russia. The species is recorded in Egypt and Mongolia.

**Ecoregion.**ER5, ER11.

**Drainage.**AS, BS.

**BOLD ID.**CROAA050-18 (Continental part), CROAA095-18 (Alpine part), CROTR062-19 (Alpine part), CROTR103-19 (Alpine part).

**Literature data.**Ćukušić (2019); Neu et al. (2018).

#### ﻿﻿Family Lepidostomatidae Ulmer, 1903

**Genus *Crunoecia* McLachlan, 1876**

***Crunoecia irrorata* (Curtis, 1834)**

**Synonyms.***Sericostoma hirtum* Pictet, 1834; *Mormonia minor* Stephens, 1836.

**Distribution in Croatia.** Continental part, Alpine part.

**General distribution.** Widely distributed in Europe, including European part of Russia.

**Ecoregion.**ER5, ER11.

**Drainage.**BS.

**BOLD ID.** No DNA barcoded specimens from Croatia.

**Literature data.**Ivković et al. (2023); Neu et al. (2018); Žalac and Kučinić (2022).

***Crunoecia kempnyi* Morton, 1901**

**Distribution in Croatia.** Alpine part.

**General distribution.** Alpine region, Bosnia and Herzegovina, Croatia.

**Ecoregion.**ER5.

**Drainage.**BS.

**BOLD ID.**CROTR074-19 (Alpine part).

**Literature data.**Ćukušić (2019); Ivković et al. (2023); Kučinić et al. (2017b, 2020a); Malicky (2014a); Neu et al. (2018); Previšić et al. (2010); Žalac and Kučinić (2022).

#### ﻿Genus *Lepidostoma* Rambur, 1842

***Lepidostoma basale* (Kolenati, 1848)**

**Synonyms.***Lasiocephala bifida* Decamps, 1972; *Sericostoma hirtum* Pictet, 1834 in part, not *Phryganea hirta* Fabricius; *Goera irrorata* Kolenati, 1859, not *Goera irrorata* Curtis; *Lasiocephala taurus* Costa, 1857.

**Distribution in Croatia.** Continental part, Mediterranean part.

**General distribution.** Widely distributed in Europe.

**Ecoregion.**ER5, ER11.

**Drainage.**AS, BS.

**BOLD ID.**CROAA024-18 (Continental part), CROAA025-18 (Continental part), CROTR122-19 (Mediterranean part).

**Literature data.**Ćukušić (2019); Kučinić et al. (2020a, 2020b); Malicky (2014a); Neu et al. (2018); Vučković et al. (2021).

***Lepidostoma hirtum* (Fabricius, 1775)**

**Synonyms.***Mormonia fimbriata* Pictet, 1865; *Mormonia gracilicornis* Curtis, 1834; *Mormonia immaculata* Stephens, 1836; *Lepidostoma lapponicum* Siltala, 1908; *Mormonia maculicornis* Curtis, 1834; *Mormonia nigromaculata* Stephens, 1836; *Ayabeopsyche nipponica* Tsuda, 1942; *Lepidostoma sericeum* Rambur, 1842; *Lepidostoma squamulosum* Rambur, 1842; *Lepidostoma* villosum Rambur, 1842.

**Distribution in Croatia.** Continental part, Alpine part, Mediterranean part.

**General distribution.** Widely distributed in Europe, including European part of Russia (along the Ural Mountains). The species is also recorded in Turkey, as well as subspecies *Lepidostoma hirtum orientalis* Mey & Jung, 1986 with its distribution also in Armenia and Iran.

**Ecoregion.**ER5, ER11.

**Drainage.**AS, BS.

**BOLD ID.**CROAA126-18 (Continental part), CROTR053-19 (Continental part), CROTR124-19 (Mediterranean part), CROTR132-19 (Mediterranean part), CROTR218-19 (Alpine part), CROTR235-19 (Mediterranean part), CROTR244-19 (Mediterranean part).

**Literature data.**Cerjanec et al. (2020); Ćukušić (2019); Graf et al. (2008); Ivković et al. (2023); Klapálek (1906); Kučinić et al. (2008, 2010, 2011, 2017b, 2019a, 2019b, 2020a, 2020b); Malicky (2014a); Neu et al. (2018); Previšić and Popijač (2010); Previšić et al. (2007a, 2010); Ridl et al. (2017); Šemnički et al. (2011, 2012); Vučković et al. (2021); Žalac and Kučinić (2022).

#### ﻿﻿Family Leptoceridae Leach in Brewster, 1815

**Genus *Adicella* McLachlan, 1877**

***Adicella balcanica* Botosaneanu & Novak, 1965**

**Distribution in Croatia.** Mediterranean part.

**General distribution.** Balkan Peninsula and Eastern Alps.

**Ecoregion.**ER5.

**Drainage.**AS.

**BOLD ID.**GBMIN91695-17 (Mediterranean part), NIP010-16 (Mediterranean part).

**Literature data.**Ćukušić (2019); Ćukušić et al. (2017).

***Adicella cremisa* Malicky, 1972**

**Distribution in Croatia.** Continental part, Mediterranean part.

**General distribution.** Alpine region, Italy.

**Ecoregion.**ER5, ER11.

**Drainage.**AS, BS.

**BOLD ID.**GBMH17848-19 (Mediterranean part), GBMIN91696-17 (Continental part), NIP008-16 (Continental part), NIP009-16 (Mediterranean part).

**Literature data.**Ćukušić (2019); Ćukušić et al. (2017).

***Adicella filicornis* (Pictet, 1834)**

**Synonyms.***Setodes moestella* McLachlan, 1868; *Leptocerus attenuatus* Stephens, 1836; *Setodes eremita* Stein, 1873; *Erotesis italica* Navas, 1934.

**Distribution in Croatia.** Alpine part, Mediterranean part.

**General distribution.** Alpine region, Balkan Peninsula, Belgium, Czechia, France, Germany, Great Britain, Hungary, Italy, Luxembourg, Poland, Slovakia, Ukraine.

**Ecoregion.**ER5.

**Drainage.**AS, BS.

**BOLD ID.**CROTR349-21 (Mediterranean part), CROTR351-21 (Mediterranean part).

**Literature data.**Ivković et al. (2023); Kučinić et al. (2017b, 2021); Previšić et al. (2010); Vučković et al. (2021); Žalac and Kučinić (2022).

***Adicella reducta* (McLachlan, 1865)**

**Synonyms.***Leptocerus bicolor* Stephens, 1836; *Mystacides ferruginea* Pictet, 1865; *Adicella noguerana* Navas, 1919.

**Distribution in Croatia.** Continental part.

**General distribution.** Widely distributed in Europe. The species is also recorded in Turkey.

**Ecoregion.**ER11.

**Drainage.**BS.

**BOLD ID.** No DNA barcoded specimens from Croatia.

**Literature data.**Neu et al. (2018); Previšić et al. (2013a).

***Adicella syriaca* Ulmer, 1907**

**Distribution in Croatia.** Continental part, Alpine part, Mediterranean part.

**General distribution.** Balkan Peninsula, Croatia, Slovenia. The species is also recorded in Turkey and Lebanon.

**Ecoregion.**ER5, ER11.

**Drainage.**AS, BS.

**BOLD ID.** No DNA barcoded specimens from Croatia.

**Literature data.**Ćukušić (2019); Graf et al. (2008); Ivković et al. (2023); Kučinić et al. (2017b); Malicky (2014a); Neu et al. (2018); Previšić et al. (2010); Ridl et al. (2017); Šemnički et al. (2011, 2012); Vučković et al. (2021).

#### ﻿Genus *Athripsodes* Billberg, 1820

***Athripsodes albifrons* (Linnaeus, 1758)**

**Synonyms.***Leptocerus albifrons* Linnaeus, 1758; *Leptocerus interjectus* McLachlan, 1881; *Phryganea interrupta* Donovan, 1813.

**Distribution in Croatia.** Continental part, Mediterranean part.

**General distribution.** Widely distributed in Europe, including European part of Russia (along the Ural Mountains). The species is also recorded in Turkey.

**Ecoregion.**ER5, ER11.

**Drainage.**AS, BS.

**BOLD ID.**CROAA011-18 (Continental part), CROTR123-19 (Mediterranean part).

**Literature data.**Ćukušić (2019); Kučinić et al. (2010, 2011, 2019b, 2020b); Malicky (2014a); Neu et al. (2018); Radovanović (1935); Ridl et al. (2017); Vučković et al. (2021).

***Athripsodes aterrimus* (Stephens, 1836)**

**Synonyms.***Leptocerus caliginosus* Stephens, 1836; *Phryganea nigrafasciata* Retzius, 1783.

**Distribution in Croatia.** Continental part, Alpine part, Mediterranean part.

**General distribution.** Widely distributed in Europe, including European part of Russia (along the Ural Mountains).

**Ecoregion.**ER5, ER11.

**Drainage.**AS, BS.

**BOLD ID.**CROAA054-18 (Continental part), CROTR037-19 (Mediterranean part).

**Literature data.**Ćukušić (2019); Ivković et al. (2023); Kučinić et al. (2008, 2017b); Malicky (2009, 2014a); Neu et al. (2018); Previšić et al. (2007b, 2010, 2013c).

***Athripsodes bilineatus* (Linnaeus, 1758)**

**Synonyms.***Leptocerus affinis* Stephens, 1836; *Phryganea bifasciata* Olivier, 1791; *Leptocerus fulvoguttatus* Mosely, 1935; *Phryganea gallata* Fourcroy, 1785; *Leptocerus noguerensis* Navas, 1930.

**Distribution in Croatia.** Continental part, Alpine part, Mediterranean part.

**General distribution.** Widely distributed in Europe (excluding Scandinavian Peninsula), including European part of Russia (along the Ural Mountains). The subspecies *Athripsodes bilineatus aegeus* Malicky, 1999 is recorded in Greece and Western Turkey.

**Ecoregion.**ER5, ER11.

**Drainage.**BS.

**BOLD ID.**CROAA012-18 (Continental part).

**Literature data.**Ćukušić (2019); Ćukušić et al. (2017); Ivković et al. (2023); Kučinić et al. (2017b, 2020a, 2020b); Malicky (2009, 2014a); Neu et al. (2018); Previšić et al. (2010); Šemnički et al. (2011, 2012).

***Athripsodes cinereus* (Curtis, 1834)**

**Synonyms.***Phryganea annulata* Gmelin, 1789; *Mystacida rufina* Rambur, 1842; *Leptocerus seminiger* Stephens, 1836.

**Distribution in Croatia.** Continental part, Alpine, part, Mediterranean part.

**General distribution.** Widely distributed in Europe, including European part of Russia.

**Ecoregion.**ER5, ER11.

**Drainage.**AS, BS.

**BOLD ID.**CROAA001-18 (Continental part), CROTR033-19 (Alpine part), CROTR049-19 (Continental part), CROTR060-19 (Continental part), CROTR114-19 (Continental part), CROTR120-19 (Continental part).

**Literature data.**Ćukušić (2019); Ivković et al. (2023); Kučinić et al. (2008, 2011, 2017b, 2020a); Malicky (2014a); Neu et al. (2018); Previšić et al. (2007b, 2010); Ridl et al. (2017); Šemnički et al. (2011, 2012); Žalac and Kučinić (2022).

***Athripsodes dalmatinus* Malicky, 1980**

**Distribution in Croatia.** Mediterranean part.

**General distribution.** Bosnia and Herzegovina, Croatia, Montenegro, Slovenia.

**Ecoregion.**ER5.

**Drainage.**AS.

**BOLD ID.**CROAA066-18 (Mediterranean part).

**Literature data.**Ćukušić (2019); Graf et al. (2008); Kučinić et al. (2011); Malicky (1980); Neu et al. (2018); Vučković et al. (2021).

#### ﻿Genus *Ceraclea* Stephens, 1829

***Ceraclea albimacula* (Rambur, 1842)**

**Synonyms.***Leptocerus alboguttatus* Hagen, 1860; *Leptocerus biwaensis* Tsuda & Kuwayama, 1950; *Ceraclea morsei* Kumanski, 1991; *Leptocerus spinosus* Tsuda, 1942.

**Distribution in Croatia.** Continental part, Alpine part, Mediterranean part.

**General distribution.** Widely distributed in Europe, including European part of Russia. The species is also recorded in China, Korea, Mongolia, Russian Far East, and Turkey.

**Ecoregion.**ER5.

**Drainage.**AS, BS.

**BOLD ID.** No DNA barcoded specimen from Croatia.

**Literature data.**Cerjanec et al. (2020); Ćukušić (2019); Kučinić et al. (2011); Neu et al. (2018); Vučković et al. (2021).

***Ceraclea annulicornis* (Stephens, 1836)**

**Synonyms.***Leptocerus futilis* Banks, 1914; *Leptocerus lugens* Hagen, 1861; *Mystacides perfusus* Kolenati, 1858; *Leptocerus recurvatus* Banks, 1908.

**Distribution in Croatia.** Continental part, Alpine part.

**General distribution.** Alpine region, Benelux, British Islands, Bulgaria, Croatia, Czechia, Estonia, France, Germany, Poland, Romania, Scandinavian Peninsula, Serbia, Ukraine, including European part of Russia. The species is also recorded in Mongolia, North America, and Russian Far East.

**Ecoregion.**ER5, ER11.

**Drainage.**BS.

**BOLD ID.** No DNA barcoded specimens from Croatia.

**Literature data.**Ivković et al. (2023); Kučinić et al. (2017b); Malicky (2009, 2014a); Neu et al. (2018); Šemnički et al. (2011, 2012).

***Ceraclea dissimilis* (Stephens, 1836)**

**Synonyms.***Leptocerus assimilis* Stephens, 1836; *Leptocerus norfolki* Navas, 1917; *Mystacides sericeus* Kolenati, 1858; *Mystacides uniguttatus* Pictet, 1834; *Mystacida vetula* Rambur, 1842.

**Distribution in Croatia.** Continental part, Alpine part, Mediterranean part.

**General distribution.** Widely distributed in Europe, including European part of Russia.

**Ecoregion.**ER5, ER11.

**Drainage.**AS, BS.

**BOLD ID.**CROAA020-18 (Continental part), CROTR237-19 (Continental part), CROTR254-19 (Mediterranean part).

**Literature data.**Cerjanec et al. (2020); Ćukušić (2019); Graf et al. (2008); Ivković et al. (2023); Kučinić et al. (2008, 2010, 2011, 2017b, 2019b, 2020b); Malicky (2014a); Neu et al. (2018); Previšić et al. (2010); Ridl et al. (2017); Šemnički et al. (2011, 2012); Vučković et al. (2021).

***Ceraclea riparia* (Albarda, 1874)**

**Distribution in Croatia.** Continental part.

**General distribution.** Bulgaria, Croatia, Finland, France, Germany, Greece, Hungary, Latvia, Netherlands, Serbia, Switzerland, Romania, including European part of Russia (along the Ural Mountains). The species is also recorded in China, Russian Far East, and Turkey.

**Ecoregion.**ER5.

**Drainage.**BS.

**BOLD ID.**CROTR112-19 (Continental part), CROTR128-19 (Continental part).

**Literature data.**Cerjanec et al. (2020); Ćukušić (2019); Kučinić et al. (2015); Neu et al. (2018).

***Ceraclea senilis* (Burmeister, 1839)**

**Synonyms.***Leptocerus nygmaticus* Navas, 1917; *Mystacides ochraceus* Kolenati, 1858; *Leptocerus spongillae* Garbini,1894.

**Distribution in Croatia.** Continental part.

**General distribution.** Widely distributed in Europe (except in Iberian Peninsula and Iceland), including European part of Russia.

**Ecoregion.**ER11.

**Drainage.**BS.

**BOLD ID.** No DNA barcoded specimens from Croatia.

**Literature data.**Klapálek (1906); Neu et al. (2018).

#### ﻿Genus *Erotesis* McLachlan, 1877

***Erotesis baltica* McLachlan, 1877**

**Distribution in Croatia.** Mediterranean part.

**General distribution.** Alpine region, Belarus, British Islands, Croatia, Czechia, Denmark, Estonia, Finland, Germany, Hungary, Italy, Lithuania, Netherlands, Poland, Slovakia, Sweden.

**Ecoregion.**ER5.

**Drainage.**AS.

**BOLD ID.** No DNA barcoded specimens from Croatia.

**Literature data.**Kučinić et al. (2011).

#### ﻿Genus *Leptocerus* Leach, 1815

***Leptocerus interruptus* (Fabricius, 1775)**

**Synonyms.***Leptocerus albonotatus* von Heyden, 1896 (1895-1896); *Phryganea trifasciatus* von Paula Schrank, 1802; *Mystacida trifasciata* Thevenet, 1871.

**Distribution in Croatia.** Continental part, Alpine part, Mediterranean part.

**General distribution.** Balkan Peninsula, Belarus, Benelux, Croatia, Eastern Alps, Estonia, France, Germany, Great Britain, Lithuania, Poland, including European part of Russia. The species is also recorded in Turkey.

**Ecoregion.**ER5, ER11.

**Drainage.**AS, BS.

**BOLD ID.** No DNA barcoded specimens from Croatia.

**Literature data.**Cerjanec et al. (2020); Ćukušić (2019); Malicky (2009, 2014a); Neu et al. (2018); Previšić and Popijač (2010).

***Leptocerus tineiformis* Curtis, 1834**

**Synonyms.***Setodes aspersellus* Rambur, 1842; *Leptocerus elongatus* Stephens, 1836; *Phryganea longicornis* von Paula Schrank, 1802.

**Distribution in Croatia.** Continental part, Mediterranean part.

**General distribution.** Widely distributed in Europe, including European part of Russia. The species is also recorded in Turkey.

**Ecoregion.**ER5, ER11.

**Drainage.**AS, BS.

**BOLD ID.**CROAA092-18 (Continental part).

**Literature data.**Ćukušić (2019); Kučinić et al. (2019c); Neu et al. (2018).

#### ﻿Genus *Mystacides* Berthold, 1827

***Mystacides azureus* (Linnaeus, 1761)**

**Synonyms.***Mystacides atra* Dziedzielewicz, 1877; *Mystacide nigra* Pictet, 1834; *Phryganea nigrocoerulea* Retzius, 1783.

**Distribution in Croatia.** Continental part, Alpine part, Mediterranean part.

**General distribution.** Widely distributed in Europe, including European part of Russia. The species is also recorded in Japan, Russian Far East, and Turkey.

**Ecoregion.**ER5, ER11.

**Drainage.**AS, BS.

**BOLD ID.**CROAA048-18 (Continental part), CROAA127-18 (Continental part), CROTR126-19 (Mediterranean part), CROTR191-19 (Mediterranean part).

**Literature data.**Cerjanec et al. (2020); Ćukušić (2019); Graf et al. (2008); Ivković et al. (2023); Kučinić et al. (2010, 2011, 2017b, 2019c, 2020b); Malicky (2014a); Neu et al. (2018); Previšić et al. (2007a, 2007b, 2010, 2013c); Ridl et al. (2017); Šemnički et al. (2011, 2012); Vučković et al. (2021).

***Mystacides longicornis* (Linnaeus, 1758)**

**Synonyms.***Mystacides concolor* Burmeister, 1839; *Mystacides leucopterus* McLachlan, 1884; *Phryganea minuta* Zetterstedt, 1840; *Mystacides monochrous* McLachlan, 1880; *Phryganea nigrafasciata* Retzius, 1783; *Phryganea quadrifasciata* Fabricius, 1775; *Phryganea sabella* von Paula Schrank, 1802.

**Distribution in Croatia.** Continental part.

**General distribution.** Widely distributed in Europe, including European part of Russia. The species is also recorded in Mongolia and Russian Far East.

**Ecoregion.**ER11.

**Drainage.**BS.

**BOLD ID.**CROAA015-18 (Continental part).

**Literature data.**Ćukušić (2019); Neu et al. (2018); Previšić et al. (2007b).

***Mystacides niger* (Linnaeus, 1758)**

**Synonyms.***Leptocerus nigricans* Stephens, 1836; *Leptocerus obtusus* Stephens, 1836; *Phryganea plumosa* Sulzer, 1776.

**Distribution in Croatia.** Continental part, Alpine part, Mediterranean part.

**General distribution.** Widely distributed in Europe, including European part of Russia. The species is also recorded in Turkey.

**Ecoregion.**ER5, ER11.

**Drainage.**AS, BS.

**BOLD ID.**CROTR079-19 (Mediterranean part), HMKKT861-11 (Continental part).

**Literature data.**Ćukušić (2019); Ćukušić et al. (2017); Ivković et al. (2023); Kučinić et al. (2017b); Malicky (2009); Neu et al. (2018); Previšić et al. (2007b, 2010); Vučković et al. (2021).

#### ﻿Genus *Oecetis* McLachlan, 1877

***Oecetis furva* (Rambur, 1842)**

**Synonyms.***Mystacides filosus* Burmeister, 1839; *Setodes intaminata* McLachlan, 1865.

**Distribution in Croatia.** Continental part, Mediterranean part.

**General distribution.** Widely distributed in Europe, including European part of Russia. The species is also recorded in Iran and Turkey.

**Ecoregion.**ER5, ER11.

**Drainage.**AS, BS.

**BOLD ID.**CROAA043-18 (Continental part), CROTR030-19 (Mediterranean part).

**Literature data.**Ćukušić (2019); Neu et al. (2018); Vrućina et al. (2016).

***Oecetis lacustris* (Pictet, 1834)**

**Synonyms.***Oecetis lacustris orientalis* Martynov, 1935; *Oecetis lacustris martynovi* Yang & Morse, 2000.

**Distribution in Croatia.** Continental part, Alpine part, Mediterranean part.

**General distribution.** Widely distributed in Europe, including European part of Russia.

**Ecoregion.**ER5, ER11.

**Drainage.**AS, BS.

**BOLD ID.**CROTR024-19 (Continental part).

**Literature data.**Ćukušić (2019); Ivković et al. (2023); Kučinić et al. (2017b); Malicky (2014a); Neu et al. (2018); Previšić et al. (2010).

***Oecetis notata* (Rambur, 1842)**

**Synonym.***Oecetis notulata* Navas, 1933.

**Distribution in Croatia.** Continental part, Alpine part, Mediterranean part.

**General distribution.** Widely distributed in Europe, including European part of Russia. The species is also recorded in Turkey.

**Ecoregion.**ER5.

**Drainage.**AS, BS.

**BOLD ID.**CROTR072-19 (Alpine part), CROTR077-19 (Alpine part), CROTR130-19 (Mediterranean part).

**Literature data.**Cerjanec et al. (2020); Ćukušić (2019); Kučinić et al. (2015, 2019a, 2019b, 2020a); Neu et al. (2018); Ridl et al. (2017); Žalac and Kučinić (2022).

***Oecetis ochracea* (Curtis, 1825)**

**Synonyms.***Oecetis albescens* Mosely, 1930; *Oecetis ochracea carri* Milne, 1934; *Phryganea hectica* Zetterstedt, 1840; *Mystacida obsoleta* Rambur, 1842; *Phryganea pilosa* Mueller, 1776; *Oecetis vicaria* Neave, 1934.

**Distribution in Croatia.** Continental part, Alpine part.

**General distribution.** Widely distributed in Europe, including European part of Russia. The species is also recorded in Turkey.

**Ecoregion.**ER5, ER11.

**Drainage.**BS.

**BOLD ID.** No DNA barcoded specimens from Croatia.

**Literature data.**Klapálek (1906); Neu et al. (2018); Previšić et al. (2007b).

***Oecetis testacea* (Curtis, 1834)**

**Distribution in Croatia.** Continental part, Alpine part, Mediterranean part.

**General distribution.** Widely distributed in Europe.

**Ecoregion.**ER5, ER11.

**Drainage.**AS, BS.

**BOLD ID.**CROTR113-19 (Continental part), CROTR115-19 (Continental part), CROTR135-19 (Mediterranean part), CROTR165-19 (Alpine part), CROTR208-19 (Continental part), CROTR209-19 (Continental part).

**Literature data.**Cerjanec et al. (2020); Ćukušić (2019); Graf et al. (2008); Ivković et al. (2023); Kučinić et al. (2008, 2010, 2011, 2017b, 2020a, 2020b); Malicky (2014a); Neu et al. (2018); Previšić et al. (2007a, 2010); Šemnički et al. (2011, 2012); Vučković et al. (2021).

#### ﻿Genus *Setodes* Rambur, 1842

***Setodes punctatus* (Fabricius, 1793)**

**Synonyms.***Setodes hiera* Kolenati, 1858; *Setodes martini* Navas, 1933.

**Distribution in Croatia.** Continental part.

**General distribution.** Widely distributed in Europe, including European part of Russia. Species is also recorded in Armenia, Iran, Morocco, and Turkey.

**Ecoregion.**ER5.

**Drainage.**BS.

**BOLD ID.**CROTR134-19 (Continental part).

**Literature data.**Cerjanec et al. (2020); Ćukušić (2019); Kučinić et al. (2015); Neu et al. (2018).

***Setodes viridis* (Fourcroy, 1785)**

**Synonym.***Setodes punctellus* Rambur, 1842.

**Distribution in Croatia.** Continental part.

**General distribution.** Austria, Belgium, Croatia, France, Germany, Hungary, Italy, Netherlands, North Macedonia, including European part of Russia (on the border with Lithuania). Species is also recorded in Morocco and Turkey.

**Ecoregion.**ER5.

**Drainage.**BS.

**BOLD ID.**CROTR117-19 (Continental part), CROTR137-19 (Continental part).

**Literature data.**Cerjanec et al. (2020); Ćuk and Vučković (2014); Ćukušić (2019); Kučinić et al. (2015).

#### ﻿Genus *Triaenodes* McLachlan, 1865

***Triaenodes lefkas* Malicky, 1974**

**Distribution in Croatia.** Mediterranean part.

**General distribution.** Distribution of the subspecies *Triaenodes ochreellus lefkas* Malicky, 1974 is presented in the Atlas. This subspecies is recorded in Albania, Croatia, Greece, Italy, and Montenegro. Change of taxonomic status (species): Kučinić et al. (2020c).

**Ecoregion.**ER5.

**Drainage.**AS.

**BOLD ID.**CROTR015-19 (Mediterranean part), NIPTR001-17 (Mediterranean part).

**Literature data.**Ćukušić (2019); Kučinić et al. (2020c); Neu et al. (2018); Malicky (2005a); Vučković et al. (2021).

***Triaenodes kawraiskii* Martynov, 1909**

**Synonym.***Triaenodes conspersus banaticus* Botosaneanu, 1958.

**Distribution in Croatia.** Continental part.

**General distribution.** Central Europe, France, Greece, Kyrgyzstan, and Russia.

**Ecoregion.**ER11.

**Drainage.**BS.

**BOLD ID.** No DNA barcoded specimens from Croatia.

**Literature data.**Malicky (2009); Neu et al. (2018).

#### ﻿﻿Family Limnephilidae Kolenati, 1848

**Genus *Allogamus* Schmid, 1955**

***Allogamus auricollis braueri* Kolenati, 1859**

**Distribution in Croatia.** Alpine part.

**General distribution.** Albania, Austria, Belgium, Bosnia and Herzegovina, Czechia, France, Germany, Great Britain, Luxembourg, Montenegro, North Macedonia, Poland, Romania, Slovakia, Slovenia, Spain, Ukraine.

**Ecoregion.**ER5.

**Drainage.**BS.

**BOLD ID.**CROAA040-18 (Alpine part).

**Literature data.**Ćukušić (2019); Kučinić et al. (2020a); Neu et al. (2018); Previšić et al. (2012).

***Allogamus uncatus* (Brauer, 1857)**

**Synonyms.***Hallesus nigricornis* Kolenati, 1848; *Allogamus tomor* Olah, 2012; *Allogamus zugor* Olah, 2015 (2014).

**Distribution in Croatia.** Alpine part, Mediterranean part.

**General distribution.** Albania, Alpine region, Bulgaria, Croatia, Czechia, Germany, Montenegro, North Macedonia, Poland, Romania, Slovakia, Ukraine.

**Ecoregion.**ER5.

**Drainage.**AS, BS.

**BOLD ID.** No DNA barcoded specimens from Croatia.

**Literature data.**Cerjanec et al. (2020); Ivković et al. (2023); Kučinić et al. (2008, 2017b); Malicky (2014a); Neu et al. (2018); Previšić et al. (2007a, 2010, 2014a); Vučković et al. (2011, 2021); Waringer et al. (2009).

#### ﻿Genus *Anabolia* Stephens, 1837

***Anabolia furcata* Brauer, 1857**

**Synonym.***Phryganea laevis* Zetterstedt, 1840.

**Distribution in Croatia.** Continental part, Alpine part.

**General distribution.** Alpine region, Balkan Peninsula, Croatia, Czechia, Denmark, France, Germany, Poland, Romania, Serbia, Slovakia, Ukraine, including European part of Russia.

**Ecoregion.**ER5, ER11.

**Drainage.**BS.

**BOLD ID.**CROAA002-18 (Continental part).

**Literature data.**Cerjanec et al. (2020); Ćukušić (2019); Kučinić et al. (2010, 2020b); Malicky (2009); Neu et al. (2018); Previšić et al. (2007b, 2013c).

#### ﻿Genus *Annitella* Klapálek, 1907

***Annitella apfelbecki* (Klapálek, 1899)**

**Distribution in Croatia.** Continental part, Alpine part, Mediterranean part.

**General distribution.** Albania, Bosnia and Herzegovina, Croatia, Serbia, Slovenia.

**Ecoregion.**ER5.

**Drainage.**AS, BS.

**BOLD ID.**CROTR290-19 (Mediterranean part).

**Literature data.**Cerjanec et al. (2020); Ćukušić (2019); Kučinić et al. (2020a); Marinković-Gospodnetić (1979); Neu et al. (2018); Previšić and Popijač (2010); Previšić et al. (2014a); Vučković et al. (2011, 2021); Waringer et al. (2009).

#### ﻿Genus *Chaetopteryx* Stephens, 1829

***Chaetopteryx bosniaca* Marinković, 1955 (1959?)**

**Synonyms.***Chaetopteryx cissylvanica* Botosaneanu, 1959; *Chaetopteryx frontisdraconis* Botosaneanu, 1993.

**Distribution in Croatia.** Alpine part.

**General distribution.** Balkan Peninsula, Ukraine.

**Ecoregion.**ER5.

**Drainage.**AS.

**BOLD ID.** No DNA barcoded specimens from Croatia.

**Literature data.**Ćukušić (2019); Kučinić et al. (2013, 2019a); Marinković-Gospodnetić (1977, 1979).

***Chaetopteryx bucari* Kučinić, Szivák & Delić, 2013**

**Distribution in Croatia.** Continental part.

**General distribution.** The species is recorded in Croatia, and also in Bosnia and Herzegovina (Oláh et al. 2012).

**Ecoregion.**ER11.

**Drainage.**BS.

**BOLD ID.**HGCAD042-10 (Continental part), HGCAD043-10 (Continental part), HGCAD044-10 (Continental part), HGCAD045-10 (Continental part), HGCAD046-10 (Continental part), HGCAD079-10 (Continental part), HGCAD080-10 (Continental part), HGCAD081-10 (Continental part), HGCAD082-10 (Continental part), HGCAD083-10 (Continental part), HGCAD084-10 (Continental part), HGCAD085-10 (Continental part), HGCAD086-10 (Continental part), HGCAD087-10 (Continental part).

**Literature data.**Ćukušić (2019); Kučinić et al. (2013, 2020b); Malicky (2014b); Neu et al. (2018); Oláh et al. (2012); Szivák et al. (2017).

***Chaetopteryx fusca* Brauer, 1857**

**Distribution in Croatia.** Continental part, Alpine part, Mediterranean part.

**General distribution.** Croatia, Czechia, Eastern Alps, Hungary, Poland, Slovakia.

**Ecoregion.**ER5, ER11.

**Drainage.**AS, BS.

**BOLD ID. BOLD ID.**CROAA081-18 (Mediterranean part), CROAA082-18 (Alpine part).

**Literature data.**Cerjanec et al. (2020); Ćukušić (2019); Ivković et al. (2023); Kučinić et al. (2013, 2017b); Malicky (2014a); Neu et al. (2018); Previšić and Popijač (2010); Previšić et al. (2007a, 2007b, 2010, 2014a); Šemnički et al. (2011, 2012); Vučković et al. (2011, 2021); Waringer et al. (2009).

***Chaetopteryx gonospina* Marinković-Gospodnetić, 1966**

**Distribution in Croatia.** Continental part, Alpine part.

**General distribution.** Bosnia and Herzegovina, border between France and Spain.

**Ecoregion.**ER5, ER11.

**Drainage.**BS.

**BOLD ID.**HGCAD052-10 (Continental part), HGCAD053-10 (Continental part).

**Literature data.**Cerjanec et al. (2020); Ivković et al. (2023); Kučinić et al. (2010, 2013; 2017b, 2020b); Oláh et al. (2022b); Previšić et al. (2007a, 2010); Žalac and Kučinić (2022).

***Chaetopteryx major* McLachlan, 1876**

**Synonym.***Chaetopteryx irregularis* Kolenati, 1859.

**Distribution in Croatia.** Continental part, Alpine part.

**General distribution.** Alpine region, Belgium, Croatia, Czechia, Hungary, Luxembourg, Slovakia.

**Ecoregion.**ER5, ER11.

**Drainage.**BS.

**BOLD ID.**CROAA103-18 (Continental part), CROTR399-22 (Continental part).

**Literature data.**Ćukušić (2019); Klapálek (1906); Kučinić et al. (2010, 2013, 2020b); Neu et al. (2018); Previšić and Popijač (2010); Previšić et al. (2013a); Radovanović (1935).

***Chaetopteryx marinkovicae* Malicky & Krušnik, 1988**

**Synonym.***Chaetopteryx malickyi* Valle, 2001.

**Distribution in Croatia.** Mediterranean part.

**General distribution.** The species is recorded in Croatia, and also in Slovenia (Oláh et al. 2012).

**Ecoregion.**ER5.

**Drainage.**AS.

**BOLD ID.**HGCAD022-10 (Mediterranean part), HGCAD023-10 (Mediterranean part), HGCAD024-10 (Mediterranean part), HGCAD025-10 (Mediterranean part), HGCAD026-10 (Mediterranean part).

**Literature data.**Ćukušić (2019); Kučinić et al. (2013); Malicky (2014b); Malicky and Krušnik (1988); Neu et al. (2018); Oláh et al. (2012); Szivák et al. (2017).

***Chaetopteryx rugulosa* Kolenati, 1848**

**Synonyms.***Chaetopteryx prealpensis* Oláh, 2012; *Chaetopteryx zalaensis* Oláh, 2012 - according to Malicky 2014b.

**Distribution in Croatia.** Continental part.

**General distribution.** Austria, Croatia, Hungary, Slovenia.

**Ecoregion.**ER5, ER11.

**Drainage.**BS.

**BOLD ID.**CROAA003-18 (Continental part), CROAA077-18 (Continental part), CROAA122-18 (Continental part), CROTR111-19 (Continental part), CROTR248-19 (Continental part), HGCAD035-10 (Continental part), HGCAD068-10 (Continental part), HGCAD069-10 (Continental part), HGCAD070-10 (Continental part), HGCAD071-10 (Continental part), NIPAG002-17 (Continental part).

**Literature data.**Ćukušić (2019); Klapálek (1906); Kučinić et al. (2010, 2013); Malicky (1996, 2014b); Neu et al. (2018); Oláh et al. (2012, 2015); Radovanović (1935); Szivák et al. (2017).

***Chaetopteryx schmidi* Botosaneanu, 1957**

**Synonym.***Chaetopteryx papukensis* Oláh & Szivák, 2012 - according Malicky 2014b.

**Distribution in Croatia.** Continental part.

**General distribution.** Bosnia and Herzegovina, Croatia, Romania (Neu et al. 2018), and also Serbia (Malicky 2014b).

**Ecoregion.**ER11.

**Drainage.**BS.

**BOLD ID.**HGCAD029-10 (Continental part), HGCAD054-10 (Continental part), HGCAD055-10 (Continental part), HGCAD056-10 (Continental part).

**Literature data.**Ćukušić (2019); Kučinić et al. (2010, 2013); Malicky (2014b); Neu et al. (2018); Oláh (2010); Oláh et al. (2012, 2015); Previšić et al. (2013c).

***Chaetopteryx uherkovichi* Oláh, 2011**

**Distribution in Croatia.** Continental part.

**General distribution.** The species is recorded only in Croatia.

**Ecoregion.**ER11.

**Drainage.**BS.

**BOLD ID.** No DNA barcoded specimens from Croatia.

**Literature data.**Kučinić et al. (2013); Neu et al. (2018); Oláh (2011).

#### ﻿Genus *Colpotaulius* Kolenati, 1848

***Colpotaulius incisus* (Curtis, 1834)**

**Synonyms.***Colpotaulius excisus* Kolenati, 1848; *Limnephila striolata* Rambur, 1842.

**Distribution in Croatia.** Continental part, Mediterranean part.

**General distribution.** Apennine Peninsula, France, Scandinavia.

**Ecoregion.**ER5, ER11.

**Drainage.**AS, BS.

**BOLD ID.** No DNA barcoded specimens from Croatia.

**Literature data.**Ćukušić (2019); Kučinić et al. (2011); Neu et al. (2018); Vučković et al. (2021).

#### ﻿Genus *Drusus* Stephens, 1837

***Drusus chrysotus* (Rambur, 1842)**

**Distribution in Croatia.** Alpine part.

**General distribution.** Alpine region, Croatia, Czechia, France.

**Ecoregion.**ER5.

**Drainage.**BS.

**BOLD ID.**CROAA104-18 (Alpine part).

**Literature data.**Cerjanec et al. (2020); Ćukušić (2019); Kladarić et al. (2021); Kučinić et al. (2014); Neu et al. (2018); Previšić et al. (2012, 2014b).

***Drusus croaticus* Marinković-Gospodnetić, 1971**

**Distribution in Croatia.** Alpine part.

**General distribution.** Croatia, Slovenia.

**Ecoregion.**ER5.

**Drainage.**AS, BS.

**BOLD ID.**CROAA041-18 (Alpine part), CROAA083-18 (Alpine part), CROTR017-19 (Alpine part), CROTR019-19 (Alpine part), CROTR043-19 (Alpine part).

**Literature data.**Cerjanec et al. (2020); Ćukušić (2019); Ivković et al. (2013, 2023); Kladarić et al. (2021); Kučinić et al. (2008, 2014, 2017b, 2019a, 2020a); Malicky (2014a); Marinković-Gospodnetić (1971, 1977, 1979); Neu et al. (2018); Oláh et al. (2017); Previšić (2009); Previšić and Popijač (2010); Previšić et al. (2007a, 2009, 2010, 2012, 2014b); Vučković et al. (2011); Žalac and Kučinić (2022).

***Drusus discolor* (Rambur, 1842)**

**Synonyms.***Drusus bicolor* Navas, 1918; *Drusus tricolor* Obenberger, 1952.

**Distribution in Croatia.** Alpine part.

**General distribution.** Alpine region, Balkan Peninsula, Croatia, Czechia, France, Germany, Poland, Slovakia, Spain, Ukraine.

**Ecoregion.**ER5.

**Drainage.**BS.

**BOLD ID.**CROTR020-19 (Alpine part).

**Literature data.**Cerjanec et al. (2020); Ćukušić (2019); Kladarić et al. (2021); Kučinić et al. (2014, 2020a); Neu et al. (2018); Previšić and Popijač (2010); Previšić et al. (2012, 2014b).

***Drusus schmidi* Botosaneanu, 1960**

**Distribution in Croatia.** Continental part, Alpine part.

**General distribution.** Balkna Peninsula.

**Ecoregion.**ER5, ER11.

**Drainage.**BS.

**BOLD ID.**CROAA021-18 (Alpine part), CROTR189-19 (Continental part), CROTR333-21 (Continental part), CROTR334-21 (Continental part), CROTR335-21 (Continental part), CROTR336-21 (Continental part), CROTR337-21 (Continental part).

**Literature data.**Ćukušić (2019); Kladarić et al. (2021); Kučinić et al. (2014, 2020a); Neu et al. (2018); Previšić et al. (2013c).

***Drusus vespertinus* Marinković-Gospodnetić, 1976**

**Distribution in Croatia.** Alpine part.

**General distribution.** Croatia, Bosnia and Herzegovina.

**Ecoregion.**ER5.

**Drainage.**BS.

**BOLD ID.**CROTR275-19 (Alpine part).

**Literature data.**Ćukušić (2019); Kladarić et al. (2021); Kučinić et al. (2014, 2020a); Neu et al. (2018); Previšić et al. (2012, 2014b).

#### ﻿Genus *Ecclisopteryx* Kolenati, 1848

***Ecclisopteryx asterix* Malicky, 1979**

**Distribution in Croatia.** Continental part.

**General distribution.** Southeastern Alps.

**Ecoregion.**ER11.

**Drainage.**BS.

**BOLD ID.**CROTR339-21 (Continental part), CROTR340-21 (Continental part), CROTR341-21 (Continental part).

**Literature data.**Kladarić et al. (2021).

***Ecclisopteryx ivkae* Previšić, Graf & Vitecek, 2014**

**Distribution in Croatia.** Mediterranean part.

**General distribution.** The species is recorded only in Croatia.

**Ecoregion.**ER5.

**Drainage.**AS.

**BOLD ID.**CROAA106-18 (Mediterranean part).

**Literature data.**Ćukušić (2019); Kladarić et al. (2021); Kučinić et al. (2020a); Neu et al. (2018); Oláh et al. (2017); Previšić et al. (2014a); Vučković et al. (2011, 2016, 2021); Waringer et al. (2009).

***Ecclisopteryx keroveci* Previšić, Graf & Vitecek, 2014**

**Distribution in Croatia.** Continental part, Alpine part.

**General distribution.** Widely distributed in Balkan Peninsula, Slovenia.

**Ecoregion.**ER5, ER11.

**Drainage.**AS, BS.

**BOLD ID.**CROAA007-18 (Alpine part), CROAA059-18 (Continental part), CROTR041-19 (Alpine part), CROTR338-21 (Alpine part).

**Literature data.**Ćukušić (2019); Kladarić et al. (2021); Neu et al. (2018); Previšić and Popijač (2010); Previšić et al. (2012, 2014a); Vučković et al. (2011, 2016).

#### ﻿Genus *Glyphotaelius* Stephens, 1833

***Glyphotaelius pellucidus* (Retzius, 1783)**

**Synonyms.***Phryganea argentata* Gmelin, 1789; *Limnephilus basalis* Curtis, 1834; *Limnephilus emarginatus* Curtis, 1834.

**Distribution in Croatia.** Continental part, Alpine part, Mediterranean part.

**General distribution.** Widely distributed in Europe, including European part of Russia. The species is also recorded in Turkey.

**Ecoregion.**ER5, ER11.

**Drainage.**AS, BS.

**BOLD ID.**CROTR005-19 (Alpine part), CROTR027-19 (Continental part), CROTR064-19 (Mediterranean part), CROTR069-19 (Alpine part), CROTR227-19 (Continental part).

**Literature data.**Cerjanec et al. (2020); Ćukušić (2019); Ivković et al. (2023); Klapálek (1906); Kučinić et al. (2017b, 2020a, 2020b); Malicky (2009, 2014a); Neu et al. (2018); Previšić et al. (2010, 2013c); Šemnički et al. (2011, 2012); Vučković et al. (2021); Žalac and Kučinić (2022).

#### ﻿Genus *Grammotaulius* Kolenati, 1848

***Grammotaulius nigropunctatus* (Retzius, 1783)**

**Synonyms.***Grammotaulius atomarius* (Fabricius, 1793); *Phryganea irroratus* Zetterstedt, 1840; *Phryganea lineola* von Paula Schrank, 1781; *Grammotaulius suarezi* Navas, 1916.

**Distribution in Croatia.** Continental part, Alpine part, Mediterranean part.

**General distribution.** Widely distributed in Europe, including European part of Russia. The species is also recorded in Turkey.

**Ecoregion.**ER5, ER11.

**Drainage.**AS, BS.

**BOLD ID.**CROTR004-19 (Alpine part).

**Literature data.**Cerjanec et al. (2020); Ćukušić (2019); Ivković et al. (2023); Klapálek (1906); Kučinić et al. (2011, 2017b); Malicky (2009, 2014a); Neu et al. (2018); Previšić et al. (2007a, 2010, 2014a); Radovanović (1935); Vučković et al. (2011, 2021); Waringer et al. (2009); Žalac and Kučinić (2022).

#### ﻿Genus *Halesus* Stephens, 1836

***Halesus digitatus* (von Paula Schrank, 1781)**

**Synonyms.***Phryganea collaris* von Paula Schrank, 1781; *Halesus hammoniensus* Ulmer, 1902; *Limnephilus hieroglyphicus* Curtis, 1834.

**Distribution in Croatia.** Continental part, Alpine part, Mediterranean part.

**General distribution.** Widely distributed in Europe, including European part of Russia. The subspecies *Halesus digitatus caucasicus* Olah, 1985 is recognised in Turkey and Iran.

**Ecoregion.**ER5, ER11.

**Drainage.**AS, BS.

**BOLD ID.**CROAA101-18 (Continental part), CROTR035-19 (Alpine part), CROTR038-19 (Continental part), CROTR042-19 (Continental part), CROTR066-19 (Continental part), CROTR221-19 (Alpine part), CROTR277-19 (Continental part), NIPM009-17 (Mediterranean part).

**Literature data.**Cerjanec et al. (2020); Ćukušić (2019); Graf et al. (2008); Ivković et al. (2023); Kučinić et al. (2011, 2017b, 2020a, 2021); Malicky (2014a); Neu et al. (2018); Previšić et al. (2007a, 2010, 2013c, 2014a); Ridl et al. (2017); Šemnički et al. (2011, 2012); Vučković et al. (2011, 2021); Waringer et al. (2009); Žalac and Kučinić (2022).

***Halesus tesselatus* (Rambur, 1842)**

**Distribution in Croatia.** Continental part, Alpine part, Mediterranean part.

**General distribution.** Widely distributed in Europe, including European part of Russia.

**Ecoregion.**ER5, ER11.

**Drainage.**AS, BS.

**BOLD ID.**CROAA084-18 (Continental part).

**Literature data.**Cerjanec et al. (2020); Ćukušić (2019); Ivković et al. (2023); Klapálek (1906); Kučinić et al. (2008, 2010, 2011, 2017b, 2020b); Malicky (2014a); Neu et al. (2018); Previšić et al. (2007a, 2010); Šemnički et al. (2011, 2012); Vučković et al. (2021).

#### ﻿Genus *Hydatophylax* Wallengren, 1891

***Hydatophylax infumatus* (McLachlan, 1865)**

**Distribution in Croatia.** Alpine part, Mediterranean part.

**General distribution.** Alpine region, Belgium, Bosnia and Herzegovina, British Islands, Czechia, Estonia, France, Germany, Lithuania, Luxembourg, Poland, Romania, Scandinavian Peninsula, Slovakia, Ukraine, including European part of Russia (along the Ural Mountains).

**Ecoregion.**ER5.

**Drainage.**AS, BS.

**BOLD ID.**HMKKT037-10 (Alpine part).

**Literature data.**Ivković et al. (2023); Kučinić et al. (2017b); Malicky (2014a); Previšić et al. (2010); Vučković et al. (2021).

#### ﻿Genus *Ironoquia* Banks, 1916

***Ironoquia dubia* (Stephens, 1837)**

**Distribution in Croatia.** Continental part.

**General distribution.** Belarus, Belgium, Croatia, Czechia, France, Germany, Great Britain, Latvia, Lithuania, Netherlands, Poland, Romania, Scandinavian Peninsula, southeastern Alps, Slovakia, Ukraine.

**Ecoregion.**ER11.

**Drainage.**BS.

**BOLD ID.**CROAA022-18 (Continental part), CROAA096-18 (Continental part).

**Literature data.**Ćuk and Vučković (2010); Ćukušić (2019); Klapálek (1906); Neu et al. (2018).

#### ﻿Genus *Limnephilus* Leach, 1815

***Limnephilus affinis* Curtis, 1834**

**Synonyms.***Goniotaulius anastomosis* Kolenati, 1848; *Chaetotaulius angustatus* Kolenati, 1848; *Limnephilus costalis* Stephens, 1852; *Goniotaulius nigrovittatus* Kolenati, 1859; *Limnephilus paonius* Denning, 1949.

**Distribution in Croatia.** Continental part, Alpine part, Mediterranean part.

**General distribution.** Widely distributed in Europe, including European part of Russia. The species is also recorded in Iran and Turkey.

**Ecoregion.**ER5, ER11.

**Drainage.**AS, BS.

**BOLD ID.**CROAA042-18 (Continental part), CROTR032-19 (Mediterranean part).

**Literature data.**Ćukušić (2019); Ivković et al. (2023); Klapálek (1906); Kučinić et al. (2011, 2017b, 2019c); Neu et al. (2018); Previšić et al. (2010); Radovanović (1935); Vučković et al. (2021).

***Limnephilus auricula* Curtis, 1834**

**Synonyms.***Limnephilus geminus* Stephens, 1837; *Limnephila guttata* Rambur, 1842; *Phryganea nigridorsa* Pictet, 1834; *Limnephilus obscurus* Curtis, 1834; *Limnephilus signatus* Stephens, 1837, in part.

**Distribution in Croatia.** Continental part, Alpine part, Mediterranean part.

**General distribution.** Widely distributed in Europe, including European part of Russia. The species is also recorded in Turkey.

**Ecoregion.**ER5, ER11.

**Drainage.**AS, BS.

**BOLD ID.**CROAA023-18 (Alpine part).

**Literature data.**Cerjanec et al. (2020); Ćukušić (2019); Ivković et al. (2023); Klapálek (1906); Kučinić et al. (2008, 2017b); Malicky (2009, 2014a); Marinković-Gospodnetić (1979); Neu et al. (2018); Previšić et al. (2010); Radovanović (1935); Vučković et al. (2021); Žalac and Kučinić (2022).

***Limnephilus bipunctatus* Curtis, 1834**

**Synonyms.***Limnophilus barbatus* Martynov, 1917 (1916); *Lasiocephalus basalis* Hoeppner & le Roi, 1912 (1911); *Limnophilus diacanthus* Navas, 1917; *Phryganea discoidea* Zetterstedt, 1840; *Limnephila obscura* Rambur, 1842; *Limnophilus tuberculatus* Brauer, 1857.

**Distribution in Croatia.** Continental part, Alpine part, Mediterranean part.

**General distribution.** Widely distributed in Europe, including European part of Russia (along the Ural Mountains). The species is also recorded in Turkey.

**Ecoregion.**ER5, ER11.

**Drainage.**AS, BS.

**BOLD ID.**CROTR147-19 (Continental part).

**Literature data.**Ćukušić (2019); Klapálek (1906); Malicky (2009); Neu et al. (2018); Vučković et al. (2021).

***Limnephilus decipiens* (Kolenati, 1848)**

**Synonyms.***Phryganea bimaculata* Scopoli not Linnaeus, 1763; *Chaetotaulius nobilis* Brauer not Kolenati, 1855.

**Distribution in Croatia.** Continental part.

**General distribution.** Widely distributed in Europe, including European part of Russia. The species is also recorded in Turkey.

**Ecoregion.**ER11.

**Drainage.**BS.

**BOLD ID.** No DNA barcoded specimen from Croatia.

**Literature data.**Malicky (2009); Neu et al. (2018).

***Limnephilus extricatus* McLachlan, 1865**

**Distribution in Croatia.** Continental part, Alpine part.

**General distribution.** Widely distributed in Europe, including European part of Russia. The species is also recorded in Turkey.

**Ecoregion.**ER5, ER11.

**Drainage.**BS.

**BOLD ID.**CROTR003-19 (Alpine part).

**Literature data.**Ćukušić (2019); Ivković et al. (2023); Kučinić et al. (2017b); Malicky (2014a); Neu et al. (2018); Previšić et al. (2010); Žalac and Kučinić (2022).

***Limnephilus flavicornis* (Fabricius, 1787)**

**Synonyms.***Limnophilus apicalis* Martynov, 1924; *Limnephilus dorsalis* Stephens, 1837; *Phryganea fusca* Rothschild, 1878; *Limnephilus sibiricus occidentis* Spuris, 1988; *Phryganea testacea* Costa, 1847; *Phryganea viridiventris* Dufour, 1841.

**Distribution in Croatia.** Continental part, Alpine part, Mediterranean part.

**General distribution.** Widely distributed in Europe, including European part of Russia. The species is also recorded in Turkey.

**Ecoregion.**ER5, ER11.

**Drainage.**AS, BS.

**BOLD ID.**CROAA008-18 (Continental part), CROAA009-18 (Continental part), CROTR073-19 (Alpine part), CROTR391-22 (Continental part).

**Literature data.**Ćukušić (2019); Ivković et al. (2023); Klapálek (1906); Kučinić et al. (2017b, 2019a, 2020a); Malicky (2009); Neu et al. (2018); Previšić et al. (2010, 2014a); Vučković et al. (2011, 2021); Waringer et al. (2009); Žalac and Kučinić (2022).

***Limnephilus graecus* Schmid, 1965**

**Distribution in Croatia.** Mediterranean part.

**General distribution.** Albania, Croatia, Greece, Montenegro.

**Ecoregion.**ER5.

**Drainage.**AS.

**BOLD ID.**CROAA047-18 (Mediterranean part), CROTR278-19 (Mediterranean part).

**Literature data.**Ćukušić (2019); Kučinić et al. (2015); Neu et al. (2018).

***Limnephilus griseus* (Linneaus, 1758)**

**Synonyms.***Limnephilus bipunctatus* Stephens, 1837; *Limnephilus fenestralis* Curtis, 1834; *Limnephilus luniger* Stephens, 1837; *Limnephilus marginalis* Stephens, 1837; *Limnephilus obliquus* Stephens, 1837; *Limnephilus signatus* Stephens, 1837; *Limnephila variegata* Rambur, 1842.

**Distribution in Croatia.** Continental part, Alpine part, Mediterranean part.

**General distribution.** Widely distributed in Europe, including European part of Russia. The species is also recorded in Turkey.

**Ecoregion.**ER5, ER11.

**Drainage.**AS, BS.

**BOLD ID.**CROAA125-18 (Continental part).

**Literature data.**Ćukušić (2019); Klapálek (1906); Kučinić et al. (2020b); Malicky (2009); Neu et al. (2018); Vučković et al. (2011, 2021).

***Limnephilus hirsutus* (Pictet, 1834)**

**Distribution in Croatia.** Continental part, Alpine part, Mediterranean part.

**General distribution.** Widely distributed in Europe, including Turkey.

**Ecoregion.**ER5, ER11.

**Drainage.**AS, BS.

**BOLD ID.**CROTR029-19 (Alpine part), CROTR094-19 (Continental part), NIPM010-18 (Mediterranean part).

**Literature data.**Cerjanec et al. (2020); Ćukušić (2019); Ivković et al. (2023); Kučinić et al. (2008, 2011, 2017b; 2020a); Malicky (2014a); Neu et al. (2018); Previšić et al. (2010); Vučković et al. (2021); Žalac and Kučinić (2022).

***Limnephilus ignavus* McLachlan, 1865**

**Synonym.***Halesus rivularis* Navas, 1917.

**Distribution in Croatia.** Continental part, Alpine part.

**General distribution.** Widely distributed in Europe, including European part of Russia.

**Ecoregion.**ER5, ER11.

**Drainage.**BS.

**BOLD ID.**CROTR040-19 (Alpine part), CROTR279-19 (Alpine part).

**Literature data.**Ćukušić (2019); Ivković et al. (2023); Klapálek (1906); Kučinić et al. (2008, 2017b, 2020a); Malicky (2014a); Neu et al. (2018); Previšić et al. (2010); Žalac and Kučinić (2022).

***Limnephilus lunatus* Curtis, 1834**

**Synonyms.***Limnephilus apicalis* Curtis, 1834; *Limnephila flavida* Rambur, 1842; *Phryganea lunaris* Pictet, 1834; *Limnephilus nebulosus* Curtis, 1834.

**Distribution in Croatia.** Continental part, Alpine part, Mediterranean part.

**General distribution.** Widely distributed in Europe, including European part of Russia. The species is also recorded in Iran, Lebanon, Morocco, and Turkey.

**Ecoregion.**ER5, ER11.

**Drainage.**AS, BS.

**BOLD ID.**CROTR009-19 (Alpine part), CROTR071-19 (Alpine part), CROTR233-19 (Mediterranean part).

**Literature data.**Cerjanec et al. (2020); Ćukušić (2019); Graf et al. (2008); Ivković et al. (2013, 2023); Kučinić et al. (2011, 2017b, 2020a, 2021); Malicky (2009, 2014a); Neu et al. (2018); Previšić et al. (2007a, 2007b, 2010, 2013c, 2014a); Ridl et al. (2017); Šemnički et al. (2011, 2012); Vučković et al. (2011, 2021); Waringer et al. (2009); Žalac and Kučinić (2022).

***Limnephilus marmoratus* Curtis, 1834**

**Synonyms.***Phryganea atomaria* Zetterstedt, 1840; *Limnephilus discoidalis* Curtis, 1834; *Phryganea maculata* Costa, 1847; Limnephilus (Chaetotaulius) vitratus Walker, 1852; *Limnephila vitrea* Rambur, 1842.

**Distribution in Croatia.** Mediterranean part.

**General distribution.** Alpine region, Benelux, Bosnia and Herzegovina, British Islands, Croatia, France, Germany, Greece, Iberian Peninsula, Lithuania, Montenegro, Poland, Romania, Scandinavian Peninsula, Ukraine.

**Ecoregion.**ER5.

**Drainage.**AS.

**BOLD ID.**CROAA072-18 (Mediterranean part), CROTR096-19 (Mediterranean part), CROTR097-19 (Mediterranean part), CROTR276-19 (Mediterranean part).

**Literature data.**Ćukušić (2019); Klapálek (1906); Kučinić et al. (2011, 2019b, 2019c, 2020a); Neu et al. (2018); Vučković et al. (2011, 2021).

***Limnephilus rhombicus* (Linnaeus, 1758)**

**Synonyms.***Limnephilus chilcotinensis* Nimmo, 1991; *Limnephilus combinatus* Walker, 1852; *Phryganea rhomboidica* Berkenhout, 1795.

**Distribution in Croatia.** Continental part, Alpine part, Mediterranean part.

**General distribution.** Widely distributed in Europe, including European part of Russia. The species is also recorded in Mongolia, North America, Russian Far East, and Turkey.

**Ecoregion.**ER5, ER11.

**Drainage.**AS, BS.

**BOLD ID.**CROAA097-18 (Alpine part), CROTR023-19 (Alpine part), CROTR188-19 (Alpine part), CROTR197-19 (Alpine part).

**Literature data.**Cerjanec et al. (2020); Ćukušić (2019); Graf et al. (2008); Ivković et al. (2013, 2023); Klapálek (1906); Kučinić et al. (2008, 2011, 2017b, 2019a, 2020a, 2020b); Malicky (2014a); Neu et al. (2018); Previšić et al. (2007a, 2007b, 2010, 2013c); Šemnički et al. (2011, 2012); Vučković et al. (2021); Žalac and Kučinić (2022).

***Limnephilus sparsus* Curtis, 1834**

**Synonyms.***Limnephilus flavescens* Stephens, 1837; *Limnephilus fuscatus* Stephens, 1837; *Limnephilus fuscus* Stephens, 1837; *Limnephilus cianficconiae hispaniae* Botosaneanu, 2004; *Desmotaulius megerlei* Kolenati, 1848; Limnophilus*paramushirensis* Tsuda, 1942; *Limnephilus punctatissimus* Stephens, 1837; *Limnephilus tenebricus* Curtis, 1834; *Desmotaulius unimaculatus* Kolenati, 1848; *Limnephilus vinculum* Curtis, 1834.

**Distribution in Croatia.** Continental part, Alpine part, Mediterranean part.

**General distribution.** Widely distributed in Europe, including European part of Russia. The species is also recorded in Turkey.

**Ecoregion.**ER5, ER11.

**Drainage.**AS, BS.

**BOLD ID.**CROTR001-19 (Alpine part), CROTR025-19 (Alpine part).

**Literature data.**Ćukušić (2019); Ivković et al. (2023); Kučinić et al. (2008, 2017b, 2020a); Malicky (2009, 2014a); Neu et al. (2018); Previšić et al. (2010); Vučković et al. (2011, 2021); Žalac and Kučinić (2022).

***Limnephilus vittatus* (Fabricius, 1798)**

**Synonyms.***Limnephilus consobrinus* Curtis, 1834; *Phryganea elegans* Pictet, 1834; *Limnephilus nigrivittatus* Stephens, 1837; *Limnephilus notatus* Stephens, 1837; *Limnephilus praeustus* Stephens, 1837; *Phryganea subpunctulatus* Zetterstedt, 1840; *Limnephilus substrigosus* Stephens, 1837.

**Distribution in Croatia.** Continental part, Alpine part, Mediterranean part.

**General distribution.** Widely distributed in Europe, including European part of Russia. The species is also recorded in Turkey.

**Ecoregion.**ER5, ER11

**Drainage.**AS, BS.

**BOLD ID.**CROTR006-19 (Alpine part).

**Literature data.**Ćukušić (2019); Klapálek (1906); Kučinić et al. (2020a); Malicky (2009, 2014a); Neu et al. (2018); Previšić et al. (2014a); Vučković et al. (2011, 2021); Waringer et al. (2009); Žalac and Kučinić (2022).

#### ﻿Genus *Mesophylax* McLachlan, 1882

***Mesophylax aspersus* (Rambur, 1842)**

**Synonym.***Stenophylax meridionalis* Kolenati, 1848.

**Distribution in Croatia.** Mediterranean part.

**General distribution.** Alpine region, Apennine Peninsula including Sardinia and Sicily, Croatia, Cyprus, France with Corsica, Great Britain, Greek Islands, Spain with Balearic Islands. The species is also recorded in Algeria, Egypt, Lebanon, Morocco, Syria, and Tunisia.

**Ecoregion.**ER5.

**Drainage.**AS.

**BOLD ID.**CROTR083-19 (Mediterranean part), CROTR281-19 (Mediterranean part).

**Literature data.**Ćukušić (2019); Kučinić et al. (2019c, 2020a); Malicky (2005a); Neu et al. (2018); Ridl et al. (2017); Žalac and Kučinić (2022).

***Mesophylax impunctatus* McLachlan, 1884**

**Distribution in Croatia.** Mediterranean part.

**General distribution.** Three subspecies are recognised, each with a different distribution. *Mesophylax impunctatus impunctatus* McLachlan, 1884 is distributed in the Alpine region; *Mesophylax impunctatus aduncus* Navás, 1923 occurs in the Balkan Peninsula and Turkey, while *Mesophylax impunctatus zetlandicus* McLachlan, 1884 is found in the British Isles. In addition, a *Mesophylax impunctatus* species complex has been recognised in Spain. Most likely in Croatia - subspecies *M. impunctatus aduncus*.

**Ecoregion.**ER5.

**Drainage.**AS.

**BOLD ID.** No DNA barcoded specimens from Croatia.

**Literature data.**Vučković et al. (2011, 2021).

#### ﻿Genus *Parachiona* Thomson, 1891

***Parachiona picicornis* (Pictet, 1834)**

**Synonyms.***Limnephila nigrita* Rambur, 1842; *Phryganea puberula* Zettersted, 1840.

**Distribution in Croatia.** Alpine part.

**General distribution.** Central and north Europe including Belarus, Estonia, France, Norway, Ukraine, and European part of Russia.

**Ecoregion.**ER5.

**Drainage.**BS.

**BOLD ID.** No DNA barcoded specimens from Croatia.

**Literature data.**Klapálek (1906); Neu et al. (2018).

#### ﻿Genus *Platyphylax* McLachlan, 1871

***Platyphylax frauenfeldi* (Brauer, 1857)**

**Synonyms.***Enoicyla clara* Hagen, 1864; *Platyphylax kolenatii* Kolenati, 1859; *Platyphylax pallescens* McLachlan, 1875.

**Distribution in Croatia.** Continental part.

**General distribution.** Austria, Croatia, Hungary, Slovenia, Switzerland.

**Ecoregion.**ER11.

**Drainage.**BS.

**BOLD ID.** No DNA barcoded specimens from Croatia.

**Literature data.**Malicky (2009, 2024); Malicky et al. (2002); Neu et al. (2018).

#### ﻿Genus *Potamophylax* Wallengren, 1891

***Potamophylax cingulatus cingulatus* (Stephens, 1837)**

**Distribution in Croatia.** Alpine part.

**General distribution.** Belarus, Benelux, British Islands, Croatia, Czechia, France, Germany, Iceland, Iberian Peninsula, Poland, Scandinavian Peninsula, Slovakia, including the European part of Russia (northern region of Ural Mountains).

**Ecoregion.**ER5.

**Drainage.**BS.

**BOLD ID.** No DNA barcoded specimens from Croatia.

**Literature data.**Neu et al. (2018).

***Potamophylax cingulatus alpinus* Tobias, 1994**

**Distribution in Croatia.** Continental part.

**General distribution.** Alpine region.

**Ecoregion.**ER11.

**Drainage.**BS.

**BOLD ID.**CROAA087-18 (Continental part).

**Literature data.**Ćukušić (2019). According to the Trichoptera World Checklist (2025) this subspecies has the species level, *Potamophylax alpinus* Tobias, 1994.

***Potamophylax cingulatus depilis* Szczęsny, 1994**

**Distribution in Croatia.** Alpine part.

**General distribution.** Poland, Slovakia, Ukraine.

**Ecoregion.**ER5.

**Drainage.**AS.

**BOLD ID.** No DNA barcoded specimens from Croatia.

**Literature data.**Oláh et al. (2018). According to the Trichoptera World Checklist (2025) this subspecies has the species level *Potamophylax depilis* Szczęsny, 1994.

***Potamophylax latipennis* (Curtis, 1834)**

**Synonyms.***Phryganea fasciata* Gmelin, 1789; *Phryganea guttifera* Zetterstedt, 1840; *Phryganea laciniosa* Gmelin, 1789; *Phryganea pantherina* Pictet, 1834; *Limnephilus stellatus* Curtis, 1834.

**Distribution in Croatia.** Continental part, Alpine part, Mediterranean part.

**General distribution.** Widely distributed in Europe, including European part of Russia (along the Ural Mountains). The species is also recorded in Turkey.

**Ecoregion.**ER5, ER11.

**Drainage.**AS, BS.

**BOLD ID.**CROAA027-18 (Alpine part), CROAA028-18 (Alpine part), CROAA078-18 (Mediterranean part), CROAA079-18 (Alpine part), CROTR207-19 (Continental part).

**Literature data.**Cerjanec et al. (2020); Ćukušić (2019); Ivković et al. (2023); Kučinić et al. (2017b, 2019a); Malicky (2014a); Marinković-Gospodnetić (1979); Neu et al. (2018); Previšić et al. (2007a, 2010, 2013c); Šemnički et al. (2011, 2012); Žalac and Kučinić (2022).

***Potamophylax luctuosus* (Piller & Mitterpacher, 1783)**

**Synonyms.**Stenophylax pantherinus var. brittingeriana Kolenati, 1848; *Stenophylax excisus* Martynov, 1926; Stenophylax pantherinus var. geometrina Kolenati, 1848; *Stenophylax luctuosus* Piller & Mitterpacher, 1783; *Anabolia gigantea* Brauer, 1857.

**Distribution in Croatia.** Continental part, Alpine part.

**General distribution.** Alpine region, Balkan Peninsula, Belgium, Croatia, Czechia, Denmark, Estonia, France, Germany, Hungary, Lithuania, Luxembourg, Poland, Slovakia, Ukraine.

**Ecoregion.**ER5, ER11.

**Drainage.**BS.

**BOLD ID.**CROAA113-18 (Continental part), CROTR282-19 (Continental part).

**Literature data.**Ćukušić (2019); Ivković et al. (2023); Kučinić et al. (2017b, 2020b); Malicky (2014a); Neu et al. (2018); Previšić and Popijač (2010); Šemnički et al. (2011, 2012).

***Potamophylax nigricornis* (Pictet, 1834)**

**Synonyms.***Potamophylax apados* Oláh & Chvojka, 2013; *Stenophylax areatus* Kolenati, 1856.

**Distribution in Croatia.** Continental part, Alpine part, Mediterranean part.

**General distribution.** Widely distributed in Europe, including the European part of Russia (predominantly along the Ural Mountains). The species is also recorded in Turkey.

**Ecoregion.**ER5, ER11.

**Drainage.**AS, BS.

**BOLD ID.**CROTR022-19 (Continental part).

**Literature data.**Ćukušić (2019); Ivković et al. (2013, 2023); Kučinić et al. (2008, 2017b); Malicky (2014a); Neu et al. (2018); Oláh et al. (2013); Previšić et al. (2007a, 2010, 2013a); Vučković et al. (2021).

***Potamophylax pallidus* (Klapálek, 1899)**

**Distribution in Croatia.** Continental part, Alpine part, Mediterranean part.

**General distribution.** Austria, Balkan Peninsula, Croatia, Slovenia. The species is also recorded in Turkey.

**Ecoregion.**ER5, ER11.

**Drainage.**AS, BS.

**BOLD ID.**CROAA114-18 (Continental part), CROTR136-19 (Alpine part), CROTR171-19 (Alpine part), CROTR193-19 (Alpine part).

**Literature data.**Cerjanec et al. (2020); Ćukušić (2019); Ivković et al. (2023); Kučinić et al. (2010, 2017b, 2020b); Malicky (2014a); Neu et al. (2018); Previšić et al. (2007a, 2010, 2012); Šemnički et al. (2011, 2012); Vučković et al. (2021); Žalac and Kučinić (2022).

***Potamophylax rotundipennis* (Brauer, 1857)**

**Synonyms.***Stenophylax hieroglyphicus* Preudhomme de Borre, 1871; *Stenophylax pilosus* Kolenati, 1848.

**Distribution in Croatia.** Continental part, Alpine part.

**General distribution.** Austria, Balkan Peninsula, Belarus, Benelux, Czechia, Denmark, Estonia, Finland, France, Germany, Great Britain, Lithuania, Poland, Romania, Slovakia, Slovenia, Switzerland, Ukraine, including the European part of Russia.

**Ecoregion.**ER5, ER11.

**Drainage.**BS.

**BOLD ID.**CROAA016-18 (Continental part), CROTR186-19 (Alpine part).

**Literature data.**Ćukušić (2019); Ćukušić et al. (2017); Ivković et al. (2023); Kučinić et al. (2010, 2017b, 2020b); Neu et al. (2018); Previšić et al. (2007b, 2010).

***Potamophylax schmidi* Marinković-Gospodnetić, 1971**

**Distribution in Croatia.** Alpine part.

**General distribution.** Balkan Peninsula, Hungary, Slovakia.

**Ecoregion.**ER5.

**Drainage.**BS.

**BOLD ID.** No DNA barcoded specimens from Croatia.

**Literature data.**Neu et al. (2018).

#### ﻿Genus *Rhadicoleptus* Wallengren, 1891

***Rhadicoleptus alpestris* (Kolenati, 1848)**

**Synonyms.***Limnophilus ictus* Waga, 1857; *Phryganea pilosula* Zetterstedt, 1840.

**Distribution in Croatia.** Continental part, Alpine part.

**General distribution.** Four subspecies are recognised. The subspecies *Rhadicoleptus alpestris alpestris* (Kolenati, 1848) is widely distributed in Europe, including the Alpine region, Belarus, Belgium, Croatia, Czechia, Estonia, France, Germany, Great Britain, Hungary, Latvia, Lithuania, Netherlands, Poland, and the European part of Russia (along the Ural Mountains). *Rhadicoleptus alpestris macedonicus* Botoșăneanu & Riedel, 1965 is restricted to the Balkan Peninsula. *Rhadicoleptus alpestris spinifer* McLachlan, 1875 is distributed in France and Spain, while *Rhadicoleptus alpestris sylvanocarpaticus* Botoșăneanu & Riedel, 1965 occurs in Hungary, Poland, Romania, Slovakia, and Ukraine. In addition, a *Rhadicoleptus alpestris* species complex has been reported in Poland. According to the Trichoptera World Checklist (2025), the listed subspecies have the species status.

**Ecoregion.**ER5, ER11.

**Drainage.**BS.

**BOLD ID.**CROTR012-19 (Continental part), CROTR280-19 (Continental part), CROTR283-19 (Continental part), CROTR284-19 (Continental part).

**Literature data.**Ćukušić (2019); Ivković et al. (2023); Kučinić et al. (2017b); Malicky (2009); Neu et al. (2018); Previšić et al. (2010, 2013a).

#### ﻿Genus *Stenophylax* Kolenati, 1848

***Stenophylax fissus* (McLachlan, 1875)**

**Synonym.***Micropterna fuscata* Navas, 1926.

**Distribution in Croatia.** Mediterranean part.

**General distribution.** Albania, Apennine Peninsula with Sicily and Sardinia, Balkan Peninsula, Germany, Greece, Iberian Peninsula, France with Corsica, Switzerland. The species is recorded in Turkey.

**Ecoregion.**ER5.

**Drainage.**AS.

**BOLD ID.**CROTR045-19 (Mediterranean part), CROTR050-19 (Mediterranean part).

**Literature data.**Ćukušić (2019); Kučinić et al. (2017a, in press); Neu et al. (2018); Vučković et al. (2011, 2021).

***Stenophylax lateralis* (Stephens, 1837)**

**Synonym.***Halesus latipennis* Stephens, 1837.

**Distribution in Croatia.** Continental part, Alpine part.

**General distribution.** Alpine region, Bulgaria, Croatia, Czechia, Estonia, Great Britain, Hungary, Latvia, Lithuania, Poland, Romania, Scandinavian Peninsula, Slovakia, Ukraine, including the European part of Russia (near the border with Ukraine).

**Ecoregion.**ER5, ER11.

**Drainage.**BS.

**BOLD ID.**CROTR002-19 (Alpine part), CROTR021-19 (Continental part), CROTR154-19 (Alpine part), HMKKT035-10 (Alpine part), HMKKT306-10 (Alpine part), HMKKT307-10 (Alpine part), NIPM005-17 (Alpine part).

**Literature data.**Cerjanec et al. (2020); Ćukušić (2019); Gottstein Matočec et al. (2002); Ivković et al. (2023); Klapálek (1906); Kučinić and Ilić (1993); Kučinić et al. (2017a, 2017b, 2020a); Langhoffer (1912); Malicky (2009); Neu et al. (2018); Previšić et al. (2007a, 2010); Radovanović (1935); Žalac and Kučinić (2022).

***Stenophylax malaspina* (Schmid, 1957)**

**Distribution in Croatia.** Mediterranean part.

**General distribution.** Border between Northern Macedonia and Albania, Bulgaria, Greece, southern Italy, Thrace. The species is also recorded in Lebanon, Syria, and Turkey.

**Ecoregion.**ER5.

**Drainage.**AS.

**BOLD ID.** No DNA barcoded specimens from Croatia.

**Literature data.**Kučinić et al. (in press).

***Stenophylax meridiorientalis* Malicky, 1982**

**Distribution in Croatia.** Continental part, Mediterranean part.

**General distribution.** Balkan Peninsula, Crimean Peninsula, Croatia, Hungary, Poland, Ukraine, including European part of Russia (near border with Georgia). The species is recorded in Iran and Turkey.

**Ecoregion.**ER5, ER11.

**Drainage.**AS, BS.

**BOLD ID.** No DNA barcoded specimens from Croatia.

**Literature data.**Neu et al. (2018); Oláh (2010); Vučković et al. (2021).

***Stenophylax mitis* McLachlan, 1875**

**Distribution in Croatia.** Mediterranean part.

**General distribution.** Alpine region, Apennine Peninsula including Sardinia and Sicily, Balkan Peninsula, France with Corsica, Germany, Luxembourg, Netherlands, Spain. The species is also recorded in Algeria.

**Ecoregion.**ER5.

**Drainage.**AS.

**BOLD ID.**CROAA075-18 (Mediterranean part), CROTR013-19 (Mediterranean part).

**Literature data.**Ćukušić (2019); Kučinić et al. (2019c, in press); Neu et al. (2018).

***Stenophylax nycterobius* (McLachlan, 1875)**

**Synonyms.***Micropterna bofilli* Navas, 1919; *Stenophylax striatus* Kolenati, 1848; *Micropterna ventralis* Navas, 1915.

**Distribution in Croatia.** Alpine part, Mediterranean part.

**General distribution.** Alpine region, Balkan Peninsula, Belgium, Crimean Peninsula, Croatia, Czechia, France, Hungary, Luxembourg, Poland, Slovakia, Spain, Ukraine, including European part of Russia near border with Georgia (including Georgia). The species is also recorded in Lebanon and Turkey.

**Ecoregion.**ER5.

**Drainage.**AS, BS.

**BOLD ID.**CROTR055-19 (Mediterranean part), CROTR016-19 (Alpine part), CROTR044-19 (Mediterranean part), CROTR051-19 (Mediterranean part), CROTR174-19 (Mediterranean part), NIPM003-17 (Mediterranean part), NIPM006-17 (Mediterranean part).

**Literature data.**Ćukušić (2019); Gottstein Matočec et al. (2002); Ivković et al. (2023); Klapálek (1906); Kučinić and Ilić (1993); Kučinić et al. (2011, 2017a, 2017b, 2020a); Langhoffer (1912); Malicky (2014a); Neu et al. (2018); Previšić et al. (2010, 2014a); Radovanović (1935); Vučković et al. (2011, 2021); Waringer et al. (2009); Žalac and Kučinić (2022).

***Stenophylax permistus* McLachlan, 1895**

**Synonym.***Stenophylax concentricus* McLachlan, 1875.

**Distribution in Croatia.** Continental part, Alpine part, Mediterranean part.

**General distribution.** Widely distributed in Europe, including European part of Russia (near border with Georgia). The species is also recorded in Turkey and Lebanon.

**Ecoregion.**ER5, ER11.

**Drainage.**AS, BS.

**BOLD ID.**CROAA065-18 (Alpine part), CROTR048-19 (Alpine part), CROTR054-19 (Alpine part), CROTR107-19 (Alpine part).

**Literature data.**Cerjanec et al. (2020); Ćukušić (2019); Gottstein Matočec et al. (2002); Ivković et al. (2023); Klapálek (1906); Kučinić et al. (2008, 2011, 2017b, 2019c, 2020a); Langhoffer (2015); Malicky (2009, 2014a); Marinković-Gospodnetić (1979); Neu et al. (2018); Previšić et al. (2010, 2013c, 2014a); Radovanović (1935); Vučković et al. (2011, 2021); Waringer et al. (2009); Žalac and Kučinić (2022).

***Stenophylax sequax* (McLachlan, 1875)**

**Synonyms.***Micropterna affinis* Murgoci, 1951; *Micropterna lateralis* von zur Muehlen, 1880; *Micropterna taeniata* Navas, 1917.

**Distribution in Croatia.** Continental part, Alpine part, Mediterranean part.

**General distribution.** Widely distributed in Europe, including European part of Russia. The species is recorded in Turkey.

**Ecoregion.**ER5, ER11.

**Drainage.**AS, BS.

**BOLD ID.**CROTR226-19 (Alpine part), CROTR239-19 (Alpine part), NIPM004-17 (Alpine part), NIPM007-17 (Mediterranean part).

**Literature data.**Ćukušić (2019); Gottstein Matočec et al. (2002); Ivković et al. (2013, 2023); Kučinić and Ilić (1993); Kučinić et al. (2017a, 2017b, 2020a); Langhoffer (1915); Neu et al. (2018); Previšić et al. (2010, 2013c); Radovanović (1935); Vučković et al. (2021); Žalac and Kučinić (2022).

***Stenophylax testaceus* (Gmelin, 1789)**

**Synonyms.***Phryganea brunnea* Olivier, 1791; *Mesophylax impunctatus* Doehler, 1914; *Micropterna orophila* Stein, 1874.

**Distribution in Croatia.** Alpine part, Mediterranean part.

**General distribution.** Alpine region, Apennine Peninsula with Sardinia and Sicily, Balkan Peninsula, Belgium, Czechia, France, Germany, Hungary, Luxembourg, Poland, Slovakia, Spain, Corsica, Ukraine.

**Ecoregion.**ER5.

**Drainage.**AS, BS.

**BOLD ID.**CROTR026-19 (Alpine part), CROTR028-19 (Alpine part), CROTR098-19 (Mediterranean part), NIPM008-17 (Alpine part).

**Literature data.**Cerjanec et al. (2020); Ćukušić (2019); Gottstein Matočec et al. (2002); Kučinić and Ilić (1993); Kučinić et al. (2011, 2017a, 2019a, 2020a); Neu et al. (2018); Previšić et al. (2014a); Vučković et al. (2011, 2021); Waringer et al. (2009); Žalac and Kučinić (2022).

***Stenophylax vibex* (Curtis, 1834)**

**Synonyms.***Stenophylax speluncarum* McLachlan, 1875; *Stenophylax striatus flavescens* Kolenati, 1848.

**Distribution in Croatia.** Alpine part, Mediterranean part.

**General distribution.** Apennine Peninsula, Austria, Belgium, Czechia, France, Germany, Great Britain, Luxembourg, Poland, Slovakia, Spain, Switzerland. The species is also recorded in Morocco.

**Ecoregion.**ER5.

**Drainage.**AS, BS.

**BOLD ID.**CROTR285-19 (Mediterranean part).

**Literature data.**Ćukušić (2019); Gottstein Matočec et al. (2002); Ivković et al. (2023); Klapálek (1906); Kučinić et al. (2008, 2017b; 2019a); Langhoffer (1912); Malicky (2014a); Marinković-Gospodnetić (1979); Previšić et al. (2010); Radovanović (1935); Žalac and Kučinić (2022).

***Stenophylax wageneri* (Malicky, 1971)**

**Distribution in Croatia.** Mediterranean part.

**General distribution.** Albania, Italy, Montenegro.

**Ecoregion.**ER5.

**Drainage.**AS.

**BOLD ID.**NIPM001-17 (Mediterranean part), NIPM002-17 (Mediterranean part).

**Literature data.**Ćukušić (2019); Kučinić et al. (2017a, 2020a, 2021, in press); Neu et al. (2018); Žalac and Kučinić (2022).

#### ﻿﻿Family Odontoceridae Wallengren, 1891

**Genus *Odontocerum* Leach, 1815**

***Odontocerum albicorne* (Scopoli, 1763)**

**Synonyms.***Mystacide cylindrica* Pictet, 1834; *Odontocerum griseum* Leach, 1815; *Molanna albicornis incanus* Kolenati, 1859; *Crenogenes incomtus* Imhoff & Labram, 1838; *Odontocerus maculipennis* Curtis, 1834.

**Distribution in Croatia.** Continental part, Alpine part, Mediterranean part.

**General distribution.** Widely distributed in Europe.

**Ecoregion.**ER5, ER11.

**Drainage.**AS, BS.

**BOLD ID.**CROAA128-18 (Alpine), CROTR047-19 (Continental part), CROTR289-19 (Alpine part), CROTR373-21 (Mediterranean part), CROTR374-21 (Mediterranean part).

**Literature data.**Cerjanec et al. (2020); Ćukušić (2019); Ivković et al. (2023); Kučinić et al. (2011, 2017b, 2019a, 2020a, 2021); Malicky (2005a, 2014a); Marinković-Gospodnetić (1979); Neu et al. (2018); Previšić and Popijač (2010); Previšić et al. (2010, 2012, 2014a); Vučković et al. (2011, 2021); Waringer et al. (2009); Žalac and Kučinić (2022).

#### ﻿﻿Family Phryganeidae Leach, 1815

**Genus *Agrypnia* Curtis, 1835**

***Agrypnia varia* (Fabricius, 1793)**

**Synonym.***Phryganea detrita* Motschulsky, 1853.

**Distribution in Croatia.** Continental part, Alpine part, Mediterranean part.

**General distribution.** Widely distributed in Europe, including European part of Russia. The species is also recorded in Siberia and Turkey.

**Ecoregion.**ER5, ER11.

**Drainage.**AS, BS.

**BOLD ID.**CROAA013-18 (Continental part), CROTR078-19 (Alpine part), CROTR240-19 (Alpine part).

**Literature data.**Cerjanec et al. (2020); Ćukušić (2019); Ivković et al. (2023); Kučinić et al. (2011, 2017b, 2019a, 2019c, 2020a); Malicky (2009); Neu et al. (2018); Previšić et al. (2010); Žalac and Kučinić (2022).

#### ﻿Genus *Hagenella* Martynov, 1924

***Hagenella clathrata* (Kolenati, 1848)**

**Synonyms.***Neuronia clathrata* Kolenati, 1848; *Oligostomis melanoptera* Wallengren, 1880.

**Distribution in Croatia.** Continental part.

**General distribution.** Alpine region, Belarus, Benelux, Denmark, Estonia, Finland, France, Germany, Great Britain, Latvia, Lithuania, Poland, Romania, Sweeden, Ukraine, including European part of Russia. The species is also recorded in Russian Far East.

**Ecoregion.**ER11.

**Drainage.**BS.

**BOLD ID.** No DNA barcoded specimens from Croatia.

**Literature data.**Klapálek (1906); Kučinić et al. (2019a); Neu et al. (2018); Previšić et al. (2013a); Radovanović (1935).

#### ﻿Genus *Oligostomis* Kolenati, 1848

***Oligostomis reticulata* (Linneaus, 1761)**

**Synonym.***Neuronia stalii* McLachlan, 1868.

**Distribution in Croatia.** Continental part, Alpine part.

**General distribution.** Alpine region, Belarus, Belgium, Bosnia and Herzegovina, Croatia, Czechia, Estonia, France, Germany, Hungary, Lithuania, Netherlands, Poland, Scandinavian Peninsula, Slovakia, Ukraine, including European part of Russia.

**Ecoregion.**ER5, ER11.

**Drainage.**AS, BS.

**BOLD ID.**CROTR331-21 (Continental part), CROTR332-21 (Continental part), CROTR342-21 (Continental part), CROTR343-21 (Continental part), CROTR344-21 (Continental part), CROTR345-21 (Continental part), CROTR346-21 (Alpine part).

**Literature data.**Klapálek (1906); Kučinić et al. (2019a); Marinković-Gospodnetić (1979); Neu et al. (2018).

#### ﻿Genus *Oligotricha* Rambur, 1842

***Oligotricha striata* (Linnaeus, 1758)**

**Synonyms.***Phryganea analis* Fabricius, 1775; *Oligotrichia chloronevra* Rambur, 1842; *Neuronia fusca* Stephens, 1837; *Phryganea ruficrus* Scopoli, 1763.

**Distribution in Croatia.** Continental part.

**General distribution.** Widely distributed in central and north Europe (including Great Britain, Ireland), France, Balkan Peninsula, Romania, including the European part of Russia and Mongolia in Asia.

**Ecoregion.**ER11.

**Drainage.**BS.

**BOLD ID.** No DNA barcoded specimens from Croatia.

**Literature data.**Klapálek (1906).

#### ﻿Genus *Phryganea* Linnaeus, 1758

***Phryganea bipunctata* Retzius, 1783**

**Synonyms.***Phryganea beckwithii* Stephens, 1836; *Phryganea fulvipes* Burmeister, 1839.

**Distribution in Croatia.** Continental part, Alpine part.

**General distribution.** Widely distributed in Europe and European part of Russia (predominantly along the Ural Mountains), excluding Apennine, Balkan, and Iberian peninsulas. The species is also recorded in China, Mongolia, and Russian Far East.

**Ecoregion.**ER5, ER11.

**Drainage.**BS.

**BOLD ID.** No DNA barcoded specimens from Croatia.

**Literature data.**Ćukušić (2019); Ivković et al. (2023); Kučinić et al. (2017b, 2019a); Malicky (2009); Neu et al. (2018); Previšić et al. (2010).

***Phryganea grandis* Linnaeus, 1758**

**Synonyms.***Phryganea atomaria* Stephens, 1836; *Phryganea uncinata* von Paula Schrank, 1781.

**Distribution in Croatia.** Continental part, Alpine part, Mediterranean part.

**General distribution.** Widely distributed in Europe. Five subspecies are recognised, with *Phryganea grandis grandis* Linnaeus, 1758 having the widest distribution in Europe, including the European part of Russia. The subspecies *Phryganea grandis nattereri* Brauer, 1873 has been recorded in Italy. *Phryganea grandis ochrida* Malicky, 1975 is reported from the following countries: Bulgaria, Crimean Peninsula, Greece, North Macedonia, Turkey, and Ukraine. The subspecies *Phryganea grandis rotundata* Ulmer, 1905 is recorded in the European part of Russia along the Ural Mountains and in regions near the Caspian Sea, while *Phryganea grandis serti* Sipahiler, 2000 is known from a single record in Turkey. In addition, a *Phryganea grandis* species complex has been reported from Lithuania, Moldova, Montenegro, Poland, Romania, Ukraine, and the European part of Russia along the Ural Mountains. The distribution of the species and its subspecies remains indistinct, and a revision of this species group is required.

**Ecoregion.**ER5, ER11.

**Drainage.**AS, BS.

**BOLD ID.**CROAA026-18 (Continental part), CROAA134-18 (Continental part).

**Literature data.**Ćukušić (2019); Ivković et al. (2023); Klapálek (1906); Kučinić et al. (2011, 2017b, 2019a); Malicky (2009); Neu et al. (2018); Previšić et al. (2010).

#### ﻿Genus *Trichostegia* Kolenati, 1848

***Trichostegia minor* (Curtis, 1834)**

**Synonyms.***Phryganea mixta* Burmeister, 1839; *Phryganea tortriceana* Rambur, 1842.

**Distribution in Croatia.** Continental part, Alpine part, Mediterranean part.

**General distribution.** Alpine region, Belarus, Benelux, British Islands, Crimean Peninsula, Croatia, Czechia, Estonia, France, Italy, Letonia, Lithuania, Poland, Romania, Scandinavian Peninsula, Slovakia, Thrace, Ukraine, including European part of Russia.

**Ecoregion.**ER5, ER11.

**Drainage.**AS, BS.

**BOLD ID.**CROAA133-18 (Alpine part).

**Literature data.**Ćukušić (2019); Kučinić et al. (2019a, 2020a); Malicky (2009); Neu et al. (2018); Vučković et al. (2021); Žalac and Kučinić (2022).

#### ﻿﻿Family Rhyacophilidae Stephens, 1836

**Genus *Rhyacophila* Pictet, 1834**

***Rhyacophila aurata* Brauer, 1857**

**Distribution in Croatia.** Continental part, Alpine part, Mediterranean part.

**General distribution.** Alpine region, Bosnia and Herzegovina, Croatia.

**Ecoregion.**ER5, ER11.

**Drainage.**AS, BS.

**BOLD ID.**CROAA032-18 (Alpine part), CROTR145-19 (Continental part).

**Literature data.**Cerjanec et al. (2020); Ćukušić (2019); Ivković et al. (2023); Kučinić et al. (2011, 2017b); Malicky (2014a); Neu et al. (2018); Previšić and Popijač (2010); Previšić et al. (2007a, 2010); Ridl et al. (2017); Šemnički et al. (2011, 2012); Vučković et al. (2011).

***Rhyacophila balcanica* Radovanović, 1953**

**Distribution in Croatia.** Alpine part, Mediterranean part.

**General distribution.** Balkan Peninsula.

**Ecoregion.**ER5.

**Drainage.**AS, BS.

**BOLD ID.**CROTR256-19 (Alpine part), HMTRI031-08 (Mediterranean part).

**Literature data.**Ćukušić (2019); Karaouzas et al. (2015); Kučinić et al. (2011, 2020a); Malicky (2014a); Neu et al. (2018); Previšić et al. (2014a); Vučković et al. (2011, 2021); Waringer et al. (2009).

***Rhyacophila cabrankensis* Malicky, Previšić & Kučinić, 2007**

**Distribution in Croatia.** Alpine part.

**General distribution.** Croatia.

**Ecoregion.**ER5.

**Drainage.**BS.

**BOLD ID.**CROAA089-18 (Alpine part).

**Literature data.**Ćukušić (2019); Kučinić et al. (2020a); Malicky et al. (2007); Neu et al. (2018); Oláh et al. (2021).

***Rhyacophila delici* Kučinić & Valladolid, 2020**

**Distribution in Croatia.** Continental part, Alpine part, Mediterranean part.

**General distribution.** The species is not included in the Atlas, as it has been newly described (Valladolid et al. 2020). It is distributed in Croatia and Bosnia and Herzegovina (Valladolid et al. 2020).

**Ecoregion.**ER5, ER11.

**Drainage.**AS, BS.

**BOLD ID.**CROTR116-19 (Continental part), CROTR264-19 (Alpine part), GBMND8197-21 (Mediterranean part), GBMND8198-21 (Mediterranean part), GBMND8199-21 (Mediterranean part), GBMND8200-21 (Mediterranean part), GBMND8201-21 (Mediterranean part), GBMND8202-21 (Mediterranean part), GBMND8203-21 (Mediterranean part), GBMND8204-21 (Alpine part), GBMND8205-21 (Alpine part), GBMND8206-21 (Alpine part), GBMND8207-21 (Alpine part), GBMND8208-21 (Alpine part), GBMND8209-21 (Alpine part), GBMND8210-21 (Alpine part), GBMND8211-21 (Alpine part), GBMND8212-21 (Alpine part), GBMND8213-21 (Alpine part).

**Literature data.**Cerjanec et al. (2020); Ćukušić (2019); Ćukušić et al. (2017); Graf et al. (2008); Ivković et al. (2023); Karaouzas et al. (2015); Kučinić et al. (2008, 2010, 2011, 2017b, 2019a, 2020a, 2020b); Malicky (2009, 2014a); Marinković-Gospodnetić (1979); Oláh (2010); Previšić and Popijač (2010); Previšić et al. (2007a, 2010, 2012, 2013b, 2014a); Ridl et al. (2017); Valladolid et al. (2020, 2022); Vučković et al. (2011, 2021); Waringer et al. (2009); Žalac and Kučinić (2022).

***Rhyacophila dorsalis persimilis* McLachlan, 1879**

**Distribution in Croatia.** Continental part, Alpine part.

**General distribution.** Alpine region, Croatia, Czechia, Germany, Hungary, Slovakia.

**Ecoregion.**ER5, ER11.

**Drainage.**BS.

**BOLD ID.**CROAA033-18 (Alpine part), CROAA034-18 (Continental part), CROAA060-18 (Alpine part), CROAA131-18 (Continental part), CROTR198-19 (Continental part), CROTR258-19 (Continental part), CROTR263-19 (Continental part).

**Literature data.**Cerjanec et al. (2020); Ćukušić (2019); Kučinić et al. (2020a, 2020b); Neu et al. (2018); Previšić and Popijač (2010); Previšić et al. (2007b).

***Rhyacophila dorsalis plitvicensis* Kučinić & Malicky, 2002**

**Distribution in Croatia.** Alpine part, Mediterranean part.

General distribution. The subspecies is only present in Croatia.

**Ecoregion.**ER5.

**Drainage.**AS, BS.

**BOLD ID.**CROTR259-19 (Alpine part), CROTR260-19 (Alpine part), CROTR261-19 (Alpine part, CROTR262-19 (Alpine part).

**Literature data.**Ćukušić (2019); Ivković et al. (2013, 2023); Karaouzas et al. (2015); Kučinić and Malicky (2002); Kučinić et al. (2008, 2017b); Malicky (2014a); Neu et al. (2018); Previšić et al. (2007a, 2010); Šemnički et al. (2011, 2012); Vučković et al. (2021).

***Rhyacophila fasciata* Hagen, 1859**

**Synonym.***Rhyacophila septentrionis* McLachlan, 1865.

**Distribution in Croatia.** Continental part.

**General distribution.** Widely distributed in Europe, including European part of Russia (predominantly along the Ural Mountains). The species is also recorded in Turkey.

**Ecoregion.**ER11.

**Drainage.**BS.

**BOLD ID.**GBMND8225-21 (Continental part), GBMND8226-21 (Continental part).

**Literature data.**Ćukušić (2019); Neu et al. (2018); Previšić et al. (2013c); Valladolid et al. (2020).

***Rhyacophila hirticornis* McLachlan, 1879**

**Synonym.***Rhyacophila irrorella* Kolenati, 1859.

**Distribution in Croatia.** Continental part.

**General distribution.** Alpine region, Croatia, Hungary.

**Ecoregion.**ER11.

**Drainage.**BS.

**BOLD ID.**CROTR265-19 (Continental part), CROTR394-22 (Continental part), CROTR400-22 (Continental part).

**Literature data.**Ćukušić (2019); Neu et al. (2018).

***Rhyacophila laevis* Pictet, 1834**

**Synonyms.***Rhyacophila flavipes* Pictet, 1834; *Rhyacophila latipennis* Pictet, 1834; *Rhyacophila obfuscata* Pictet, 1834; *Rhyacophila slovenica* Sykora, 1963; *Rhyacophila viduata* Navas, 1918.

**Distribution in Croatia.** Continental part, Alpine part.

**General distribution.** Alpine region, Balkan Peninsula, Belgium, Crimean Peninsula, Czechia, France, Germany, Italy, Luxembourg, Ukraine.

**Ecoregion.**ER5, ER11.

**Drainage.**BS.

**BOLD ID.**CROTR266-19 (Continental part).

**Literature data.**Cerjanec et al. (2020); Ćuk and Vučković (2009); Ćukušić (2019); Klapálek (1906); Kučinić et al. (2020a); Neu et al. (2018); Previšić et al. (2012).

***Rhyacophila loxias* Schmid, 1970**

**Distribution in Croatia.** Continental part.

**General distribution.** Balkan Peninsula.

**Ecoregion.**ER11.

**Drainage.**BS.

**BOLD ID.**CROTR257-19 (Continental part), CROTR267-19 (Continental part).

**Literature data.**Ćukušić (2019); Previšić et al. (2013c).

***Rhyacophila nubila* Zetterstedt, 1840**

**Synonyms.***Rhyacophila paupera* Hagen, 1859; *Rhyacophila subnubila* Martynov, 1934; *Rhyacophila subnubila* Murgoci, 1953.

**Distribution in Croatia.** Continental part.

**General distribution.** Austria, Balkan Peninsula, Belarus, Crimean Peninsula, Czechia, Estonia, Germany, Hungary, Latvia, Lithuania, Poland, Scandinavian Peninsula, Slovakia, including European part of Russia. The species is also recorded in Armenia, Iran, Lebanon, and Turkey.

**Ecoregion.**ER11.

**Drainage.**BS.

**BOLD ID.** No DNA barcoded specimens from Croatia.

**Literature data.**Ćukušić (2019); Previšić et al. (2013c).

***Rhyacophila obliterata* McLachlan, 1863**

**Distribution in Croatia.** Continental part.

**General distribution.** Austria, Balkan Peninsula, Belgium, Croatia, Czechia, Finland, France, Germany, Great Britain, Iberian Peninsula, Luxembourg, Norway, Poland, Slovakia, Slovenia, Sweeden, Switzerland, Ukraine, including European part of Russia (along the Ural Mountains).

**Ecoregion.**ER11.

**Drainage.**BS.

**BOLD ID.** No DNA barcoded specimens from Croatia.

**Literature data.**Neu et al. (2018); Previšić et al. (2013c).

***Rhyacophila palmeni* McLachlan, 1879**

**Distribution in Croatia.** Continental part, Alpine part.

**General distribution.** Southeastern Alps and southern Balkans.

**Ecoregion.**ER5.

**Drainage.**BS.

**BOLD ID.**CROAA030-18 (Continental part).

**Literature data.**Cerjanec et al. (2020); Ćukušić (2019); Ibrahimi et al. (2012); Kučinić et al. (2015); Neu et al. (2018).

***Rhyacophila polonica* McLachlan, 1879**

**Synonyms.***Rhyacophila hageni* McLachlan, 1879; *Rhyacophila hungarica* Satori, 1938; *Rhyacophila margiti* Kiss, 2002.

**Distribution in Croatia.** Continental part, Alpine part.

**General distribution.** Balkan Peninsula, Croatia, Czechia, eastern Alps, Germany, Hungaria, Poland, Slovakia, Ukraine.

**Ecoregion.**ER5, ER11.

**Drainage.**BS.

**BOLD ID.**CROAA090-18 (Alpine part), CROTR058-19 (Continental part).

**Literature data.**Ćukušić (2019); Neu et al. (2018); Previšić et al. (2013c).

***Rhyacophila schmidinarica* Urbanič, Krušnik & Malicky, 2000**

**Distribution in Croatia.** Continental part, Alpine part.

**General distribution.** Albania, Croatia, Montenegro, Slovenia.

**Ecoregion.**ER5, ER11.

**Drainage.**AS, BS.

**BOLD ID.**CROTR007-19 (Alpine part), CROTR070-19 (Alpine part), CROTR084-19 (Alpine part).

**Literature data.**Cerjanec et al. (2020); Ćukušić (2019); Ivković et al. (2023); Kučinić et al. (2010, 2017b, 2020b); Malicky (2014a); Previšić et al. (2010, 2012, 2013c); Neu et al. (2018); Šemnički et al. (2011, 2012); Urbanič et al. (2000); Žalac and Kučinić (2022).

***Rhyacophila torrentium* Pictet, 1834**

**Synonym.***Rhyacophila valkanovi* Botosaneanu, 1956.

**Distribution in Croatia.** Alpine part.

**General distribution.** Alpine region, Bosnia and Herzegovina, France, Montenegro, Poland, Romania, Ukraine.

**Ecoregion.**ER5.

**Drainage.**BS.

**BOLD ID.**CROAA018-18 (Alpine part).

**Literature data.**Ćukušić (2019); Kučinić et al. (2020a); Vučković et al. (2011).

***Rhyacophila tristis* Pictet, 1834**

**Synonyms.***Rhyacophila angularis* Pictet, 1834; *Rhyacophila biguttata* Pictet, 1834; *Rhyacophila chesa* Navas, 1918; *Rhyacophila pelionensis* Jacquemart, 1957; *Rhyacophila umbrosa* Pictet, 1834.

**Distribution in Croatia.** Continental part, Alpine part, Mediterranean part.

**General distribution.** Alpine region, Balkan Peninsula, Belgium, Croatia, Czechia, France with Corsica, Germany, Hungary, Luxembourg, Poland, Slovakia, Ukraine. The species is also recorded in Turkey.

**Ecoregion.**ER5, ER11.

**Drainage.**AS, BS.

**BOLD ID.**CROAA098-18 (Mediterranean part), CROTR011-19 (Mediterranean part), CROTR018-19 (Continental part), CROTR031-19 (Mediterranean part), CROTR236-19 (Alpine part), CROTR250-19 (Alpine part).

**Literature data.**Ćukušić (2019); Graf et al. (2008); Ivković et al. (2013, 2023); Kučinić et al. (2017b, 2020a, 2021); Malicky (2014a); Neu et al. (2018); Oláh (2010); Previšić and Popijač (2010); Previšić et al. (2007a, 2010, 2012, 2013c); Šemnički et al. (2011, 2012); Vučković et al. (2021); Žalac and Kučinić (2022).

***Rhyacophila vulgaris* Pictet, 1834**

**Synonym.***Rhyacophila venusta* Hagen, 1859.

**Distribution in Croatia.** Continental part.

**General distribution.** Alpine region, Czechia, France, Germany, Slovakia.

**Ecoregion.**ER5.

**Drainage.**BS.

**BOLD ID.**CROAA031-18 (Continental part).

**Literature data.**Ćukušić (2019); Kučinić et al. (2015).

#### ﻿﻿Family Sericostomatidae Stephens, 1836

**Genus *Notidobia* Stephens, 1829**

***Notidobia ciliaris* (Linnaeus, 1761)**

**Synonyms.***Phryganea atra* Fourcroy, 1785; *Phryganea atrata* Fabricius, 1793.

**Distribution in Croatia.** Continental part, Alpine part, Mediterranean part.

**General distribution.** Widely distributed in Europe including European part of Russia (predominantly along the Ural Mountains).

**Ecoregion.**ER5, ER11.

**Drainage.**AS, BS.

**BOLD ID.** No DNA barcoded specimens from Croatia.

**Literature data.**Germar (1817); Ćukušić (2019); Ivković et al. (2013, 2023); Klapálek (1906); Kučinić et al. (2017b); Malicky (2014a); Neu et al. (2018); Previšić et al. (2007a, 2007b, 2010); Radovanović (1935); Šemnički et al. (2011, 2012).

#### ﻿Genus *Sericostoma* Latreille, 1825

***Sericostoma flavicorne* Schneider, 1845** / ***Sericostoma schneiderii* (Kolenati, 1848)**

**Synonym.***Prosoponia schneiderii* Kolenati, 1848 (not a syn. acc. to Malicky 2005b).

**Distribution in Croatia.** Continental part, Alpine part, Mediterranean part

**General distribution.** Alpine region, Balkan Peninsula, Belgium, Croatia, Czechia, France, Germany, Hungaria, Luxembourg, Poland, Slovakia, Spain, Ukraine. The species is also recorded in Turkey.

**Ecoregion.**ER5, ER11.

**Drainage.**AS, BS.

**BOLD ID.**CROAA058-18 (Mediterranean part), CROAA062-18 (Alpine part), CROAA115-18 (Continental part), CROTR372-21 (Mediterranean part), SERIC230-17 (Mediterranean part), SERIC231-17 (Mediterranean part), SERIC232-17 (Mediterranean part), SERIC233-17 (Mediterranean part), SERIC234-17 (Mediterranean part), SERIC235-17 (Mediterranean part), SERIC236-17 (Mediterranean part), SERIC237-17 (Mediterranean part), SERIC238-17 (Mediterranean part), SERIC239-17 (Mediterranean part), SERIC240-17 (Mediterranean part), SERIC241-17 (Mediterranean part), SERIC242-17 (Mediterranean part), SERIC243-17 (Mediterranean part), SERIC244-17 (Mediterranean part), SERIC245-17 (Mediterranean part), SERIC246-17 (Mediterranean part), SERIC247-17 (Mediterranean part), SERIC248-17 (Mediterranean part), SERIC249-17 (Mediterranean part), SERIC250-17 (Mediterranean part), SERIC251-17 (Mediterranean part), SERIC252-17 (Mediterranean part), SERIC253-17 (Mediterranean part), SERIC254-17 (Mediterranean part), SERIC255-17 (Mediterranean part), SERIC256-17 (Mediterranean part), SERIC257-17 (Mediterranean part).

**Literature data.**Cerjanec et al. (2020); Ćukušić (2019); Graf et al. (2008); Ivković et al. (2023); Klapálek (1906); Kučinić et al. (2008, 2010, 2011, 2017b, 2019c, 2020a, 2021, 2020b); Malicky (2014a); Marinković-Gospodnetić (1979); Neu et al. (2018); Oláh and Vinçon (2023); Previšić and Popijač (2010); Previšić et al. (2007a, 2010, 2012, 2013c, 2014a); Radovanović (1935); Ridl et al. (2017); Šemnički et al. (2011, 2012); Vučković et al. (2011, 2021); Waringer et al. (2009); Žalac and Kučinić (2022).

## ﻿﻿Discussion

**Figure 6.**
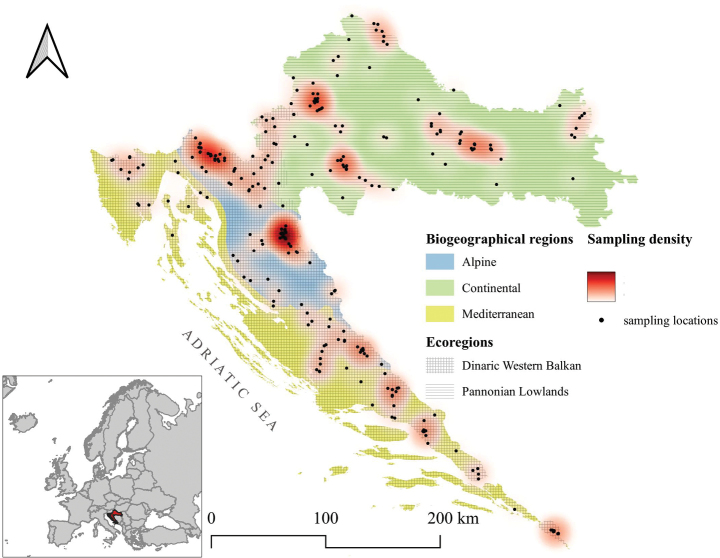
Map of Croatia showing sampling density of Trichoptera across its territory.

Based on systematic field work during the past three decades and historic Trichoptera collections, the analysis of the literature, and the BOLD Systems database, more than 150 species have been newly recorded for the Croatian fauna, some even new to science (e.g., Malicky et al. 2007; Oláh 2010, 2011; Previšić et al. 2014a; Valladolid et al. 2020). Altogether, 225 species from 18 families and 74 genera were identified (Table 1). Including five subspecies (three for *Potamophylax cingulatus* and two for *Rhyacophila dorsalis*), the total number of taxa is 228. The possibility of encountering species unknown to science remains. We analysed localities in Croatia where Trichoptera were collected (Suppl. material 1). Certain areas stand out as more researched than others, where sampling density is higher (Fig. 6): the Nature Park Papuk, the Nature Park Medvednica, areas of Međimurje and Banovina (Continental region); the upper part of the River Kupa and the National Park Plitvice Lakes (Alpine region); the River Cetina and its tributaries, the Neretva River valley and Konavle (Mediterranean region). This is due to the fact that these aquatic habitats are more interesting than others (e.g., Graf et al. 2008; Kučinić et al. 2013, 2021; Previšić et al. 2013b, 2013c, 2014a; Szivák et al. 2017; Vučković et al. 2021; Waringer et al. 2009; Žalac and Kučinić 2022). Future research on Croatian caddisfly fauna should be directed towards less investigated areas.

The analysis of caddisfly diversity and distribution primarily includes scientific studies based on adults. In the last 15 years, five species of caddisflies in the fauna of Croatia (*Beraea dira*, *Cyrnus crenaticornis*, *Ironoquia dubia*, *Rhaycophila laevis*, *Setodes viridis*) were recorded for the first time by detailed larval studies (Ćuk and Vučković 2009, 2010, 2014; Ćuk et al. 2015, 2021). Except for *C. crenaticornis* another four of these species were later recorded in the adult stages (e.g., Cerjanec et al. 2020; Ćukušić 2019). The presence of 223 species (99% of the fauna) was confirmed in the adult stage (e.g., Malicky 2009, 2014a; Kučinić et al. 2017b; Previšić et al. 2007b, 2014a; Valladolid et al. 2020; Žalac and Kučinić 2022). Only two species, *Cyrnus crenaticornis* and *Synagapetus moselyi*, have been identified in the fauna of Croatia only as larval stages that have been successfully DNA barcoded (BOLD Systems database; Ćukušić 2019; Ćuk et al. 2021).

Limnephilidae is the most species-rich family in Croatia with 66 identified species and 19 genera (Table 1). The analysis of the occurrence of families in certain regions of Croatia can be interpreted by hydrological and climatic characteristics of those regions (e.g., Bertić et al. 2001; Cerjanec et al. 2020; Ćukušić 2019; Kučinić et al. 2014; Matoničkin 1987; Matoničkin and Pavletić 1960; Vučković et al. 2021). So, for example, the family Leptoceridae is equally represented in the Continental and Mediterranean parts of Croatia, and significantly less so in the Alpine part. On the other hand, e.g., the family Rhyacophilidae is significantly less diverse in the Mediterranean compared to the Continental and Alpine regions. All families have been recorded in the Alpine region; Brachycentridae have not yet been recorded in the Mediterranean part, and Apataniidae does not occur in the Continental and Mediterranean parts (Table 1). With additional research, we expect to find these families in the Croatian regions where they have not yet been recorded.

The Mediterranean region has the lowest recorded caddisfly diversity, with 131 species, likely due to fewer freshwater habitats and fewer mountain springs and streams, which are more common in the Continental (170 species) and Alpine (155 species) regions (Table 1). Future research may reveal more species in the Mediterranean, potentially balancing species richness across regions.

A total of 77 species (34% of Croatian fauna) occur in all three regions, while 75 species (33% of Croatian caddisfly fauna) are restricted to a single region, reflecting distinct faunal patterns shaped by each region’s geology, hydrology, and habitats. This points to certain similarities (common species), but also to the specificities of each of the three geographical regions (Fig. 1) and their fauna. The distribution of each species is determined by two basic factors. In Trichoptera, on the one hand, these are primarily hydrological, climatic and geological characteristics of a certain area, and on the other hand, as with all groups of organisms, their biological characteristics (Frandsen et al. 2025; Pauls et al. 2008; Sole and Gullefors 1996; Svensson 1972).

The highest faunal similarity is between the Continental and Alpine regions, sharing 118 species (73% similarity), while the lowest is between the Continental and Mediterranean regions, with 94 shared species, representing 62% of caddisfly fauna in Croatia (Table 2). Alpine and Mediterranean regions also share 94 species, but have a higher faunal similarity of 66% (Table 2). These relatively high similarity values reflect caddisfly biology and the geographical, geological, and hydrological features of Croatian regions (Figs 1, 2, 3A–H).

The Black Sea drainage basin (Fig. 2), covering two-thirds of Croatia and 62% of its rivers (Jelić et al. 2008; Vilenica et al. 2015), hosts 203 species (90% of Croatian caddisfly fauna) due to greater aquatic habitat diversity (Fig. 3A–C, F, G). The Adriatic Sea basin, including the Mediterranean and part of the Alpine region (Fig. 1), has 141 species (63% of fauna) but lower habitat diversity, especially in the Mediterranean, explaining fewer species there.

On the Adriatic islands, only 12 caddisfly species were found (Kučinić et al. 2019c), given the limited aquatic habitats, unlike the Greece islands, which are characterised by a larger number of various habitats (Malicky 2005a). According to recent research and new distribution data (e.g., Kučinić et al. 2021; Malicky 2014a; Neu et al. 2018; Vučković et al. 2021; Žalac and Kučinić 2022) certain species, such as *Glossosoma neretvae* Marinković-Gospodnetić, 1988, *Limnephilus decipiens*, *Potamophylax luctuosus*, *Rhyacophila hirticornis*, *R. vulgaris* and *Setodes punctatus* do not occur in Dalmatia (Mediterranean Croatia) (e.g., Malicky 1979; Radovanović 1935; Vučković et al. 2021).

The Ecoregion 5 (Dinaric Western Balkan) shows significantly greater diversity of caddisflies compared to Ecoregion 11 (Pannonian Lowlands) (Fig. 1) (ER5 = 197 species, ER11 = 152 species) because it covers the karst area of Croatia (Fig. 1), which is characterised by a greater diversity of aquatic habitats in comparison to Ecoregion 11. For example, in Ecoregion 5, the National Park Plitvice Lakes is located, an area with the greatest diversity of caddisflies in Croatia (91 species) (Ivković et al. 2023; Kučinić et al. 2017b; Žalac and Kučinić 2022), and within the park there is the Labudovac locality (tufa barrier) with the largest number of recorded species of caddisflies (44 species) (Kučinić et al. 2017b, Malicky 2014a; Previšić et al. 2007a), not only in the National Park, but in Croatia in general. The karst of Croatia is the area of the greatest biodiversity (Bãnãrescu 2004), also evident in other groups of organisms, such as plants, cave fauna, aquatic Diptera, mayflies, and crayfish (Bilandžija et al. 2007; Hlaváč et al. 2020; Hlebec et al. 2023; Ivković and Plant 2015; Ivković et al. 2023; Klobučar et al. 2013; Lukić et al. 2023; Milović et al. 2021; Vilenica et al. 2015).

Some of the species, such as, *Agapetus fuscipes* Curtis, 1834, *Diplectrona felix* McLachlan, 1878, *Drusus muelleri* McLachlan, 1868, *Glossosoma boltoni* Curtis, 1834, *Hydropsyche exocellata* Dufour, 1841, *Limnephilus centralis* Curtis, 1834, *Limnephilus stigma* Curtis, 1834, *Rhyacophila evoluta* McLachlan, 1879, *Sericostoma perosnatum* (Kirby & Spence, 1826) and *Synagapetus dubitans* McLachlan, 1879, have been primarily recorded in limnological studies in larval stages (e.g., Bućan and Miliša 2023; Habdija 1979; Matoničkin 1987; Matoničkin and Pavletić 1967; Rađa and Puljas 2008; Šumanović et al. 2024; Vilenica et al. 2025). However, we chose not to include them in this study, as we have not yet confirmed their presence in the adult stage within Croatian territory or by larval DNA barcodes. We have further excluded several species that had previously been recorded in their adult stage (species that have been synonymized are not listed), such as *Anabolia nervosa* (Curtis, 1834), *Chaetopetryx mecsekensis* Nógrádi 1986, *Ecclisopteryx dalecarlica* Kolenati, 1848, *Halesus interpunctatus* (Zetterstedt, 1840), *Oxyethira tristella* Klapálek, 1865, *Glossosoma neretvae*, *Limnephilus flavospinosus* (Stein, 1874), Metalype (Psychomyia) fragilis (Pictet, 1834), *Rhyacophila philopotamoides* McLachlan, 1879, *Rhyacophila simulatrix* McLachlan, 1879, *Sericostoma pedemontanum* McLachlan, 1876, *Sericostoma personatum* (Spence in Kirby & Spence, 1826) and *Stenophylax mucronatus* McLachlan, 1880 (Cerjanec et al. 2020; Ćukušić 2019; Gottstein Matočec et al. 2002; Klapálek 1906; Langhoffer 1912; Neu et al. 2018; Previšić and Popijač 2010; Radovanović 1935; Vučković et al. 2011, 2021). Some of these species can also be expected in the fauna of Croatia in future research, e.g., *Limnephilus flavospinosus*.

Based on new data about the general distribution of those species (Neu et al. 2018) and recent faunistic and taxonomic papers (e.g., Ćukušić 2019; Kučinić et al. 2020c, 2021; Malicky 2014a, 2014b; Malicky et al. 2007; Mewis et al. 2024; Oláh 2010; Previšić et al. 2014a; Valladolid et al. 2020) some species were most likely misidentified, e.g., *Agapetus kampos* as Agapetus cf. fuscipes Curtis, 1834 (Ćukušić 2019); *A. auricollis braueri* as *Allogamus auricollis* (Ćukušić 2019; Kučinić et al. 2020a; Previšić et al. 2012); *Chaetopteryx fusca* as C. *bosniaca* (Ćukušić 2019); *Chaetopteryx gonospina* as *C. major* (Kučinić et al. 2020b); *Chaetopteryx schmidi* as species *C. mecsekensis* (Ćukušić 2019); *Chaetopteryx schmidi* as C. *papukensis* Oláh & Szivák, 2012 (Ćukušić 2019; Malicky 2014b; Oláh et al. 2012, 2015; Previšić et al. 2013c); *Chaetopteryx rugulosa* as *C. prealpensis* (Malicky 2014b; Oláh et al. 2012); *Chaetopteryx rugulosa* as *C. psunjensis* Oláh, 2015 (Ćukušić 2019; Oláh et al. 2015); *Chaetopteryx bucari* as *C. rugulosa* (Kučinić et al. 2010); *Chaetopteryx rugulosa mecsekensis* with species *C. schmidi* (Kučinić et al. 2013; Oláh 2010); *Chaetopteryx schmidi* with species *C. rugulosa* (Ćukušić 2019); *Diplectrona* sp. (Ćukušić 2019) with species *D. atra*; *Ecclisopteryx dalecarlica* with species *E. keroveci* or *E. ivkae* (e.g., Previšić and Popijač 2010; Previšić et al. 2012, 2014a; Vučković et al. 2011; Waringer et al. 2009); *Glossosoma neretvae* with species *G. discophorum* (Neu et al. 2018; Vučković et al. 2021); *Phryganea bipunctata* with species *P. grandis* (Ćukušić 2019, Kučinić et al. 2019a; specimen from River Drava); *Potamophylax depilis* Szczesny, 1994 with subspecies *P. cingulatus depilis* (Oláh et al. 2018); *Psychomyia fragilis* (Pictet, 1834) (Previšić and Popijač 2010) with species *Psychomyia klapaleki*; *Rhyacophila fasciata* with species *R. delici* (e.g., Cerjanec et al. 2020; Ćukušić 2019; Ćukušić et al. 2017; Kučinić et al. 2008, 2010, 2011, 2017b, 2019a, 2020a, 2020b; Malicky 2009, 2014; Marinković-Gospodnetić 1979; Neu et al. 2018; Oláh 2010; Previšić and Popijač 2010; Previšić et al. 2007a, 2010, 2012, 2014a; Ridl et al. 2017; Vučković et al. 2011; Waringer et al. 2009); *Rhyacophila fasciata delici* with species *R. delici* (Ivković et al. 2023; Valladolid et al. 2020, 2022; Vučković et al. 2021); *Rhyacophila dorsalis* (Curtis, 1834) (e.g., Ćukušić 2019; Kučinić et al. 2020a, 2020b) with subspecies *R. dorsalis persimilis*; *Rhyacophila praemorosa* McLachlan, 1879 with *R. polonica* (Ćukušić 2019); *Rhyacophila nubila* with subspecies *R. dorsalis persimilis* (Ćukušić 2019); *Sericostoma personatum* (e.g., Previšić and Popijač 2010; Previšić et al. 2013c; Vučković et al. 2011) with species *S. flavicorne*, *Sericostoma pedemontanum* McLachlan, 1876 (Klapálek 1906) with species *S. flavicorne*; *Sericostoma* sp. (Kučinić et al. 2010) with species *Sericostoma flavicorne*; *Setodes bulgaricus* Kumanski & Malicky, 1976 with species *S. viridis* (Cerjanec et al. 2020; Ćukušić 2019; Kučinić et al. 2015); *Silo* sp. with species *S. nigricornis* (Ćukušić 2019); *Tinodes dives* (Pictet, 1834) (e.g., Cerjanec et al. 2020; Ćukušić 2019; Ivković et al. 2013, 2023; Kučinić et al. 2008, 2016, 2017b, 2020a; Marinković-Gospodnetić 1979; Neu et al. 2018; Previšić and Popijač 2010; Previšić et al. 2007a, 2010, 2014a; Vučković et al. 2011, 2021) with subspecies *T. dives jekeeli* (Olah et al. 2021); *Triaenodes ochrellus lefkas* (Malicky 2005a; Neu et al. 2018) with species *T. lefkas* (Kučinić et al. 2020c); *Wormaldia occipitalis* (e.g., Cerjanec et al. 2020; Ćukušić 2019; Ivković et al. 2023; Kučinić et al. 2017b, 2020a, 2020b; Malicky 2009; Previšić et al. 2010, 2012, 2013c; Vučković et al. 2021; Žalac and Kučinić 2022) with species *W. subterranea* (Neu 2015).

Certain species are well-represented within the Croatian fauna and recorded from many localities (e.g., *Hydropsyche instabilis*, *Lepidostoma hirtum*, *Limnephilus lunatus*, *L. rhombicus*, *Psychomyia pusilla*, *Rhyacophila delici*, *Wormaldia subnigra*), while others are known from only a single locality of findings (e.g., *Cyrnus crenaticornis*, *Drusus chrysotus*, *Ecclisopteryx asterix*, *Hydropsyche guttata*, *H. mostarensis*, *Hydroptila vichtaspa*, *Plectrocnemia geniculata*) (Figs 4, 5), which is primarily due to the biological characteristics of certain species and the possibility of finding a larger number of habitats where they can survive (e.g., Cerjanec et al. 2020; Ćuk et al. 2021; Hickin 1967; Kladarić et al. 2021; Kučinić et al. 2015; Malicky 2009, 2014a; Wallace et al. 2003; Žalac and Kučinić 2022). For certain species such as *D. chrysotus*, *E. asterix*, and *H. mostarensis*, Croatia is on the edge of their distribution area (Figs 4, 5) (Kladarić et al. 2021; Kučinić et al. 2011; Previšić et al. 2012). The second group has a special relationship with the areas where they are found, and if possible, the preservation of their habitat from any anthropogenic impact for the purpose of their survival in our fauna is needed. For these species, it is necessary to conduct detailed research in the areas where they are found, in order to determine new localities where they also occur. For example, *D. chrysotus* is recorded only twice at the source area of the River Dobra (Cerjanec et al. 2020; Previšić et al. 2012), which had been under anthropogenic pressure (Cerjanec et al. 2020). In this specific area (Alpine region – Gorski kotar) there are more than 30 springs of various mountain streams that have not been investigated where we expect this species to occur.

We did not collect certain Trichoptera species during our research (e.g., *Hydropsyche guttata*, *Oligotricha striata*, *Parachiona picicornis*, *Platyphylax frauenfeldi*, *Triaenodes kawraiskii*) although, according to data in collections or the literature (Klapálek 1906; Malicky 2009, 2024; Malicky et al. 2002), they have been found in Croatia. These are endangered, rare species e.g., *Platyphylax frauenfeldi* (Malicky 2024; Malicky et al. 2002) (Fig. 5) or species for which Croatia is one of the limits of distribution, e.g., *Hydropscyhe guttata* (Malicky 2009) (Fig. 4). In the future, we hope to confirm their presence in the fauna of Croatia. For some species, it will be necessary to confirm their presence in some regions of Croatia, e.g., *Polycentropus ierapetra slovenica* in the Mediterranean part of Croatia or *Micrasema sericeum* in the Alpine part.

The BOLD Systems database currently contains 593 DNA-barcoded Trichoptera specimens from Croatia (Suppl. material 2). DNA barcoding encompassed 176 species (Suppl. material 2), identifying 211 BINs, covering 78% of Croatian Trichoptera fauna. Some morphologically distinct species share the same BIN (e.g., *Chaetopteryx villosa* - *C. fusca* - *C. bosniaca* – BOLD:AAA5187, *Rhyacophila nubila* - *R. dorsalis* – BOLD: AAC4103, *Limnephilus lunatus* - *L. graecus* - BOLD:AAC1503), highlighting the need for further research and the importance of a well-curated, quality-checked BOLD reference database. The accuracy of identification based on morphology is extremely important. These studies should also include analysis of nuclear genes because analysis of mitochondrial genes including DNA barcoding (COI genes) is sometimes not sufficiently informative (e.g., Darschnik et al. 2019; Weigand et al. 2017). Despite this, we believe that it is necessary to continue DNA barcoding of all segments of fauna in Croatia, including Trichoptera, as a basis for studying biodiversity, taxonomy, biomonitoring, phylogeny and phylogeography (e.g., Ćukušić 2019; Kladarić et al. 2021; Previšić et al. 2014b; Szivák et al. 2017; Valladolid et al. 2020; Waringer et al. 2009).

### ﻿﻿Notes on some caddisfly species in the Croatian fauna

#### ﻿﻿*Allogamus auricollis*braueri Kolenati, 1958 (Family Limnephilidae)

The species *Allogamus auricollis* is represented by two subspecies: *A. auricollis auricollis* (Pictet, 1834) and *A. auricollis braueri* (Kolenati, 1859) (Malicky 2004, 2016). A detailed distribution of these subspecies across Central Europe, with an in-depth morphological analysis, was presented in Malicky (2016). Molecular analyses suggest that the observed differences between the two subspecies are more indicative of interspecific rather than intraspecific variability (Malicky 2016). In contrast, a 2022 study proposed elevating the subspecies to distinct species, recognising *A. auricollis* and *A. braueri* as separate taxa (Oláh et al. 2022b). Here we recognise them as subspecies and confirm that only *A. auricollis braueri* is present in Croatia, though future research may validate its recognition as *A. braueri* at the species level.

#### ﻿﻿*Chaetopteryx schmidi* Botosaneanu, 1957 (Family Limnephilidae)

The genus *Chaetopteryx* has been recorded in the fauna of Croatia with nine species (e.g., Malicky 1996; Malicky and Krušnik 1988; Malicky 2014b; Kučinić et al. 2013, 2020b; Oláh 2011; Szivák et al. 2017). Within the Papuk and Psunj mountains (Continental part - eastern Croatia) and Kozara mountain (north Bosnia and Herzegovina), the species *C. papukensis* was described as part of the *C. rugulosa* species complex (*Chaetopteryx schmidi* species subgroup) (Oláh et al. 2012). In the same study, the species *Chaetopteryx prealpensis* (*C. rugulosa* species complex) was described as a new species, distributed in the western part of continental Croatia and Austria, Hungary, and Slovenia (Oláh et al. 2012). However, Malicky (2014b) synonymised *C. papukensis* with *C. schmidi*, a species originally described from Romania (Botosaneanu 1957). In addition to significant morphological similarities, Malicky pointed out that a molecular analysis (DNA barcoding - COI gene) was conducted on specimens from the Đerdap region on the Romanian - Serbian border, confirming their belonging to *C. schmidi* with those from Papuk. The results revealed no molecular differences among the analysed specimens or populations, supporting the conclusion that they represent a single species, *C. schmidi* (Malicky 2014b). This is supported also by the paper in which species *Chaetopteryx bucari* (Kučinić et al. 2013) was described, where DNA-barcoded specimens from Đerdap in Serbia and Romania (Cerna Valley) showed a high similarity, belonging to the same species, *C. schmidi* (Ćukušić 2019; Kučinić et al. 2013; Szivák et al. 2017). Also, the specimens of *C. schmidi* from Croatia (Papuk) and Serbia (Đerdap) share the same BIN, BOLD:ACF1416 (BOLD Systems). In the same study, Malicky synonymised *C. prealpenis* with *C. rugulosa* (Malicky 2014b), and we agree with these results.

In 2015, a new species of the genus *Chaetopteryx*, *C. psunjensis*, was described from the Psunj Mountain, located near Papuk (Oláh et al. 2015). This species was identified based on two specimens, one male and one female (Oláh et al. 2015). In the same study, the distribution area of the species *C. papukensis* was reduced to only the area of Papuk Mountain in Croatia, and a new species *C. balcanica* was described in the distribution area of that species in northern Bosnia and Herzegovina and Serbia (Đerdap region). The distribution area of *C. schmidi* is limited only to the part of Romania from where this species was originally described (Botosaneanu 1957).

Species identification within genus *Chaetopteryx*, particularly within the *C. rugulosa* complex, requires great caution due to high intraspecific morphological variability (Kučinić et al. 2013; Malicky 2014b; Olah et al 2012). In a doctoral dissertation (Ćukušić 2019), DNA barcoding and analysis of data from the BOLD database confirmed that specimens from Psunj in Croatia share the same BINs (BOLD: AAP3303) with some specimens from Papuk and Medvednica mountains in Croatia and specimens from the Pohorje mountains in Slovenia. These specimens belong to the species *Chaetopteryx rugulosa*. These data also show that in Papuk we have both species *C. rugulosa* and *C. schmidi*, and at Psunj mountain the species *Chaetopteryx rugulosa*. According to molecular data from specimens from Đerdap (Ćukušić 2019; Kučinić et al. 2013), we believe that the species *C. balcanica* must be synonymised with the *C. schmidi* and also we think that *C. psunjensis* is not a valid species. To determine the relationship between the species from Papuk and Psunj with other species of the genus *Chaetopteryx*, it is necessary to conduct a study with additional molecular markers. There is a possibility that the populations from this area (Papuk and Psunj), due to certain morphological specificities, may be classified as subspecies of the species *C. schmidi* and *C. rugulosa*. The same principle may also apply to to some other taxa of the genus *Chaetopteryx* from the *C. rugulosa* group (e.g., *C. rugulosa prealpenis*) and the *C. schmidi* group (e.g., *C. schmidi balcanica*). Taxonomic and phylogenetic relationships within the genus *Chaetopteryx* in Central Europe are very complex (e.g., Ćukušić 2019; Szivák et al. 2017) and require detailed morphological and molecular analyses.

#### ﻿﻿*Drusus chrysotus* (Rambur, 1842) (Family Limnephilidae)

The genus *Drusus* is particularly intriguing for biological research, encompassing morphology, taxonomy, phylogeny, phylogeography, and conservation biology (e.g., Marinković-Gospodnetić 1971; Pauls et al. 2008; Previšić et al. 2014b; Vitecek et al. 2017, 2020; Waringer et al. 2015). Species within this genus inhabit springs and the upper reaches of mountain streams, leading to population fragmentation and speciation processes (Marinković-Gospodnetić 1979; Oláh et al. 2017; Pauls et al. 2008; Previšić et al. 2014b; Vitecek et al. 2015).

*Drusus chrysotus* is the rarest species of this genus in Croatia, recorded at only one locality in the Alpine part of Croatia (Cerjanec et al. 2020; Previšić et al. 2012) (Fig. 5). Its population is highly isolated from others. DNA barcode analysis indicates that this Croatian population constitutes a distinct genetic lineage, differing by more than 5% from other European populations of the species (Ćukušić 2019). This finding suggests that the Croatian population may belong to a separate, sister species - *Drusus lepos* Oláh, 2017, described recently (Oláh et al. 2017) and potentially distributed in Croatia’s central mountain regions. Future research should investigate additional nearby springs, particularly around the source of the Dobra River basin.

#### ﻿﻿*Hydropsyche guttata* Pictet, 1834 (Family Hydropsychidae)

*Hydropsyche guttata* has been recorded in Croatia solely from specimens housed in the Varaždin Municipal Museum collection. These specimens were collected nearly a century ago, on 29 June 1927 and 17 June 1929, in Varaždin, northwestern Croatia (Malicky 2009) (Fig. 4). The study highlights that these findings represent the southernmost records of this species in Europe, significantly extending its known range. In neighboring Hungary, *H. guttata* has only been documented at two localities, while there are no records from Slovenia, despite its common occurrence in Austria (Malicky 2009).

Northern Croatia likely marks the southern boundary of *H. guttata* distribution. However, no further records of the species have emerged in more than 30 years of research, despite extensive Trichoptera surveys in the region. The two historical specimens remain the only confirmed records to date. Future research in the continental northern part of Croatia may yet confirm the species continued presence.

#### ﻿﻿*Platyphylax frauenfeldi* (Brauer, 1857) (Family Limnephilidae)

*Platyphylax frauenfeldi* is one of the most endangered caddisfly species globally. Historically inhabiting large European rivers, it has suffered severe population declines due to habitat destruction and is now potentially restricted to the Drava and Mura rivers along the Hungarian - Croatian border (Malicky 2009, 2024; Malicky et al. 2002) (Fig. 5). One of the last confirmed specimens was collected in the Mura River, Murčak locality, northwestern Croatia, on 12 October 1999 (Malicky 2024; Malicky et al. 2002).

The pressing question remains: does this species still persist in the European fauna, or is it functionally extinct? The last documented specimen is from 2001 (Malicky 2024). Addressing this question requires extensive surveys of aquatic habitats in northwestern Croatia and Hungary, with the hope of confirming *P. frauenfeldi* as a rare but extant species rather than an extinct one. Protecting these freshwater ecosystems is critical to ensuring the survival of *P. frauenfeldi* in Croatia and Europe.

#### ﻿﻿*Polycentropus ierapetra slovenica* Malicky, 1998 (Family Polycentropodidae)

This taxon was described from Slovenia in 1998 (Malicky 1998) as a subspecies of *P. ierapetra*Oláh et al. (2022a) changed the status of all subspecies of the *P. ierapetra* complex (Malicky 2004; Sipahiler 1989) to the species level. *P. ierapetra slovenica* is distributed only in the territory of Slovenia and Croatia and is isolated from other taxa of this complex, distributed in the territories of Greece, Bulgaria, Turkey, and Lebanon on one hand, and the Iberian Peninsula on the other (e.g., Malicky 2004; Neu et al. 2018). We are of the opinion that prior to the taxonomic revision of this subspecies complex, morphological and detailed molecular studies should be carried out, and accordingly, we retain the status of *P. ierapetra slovenica* at the subspecies level.

#### ﻿﻿*Potamophylax cingulatus* (Stephens, 1837) and *Potamophylax latipennis* (Curtis, 1834) (Family Limnephilidae)

The species *Potamophylax cingulatus* has been recorded in Croatia at three locations with three subspecies: one in the Continental northwestern region on Mount Ivanščica, *P. cingulatus alpinus* (Ćukušić 2019) and two in the Alpine region: one at the source of the Gacka River, *P. cingulatus depilis* (Oláh et al. 2018) and another in the western part of the Alpine region of Croatia (Neu et al. 2018), Gorski kotar region, *P. cingulatus cingulatus*. The species *P. cingulatus* exhibits significant morphological variability, leading to the description of several subspecies (Malicky 2004). Oláh et al. (2018) elevated all these subspecies to species rank, a possibility previously considered by Szczęsny (1990). In the Trichoptera World Checklist (2025), *P. cingulatus alpinus* and *P. cingulatus depilis* also have species status, *P. alpinus* and *P. depilis*. While this approach has some justification, future taxonomic studies should incorporate molecular methods to validate these classifications. Our research on populations from northwestern Croatia, coupled with DNA barcode analysis, indicates that the Croatian populations group with other European populations (e.g., Finland, Norway, Germany, Spain) (Ćukušić 2019). We propose that subspecies status remains more appropriate for now. Ćukušić (2019) noted that specimens from the source regions of rivers Gacka, Dretulja, Rječina, and Una (Alpine region, Ecoregion 5) and Zrmanja (Mediterranean region, Ecoregion 5) form a distinct genetic lineage within *P. cingulatus*, potentially representing the subspecies *P. cingulatus depilis*. However, Automatic Barcode Gap Discovery (ABGD) analysis did not separate them as distinct species. These specimens were preliminarily identified morphologically as *P. latipennis*, highlighting taxonomic ambiguities regarding the relationship between *P. cingulatus* and *P. latipennis* (Ćukušić 2019), at least for certain populations.

#### ﻿﻿*Rhyacophila balcanica* Radovanović, 1953 (Family Rhyacophilidae)

*Rhyacophila balcanica* is among the largest species of the genus *Rhyacophila* within the Croatian fauna (Malicky 2004). It was originally described from specimens collected in Montenegro (Radovanović 1953), and has since been recorded in both the Alpine and Mediterranean regions of Croatia (Ćukušić 2019; Karaouzas et al. 2015; Kučinić et al. 2011, 2020a; Vučković et al. 2021).

Oláh et al. (2022a) established the *Rhyacophila balcanica* species complex, which includes three additional species: *Rhyacophila albanica* Oláh & Ibrahimi, 2022, *R. montenegra* Oláh, 2022 and *R. syrikaltera* Oláh & Ibrahimi, 2022 (Oláh et al. 2022a). However, none of these species have yet been confirmed within the Croatian territory.

#### ﻿﻿*Rhyacophila cabrankensis* Malicky, Previšić & Kučinić, 2007 (Family Rhyacophilidae)

*Rhyacophila cabrankensis* is an endemic species of Croatia, known exclusively from the Čabranka River in the Alpine region (Malicky et al. 2007) (Fig. 5). Morphologically, it closely resembles *R. vulgaris*, a species found in the more northern karstic areas of Croatia (Fig. 5), and their distribution follows an allopatric pattern. DNA barcode analysis indicates a genetic divergence of 1.8% between these two species (Ćukušić 2019), which is notably lower than the intraspecific variation observed within *R. vulgaris* itself (Ćukušić 2019). It is likely that *R. cabrankensis* represents an isolated genetic lineage derived from *R. vulgaris* (Ćukušić 2019). Unlike its widely distributed relative, *R. cabrankensis* is restricted to a highly localised, isolated, spring-fed section of the Čabranka River. This geographic isolation is a key factor contributing to its speciation, resulting in both morphological and molecular distinctiveness.

#### ﻿﻿*Rhyacophila dorsalis dorsalis* McLachlan, 1879 and *Rhyacophila dorsalis plitvicensis* Kučinić & Malicky, 2002 (Family Rhyacophilidae)

The subspecies *R. dorsalis plitvicensis* was first described from specimens collected in the National Park Plitvice Lakes (Kučinić and Malicky 2002). A single specimen found in the Cetina River (Vučković et al. 2021), after more than 15 years of research in the area, suggests that this occurrence may be the result of passive transport from Plitvice Lakes rather than an established population. Notably, field studies were sometimes conducted at Plitvice Lakes immediately before surveys in the Cetina region.

Another subspecies, *R. dorsalis persimilis*, is found in the western regions of Croatia. The relationship between these two subspecies is strictly allopatric, with stable morphological differences in male genital structures (phallus, dorsal lobe, and lateral lobe), that allow a clear differentiation (Kučinić and Malicky 2002).

#### ﻿﻿*Sericostoma flavicorne* Schneider, 1845 and *Sericostoma schneideri* Kolenati, 1845 (Family Sericostomatidae)

The relationship among the three species: *Sericostoma flavicorne*, *S. personatum*, and *S. schneideri* is very interesting. For now, only the taxonomical status of *S. personatum* is definite, which has so far not been recorded in Croatian fauna. Relationships between the two other taxa are very complex and are still a subject of various interpretations, since they had been described in the first half of the 19^th^ century. The species *S. flavicorne* was described by Schneider in 1845, based on specimens collected in Turkey, without any illustrations of the imago (Schneider 1845). Drawings of this species from the type locality were published only 150 years later (Sipahiler 2000). The species *S. schneideri* was described by Kolenati three years after the description of *S. flavicorne*, probably from the type locality in Dalmatia (Mediterranean part, Croatia) (Kolenati 1948).

Botosaneanu (2001) argued that *S. flavicorne* described from Anatolia, Turkey, and *S. flavicorne* recorded from specimens in Dalmatia, probably represent a distinct species. Based on morphological illustrations from that study and the study of Sipahiler (2000) and Oláh and Vinçon (2023), Croatian specimens would correspond to *S. schneideri*, exhibiting minimal variability in male genital structures. Malicky had a different approach, synonymising the species *S. schneideri* with *S. flavicorne* (Malicky 2004) but in a paper from 2005, he lists both species (Malicky 2005b), considering that only molecular analyses will help distinguish them, indicating that Central European populations of the genus *Sericostoma* cannot currently be reliably identified (Malicky 2005b).

A 2023 study suggested that *S. schneideri* is a valid species, inhabiting part of central and southern Europe, and that *S. flavicorne* is absent from Europe, with a distribution in Asia (Oláh and Vinçon 2023). Morphological analysis of specimens collected in Croatia (in the Alpine and Mediterranean regions) shows a strong similarity with *S. schneideri* from Oláh and Vinçon (2023), as well as with the drawings in Botosaneanu (2001).

Darschnik et al. (2019) published sequences through which species *Sericostoma personatum* and *S. flavicorne* can be distinguished, stating that authors of the study are not sure if it is *S. flavicorne* or *S. schneideri*. The authors showed that in this case, DNA barcoding is not very helpful, but other molecular analyses should also be included. According to those results, we consider that the same analysis should be done for *S. flavicorne* specimens from its type locality in Turkey (Sipahiler 2000) and compared to Darschnik et al. (2019). After all is said, we are almost certain that such research will confirm *S. schneideri* as a European species, being also the only *Sericostoma* species in Croatia. This hypothesis can only be confirmed by future molecular and morphological research in Asia and Europe (Malicky 2005b).

#### ﻿﻿*Setodes viridis* (Fourcroy, 1785) (Family Leptoceridae)

Two species from the genus *Setodes* occur in Croatia: *Setodes punctatus* (Fabricius, 1793) and another taxon, which has certain features of *S. bulgaricus* and *S. viridis* (Cerjanec et al. 2020). The species *Setodes bulgaricus* has been described as a subspecies of *Setodes viridis*, *S. viridis bulgaricus* (Kumanski and Malicky 1976). Schmid (1987) changes the subspecies status of *S. viridis bulgaricus* to the species level, *S. bulgaricus* (Schmid 1987). Our DNA barcoding data showed no difference between our two DNA barcoded specimens and three specimens in the BOLD database (same BINs; BOLD: ACB1895), one larva from Germany and two adults from Iraq, which belong to the species *Setodes viridis*. Cerjanec et al. (2020) suggested that the subspecies *Setodes viridis bulgaricus* might need reconsideration. However, after a more detailed analysis, we are inclined to the view that two species of the genus Setodes are currently present in the Croatian fauna: *Setodes viridis* and *S. punctatus*. We consider *S. bulgaricus* a valid species, but it has not yet been recorded in the fauna of Croatia.

#### ﻿﻿*Stenophylax malaspina* (Schmid, 1957) (Family Limnephilidae)

The species *Stenophylax malaspina* was recently identified as a new addition to the Croatian fauna, collected in Central Dalmatia within the Mediterranean region (Kučinić et al. 2025, in press).

Its occurrence in Croatia represents the westernmost point of its known distribution. The nearest known population of *S. malaspina* is in southern Italy about 400 km to the south (Neu et al. 2018). While such range expansions are not uncommon, Croatia serves as either the eastern or western border for several caddisfly species, e.g., *Rhyacophila nubila* (Previšić et al. 2013b), *R. vulgaris* (Kučinić et al. 2015), *R. balcanica* (Karazaous et al. 2015), *Hydropsyche mostarensis* (Kučinić et al. 2011), *Ecclisopteryx asterix* (Kladarić et al. 2021), *Triaenodes lefkas* (Kučinić et al. 2020c), and *Drusus vespertinus* (Kučinić et al. 2014).

Dalmatia, Croatia’s largest Mediterranean region, has been extensively surveyed, with long-term studies on rivers such as the Cetina, Krka, Grab, Neretva, and Ljuta (e.g., Malicky 2014a; Vučković et al. 2021; Waringer et al. 2009; Žalac and Kučinić 2022). The absence of *S. malaspina* from these well-researched locations is surprising, and its occurrence in Central Dalmatia remains unexplained. Further studies involving broader regional sampling and molecular analyses may provide new insights into its distribution and biogeographical history in Croatia.

#### ﻿﻿*Tinodes dives jeekeli* Botosaneanu, 1980 (Family Psychomyiidae)

The subspecies *Tinodes dives jeekeli* was originally described from the Plitvice Lakes National Park in the Alpine region of Croatia (Botosaneanu 1980). Its taxonomic status has changed over time: in the first edition of the Atlas of European Trichoptera (Malicky 1983), it retained its subspecies status, but in the second edition (Malicky 2004), it was classified as a valid species, *Tinodes dives*. However, Oláh et al. (2021) revised its taxonomy once more, recognising *jeekeli* as a distinct species: *Tinodes jeekeli* Botosaneanu, 1980.

Morphological characteristics of the male genitalia, which are stable across all analysed Croatian populations, along with DNA barcode analysis (Ćukušić 2019), indicate specific traits within these populations. However, these differences do not warrant species-level distinction, but rather support a subspecies classification, thus retaining the taxon as *Tinodes dives jeekeli* Botosaneanu, 1980. *Tinodes dives jeekeli* is not a rare subspecies in Croatia and has been recorded at numerous sites in both Alpine and Mediterranean regions.

#### ﻿﻿*Wormaldia subterranea* Radovanović, 1932 (Family Philopotamidae)

*Wormaldia subterranea* was first described by Radovanović in 1932, based on specimens collected from the Podpeška Cave in Slovenia (Radovanović 1932). It is a rare occurrence for a new Trichoptera species to be discovered and described from a cave environment. Initially classified as a subspecies - *W. occipitalis subterranea* (Kimmins 1953) - it was later synonymised with *Wormaldia occipitalis* (Botosaneanu 1989). However, a 2015 study on the *W. occipitalis* group conducted additional morphological analyses, demonstrating clear morphological distinctions between *W. occipitalis* and *W. subterranea*, thereby confirming them as separate species (Neu 2015). Genetic research further supports this differentiation (Mewis et al. 2024).

Neu (2015) and Mewis et al. (2024) also provide a detailed distribution map of *W. occipitalis* and *W. subterranea*, confirming that only *W. subterranea* occurs in Croatia. Consequently, all previous data referring to *W. occipitalis* in Croatian collections and literature must be revised (e.g., Cerjanec et al. 2020; Kučinić et al. 2017a, 2020a; Malicky 2009; Previšić et al. 2013b; Vučković et al. 2021).

## ﻿﻿Conclusion

This study presents the first comprehensive overview of the Trichoptera fauna of Croatia. Given the increasing anthropogenic pressures, particularly habitat destruction and the effects of climate change, documenting the current state of the fauna is crucial not only for monitoring ongoing ecological changes but also for improving our understanding of species distribution and taxonomic status, which in turn supports their effective conservation.

Future research involving long-term monitoring, comprehensive DNA barcoding, and the integration of these data into the BOLD database will deepen our understanding of Croatian Trichoptera diversity. It is also essential to enter accurate morphological determinations of taxa into the BOLD Systems. Ongoing work on Croatian caddisfly collections, the description of previously undescribed larvae, and accurate data entry of barcoded specimens into BOLD Systems are crucial steps.

## ﻿﻿Additional information

### Use of AI

No use of AI was reported.

### Author ORCIDs

Mladen Kučinić https://orcid.org/0000-0003-2756-2305

Ana Previšić https://orcid.org/0000-0002-0332-7522

Anđela Ćukušić https://orcid.org/0009-0000-4130-3241

Ivan Vučković https://orcid.org/0000-0001-6627-8898

Sanja Žalac https://orcid.org/0009-0005-0513-554X

Darko Cerjanec https://orcid.org/0000-0001-6795-1589

Renata Ćuk https://orcid.org/0000-0002-6863-5227

Katarina Z. Stojanović https://orcid.org/0000-0002-1064-792X

Nazymgul Akimbekova https://orcid.org/0000-0001-6889-6606

Marijana Vuković https://orcid.org/0009-0001-1806-6134

Josip Skejo https://orcid.org/0000-0002-2554-4499

Dora Hlebec https://orcid.org/0000-0001-5967-864X

Aleksandar Božić https://orcid.org/0009-0000-1857-9055

Hrvoje Kutnjak https://orcid.org/0000-0003-1498-6977

### Data availability

All of the data that support the findings of this study are available in the main text or Supplementary Information.

## Supplementary materials

﻿Supplementary material 1 Localities for collected Trichoptera from Croatia in the last three decades. Data type xlsx https://binary.pensoft.net/file/1489058This dataset is made available under the Open Database License (http://opendatacommons.org/licenses/odbl/1.0/). The Open Database License (ODbL) is a license agreement intended to allow users to freely share, modify, and use this Dataset while maintaining this same freedom for others, provided that the original source and author(s) are credited.Mladen Kučinić, Ana Previšić, Anđela Ćukušić, Ivan Vučković, Sanja Žalac, Darko Cerjanec, Renata Ćuk, Katarina Z. Stojanović, Nazymgul Akimbekova, Marijana Vuković, Josip Skejo, Dora Hlebec, Aleksandar Božić, Hrvoje Kutnjak

﻿Supplementary material 2 Data on localities, geocoordinates, and geographical regions for DNA barcoded specimens in BOLD database from Croatia Data type xlsx https://binary.pensoft.net/file/1489059This dataset is made available under the Open Database License (http://opendatacommons.org/licenses/odbl/1.0/). The Open Database License (ODbL) is a license agreement intended to allow users to freely share, modify, and use this Dataset while maintaining this same freedom for others, provided that the original source and author(s) are credited.Mladen Kučinić, Ana Previšić, Anđela Ćukušić, Ivan Vučković, Sanja Žalac, Darko Cerjanec, Renata Ćuk, Katarina Z. Stojanović, Nazymgul Akimbekova, Marijana Vuković, Josip Skejo, Dora Hlebec, Aleksandar Božić, Hrvoje Kutnjak
